# N6-methyladenine-mediated aberrant activation of the lncRNA SOX2OT-GLI1 loop promotes non-small-cell lung cancer stemness

**DOI:** 10.1038/s41420-023-01442-w

**Published:** 2023-05-06

**Authors:** Hongliang Dong, Lili Zeng, Weiwei Chen, Qian Zhang, Fei Wang, Yan Wu, Bingjie Cui, Jingjing Qi, Xin Zhang, Cuilan Liu, Jiong Deng, Yong Yu, Clemens A. Schmitt, Jing Du

**Affiliations:** 1 Medical Research Center, Binzhou Medical University Hospital, 256600 Binzhou, P. R. China; 2 Department of Oral and Maxillofacial Surgery, Binzhou Medical University Hospital, 256600 Binzhou, P. R. China; 3 Department of Pathology, Binzhou Medical University Hospital, 256600 Binzhou, P. R. China; 4 Department of Oncology, Binzhou Medical University Hospital, 256600 Binzhou, P. R. China; 5 Johannes Kepler University, Altenbergerstraße 69, 4040 Linz, Austria; 6 Department of Hematology, Binzhou Medical University Hospital, 256600 Binzhou, P. R. China; 7 Kepler University Hospital, Department of Hematology and Oncology, Krankenhausstraße 9, 4020 Linz, Austria; 8 Charité-Universitätsmedizin, Freie Universität Berlin, Humboldt-Universität zu Berlin, and Berlin Institute of Health, Medical Department of Hematology, Oncology and Tumor Immunology, and Molekulares Krebsforschungszentrum – MKFZ, Campus Virchow Klinikum, 13353 Berlin, Germany; 9 Max-Delbrück-Center for Molecular Medicine in the Helmholtz Association, Robert-Rössle-Straße, 1013125 Berlin, Germany; 10 Deutsches Konsortium für Translationale Krebsforschung (German Cancer Consortium), Partner site Berlin, Berlin, Germany

**Keywords:** Cancer stem cells, Long non-coding RNAs

## Abstract

Despite the advent of precision medicine and immunotherapy, mortality due to lung cancer remains high. The sonic hedgehog (SHH) cascade and its key terminal factor, glioma-associated oncogene homolog 1 (GLI1), play a pivotal role in the stemness and drug resistance of lung cancer. Here, we investigated the molecular mechanism of non-canonical aberrant GLI1 upregulation. The SHH cascade was upregulated in stem spheres and chemo-resistant lung cancer cells and was accountable for drug resistance against multiple chemotherapy regimens. GLI1 and the long non-coding RNA SOX2OT were positively regulated, and the GLI1-SOX2OT loop mediated the proliferation of parental and stem-like lung cancer cells. Further mechanistic investigation revealed that SOX2OT facilitated METTL3/14/IGF2BP2-mediated m6A modification and stabilization of the *GLI1* mRNA. Additionally, SOX2OT upregulated METTL3/14/IGF2BP2 by sponging miR-186-5p. Functional analysis corroborated that GLI1 acted as a downstream target of METTL3/14/IGF2BP2, and GLI1 silencing could block the oncogenicity of lung cancer stem-like cells. Pharmacological inhibition of the loop remarkably inhibited the oncogenesis of lung cancer cells in vivo. Compared with paired adjacent normal tissues, lung cancer specimens exhibited consistently upregulated GLI1/SOX2OT/METTL3/14/IGF2BP2. The m6A-modified GLI1-SOX2OT loop may serve as a potential therapeutic target and prognostic predictor for lung cancer therapy and diagnosis in the clinic.

## Introduction

Among malignant diseases, lung and bronchus-derived cancers remain the leading cause of mortality in both men and women [1]. Resistance to multiple interventions and distal metastasis of lung cancer neoplastic cells need to be urgently addressed. Cancer stem cells (CSCs) have been extensively studied and proved to account for multidrug resistance and metastasis in various cancer types [2]. Therefore, mechanistic studies aiming to illustrate the molecular machinery required to initiate and maintain the malignant behavior of CSCs may pave the way for the final eradication of CSCs and improve the therapeutic efficacy for lung cancer.

The sonic hedgehog (SHH) pathway is a key signaling cascade regulating self-renewal and differentiation of stem cells. It is aberrantly upregulated in various tumor types, including lung, breast, colon, brain, and skin cancers, contributing to the initiation and progression of malignant diseases [3]. Essential components of the SHH pathway include the transmembrane proteins patched (PTCH), smoothened (SMO), and the terminal signaling regulator-transcription factor glioma-associated oncogene 1 (GLI1). In pathological situations such as cancer, the SHH ligand binds to the PTCH receptor to release SMO and stimulate the translocation of GLI1 into the nucleus in a canonical SMO-dependent way. Hyperactive GLI1 then triggers its downstream stemness-associated targets, including GLI1, ABCG2, and Snail, to propel multidrug resistance and immune cell invasion [4, 5]. SHH pathway components are genetically altered in approximately 14% of non-small-cell lung cancer (NSCLC) cases, canonically activating the signaling [6]. Additionally, non-canonical GLI1 activation leads to resistance to SMO inhibitor therapy. GLI1 is activated in >70% of lung adenocarcinoma cases through MAPK/ERK signaling in an SMO-independent non-canonical manner [7]. In lung squamous cell carcinoma, PI3K promotes tumorigenesis via GLI1 activation [8]. We have previously demonstrated that GLI1 is overexpressed in lung cancer specimens and negatively correlated with the prognosis of patients [9]. Further investigations dissecting non-canonical SMO-independent mechanisms initiating the GLI1 signaling may disclose new therapeutic targets and serve as a novel strategy to overcome SMO inhibitor resistance.

Long non-coding RNAs (lncRNAs), a cluster of untranslated RNAs harboring >200 nucleotides, play a crucial role in gene regulation [10]. The lncRNA sex-determining region Y-box 2 (SOX2)-overlapping transcript (SOX2OT), located at chromosome 3q26.3 of the human genome, is closely related to various tumorigenesis pathways [11]. SOX2OT is positively correlated to the stage of lung cancer and indicates poor survival [12–14]. Compared with healthy populations, patients with lung cancer exhibit higher SOX2OT content in the plasma and exosomes, which drops significantly following surgery [15–17]. In vitro and in vivo studies have proved that SOX2OT regulates the proliferation and differentiation of CSCs and is involved in the multidrug resistance and metastasis of cancer cells [11]. The stem cell-specific transcription factor SOX2 is located at the intron of SOX2OT and is positively regulated by SOX2OT [18]. LncRNAs can regulate target gene expression at distinct levels, including transcriptional, post-transcriptional, translational, and post-translational levels. Recent studies have found that SOX2OT promotes GLI1 expression via histone H3 methylation and acetylation, leading to resistance to multiple therapeutic strategies in lung cancer [19]. High-throughput next-generation sequencing has revealed that N6-methyladenine (m6A) is the most abundant RNA modification in eukaryotic cells and plays a pivotal role in the post-transcriptional regulation of gene expression. M6A modification exerts its function via readers (IGF2BPs, METTL3/14) and erasers (FTO, ALKBH5) [20, 21]. SOX2OT was reported to sensitize glioblastoma cells to temozolomide therapy via ALKBH5-mediated demethylation and stabilization of SOX2 [22]. We have demonstrated that SOX2 can upregulate GLI1 expression, and SOX2-targeted intervention abolishes GLI1-mediated stemness of lung cancer cells [23]. Whether SOX2OT regulates GLI1 expression at the post-transcriptional level and is mutually regulated by GLI1 remains to be investigated.

In this study, we investigated how SOX2OT forms a positive loop with the GLI1 signaling cascade through modulation at transcriptional and post-transcriptional levels. On one hand, GLI1 stimulated SOX2OT transcription, acting as a positive transcription factor. On the other hand, SOX2OT promoted transcription of METTL3/14/IGF2BP2 and guided the protein products to GLI1 RNA for m6A modification. The m6A-modified GLI1-SOX2OT loop may serve as a potential therapeutic target and prognostic predictor for patients with lung cancer in a clinical setting.

## Results

### Key components of the SHH cascade are aberrantly upregulated in lung cancer cells, stemness spheroids, and chemotherapy-resistant cell lines

**Fig. 1:**
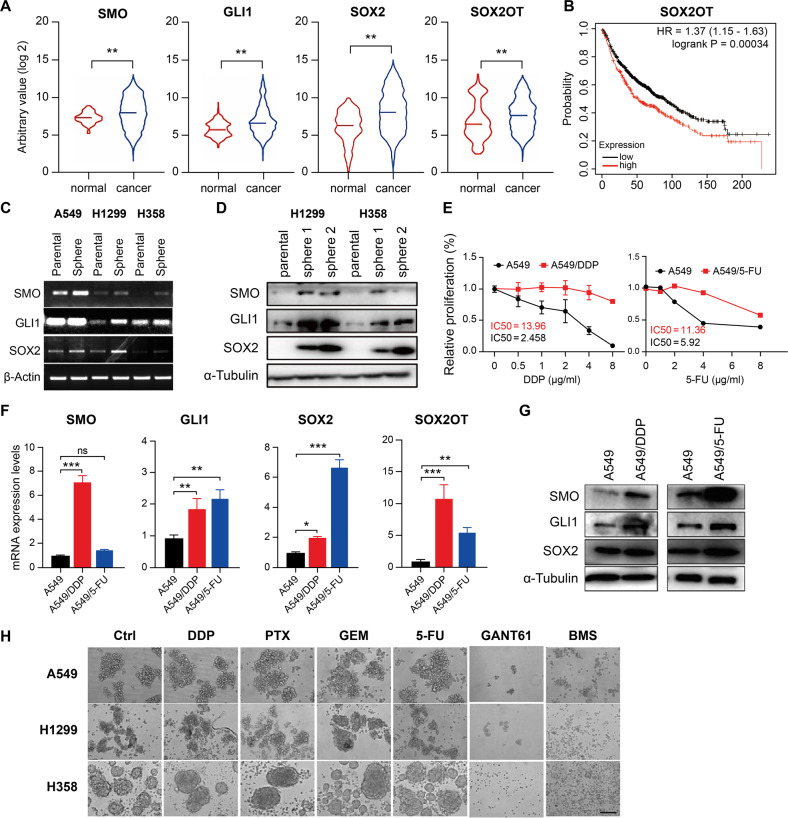
Lung cancer specimens, stem-like cells, and drug-resistant cells showed overactivation of the SHH/SOX2 cascade and overexpression of the lncRNA SOX2OT. **A** Transcription levels of SMO, GLI1, SOX2, and SOX2OT in lung cancer specimens and paired adjacent normal tissues. Plots of SMO and SOX2 were generated using data obtained from the University of California Santa Cruz genome browser. GLI1 and SOX2OT transcript data were obtained from the Gene Expression Database of Normal and Tumor Tissues 2 (GENT2). **B** Overall survival curve of patients with lung cancer based on SOX2OT expression levels as generated by Kaplan–Meier Plotter (*n* = 1880). **C** Aberrant transcriptional profile of SHH components and stemness transcriptional factor SOX2 in spheroids compared to NSCLC parental cells. **D** Overexpression of the SHH pathway and SOX2 in NSCLC spheroids detected using immunoblotting. **E** DDP- or 5-FU-resistance index in A549 drug-resistant cells compared to parental cells measured using CCK8 assay. **F** RT-qPCR analysis of the SHH/SOX2 cascade and SOX2OT in parental and DDP/5-FU-resistant A549 cells. **G** Immunoblotting of SHH pathway components and SOX2 in parental and DDP/5-FU-resistant A549 cells. **H** NSCLC spheroids are refractory to multiple chemotherapeutic drugs and sensitive to GANT61 (GLI1 inhibitor) and BMS (SMO inhibitor). Drugs were applied at the following doses: DDP, 8 µg/mL; PTX, 0.1 µg/mL; GEM, 1 µg/mL; 5-FU, 4 µg/mL; GANT61 (GLI inhibitor), 40 µM; BMS, 10 µM. Magnification = ×200, scale bar = 100 μm. DDP cisplatin, PTX paclitaxel, GEM gemcitabine, 5-FU 5-fluorouracil.

We performed data mining using the University of California Santa Cruz genome browser and Gene Expression Database of Normal and Tumor Tissues 2 (GENT2) and confirmed overexpression of SMO, GLI1, SOX2, and SOX2OT in lung cancer samples (Fig. 1A). The correlation of SMO, GLI1, and SOX2 expression with poor prognosis in lung cancer has been demonstrated [9, 23]. The overall survival curve of lung cancer patients generated by the Kaplan–Meier Plotter showed that high SOX2OT expression level is related to unfavorable prognosis (Fig. 1B). Lung cancer stem-like cells propagated in spheroids and immunofluorescence experiments indicated that SOX2 was remarkably overexpressed in spheroids compared with those in corresponding parental cells (Supplementary Fig. S1A). RT-qPCR and immunoblotting revealed that SMO, GLI1, and SOX2 were more actively transcribed and translated in lung cancer spheroids than in parental cells (Fig. 1C, D). To validate whether the SHH cascade was also stimulated in drug-resistant cells, we used commercially available DDP- or 5-FU-resistant A549 cells as models. DDP- and 5-FU-resistant cells had a resistance index of 5.7 and 1.9, respectively (Fig. 1E). RT-qPCR and immunoblotting displayed that compared with parental A549 cells, chemotherapy-insensitive cells exhibited upregulated SMO, GLI1, SOX2, and SOX2OT (Fig. 1F, G). Three different types of lung cancer spheroids were treated with drugs according to the half minimal inhibitory concentration (IC_50_) (Supplementary Fig. S1B), and the sphere formation assay indicated that spheroids were resistant to the four commonly used first-line chemotherapy drugs DDP, paclitaxel (PTX), gemcitabine (GEM), and 5-FU, and sensitive to inhibitors against GLI1 or SMO (Fig. 1H). Additionally, compared with parental cells, CSCs isolated from spheroids harbored a significantly higher capacity to initiate solid tumor formation in vivo, corroborating the stemness (Supplementary Fig. S1C).

### GLI1-SOX2OT forms a regenerative loop to positively regulate the proliferation of lung cancer parental and stem cells

**Fig. 2:**
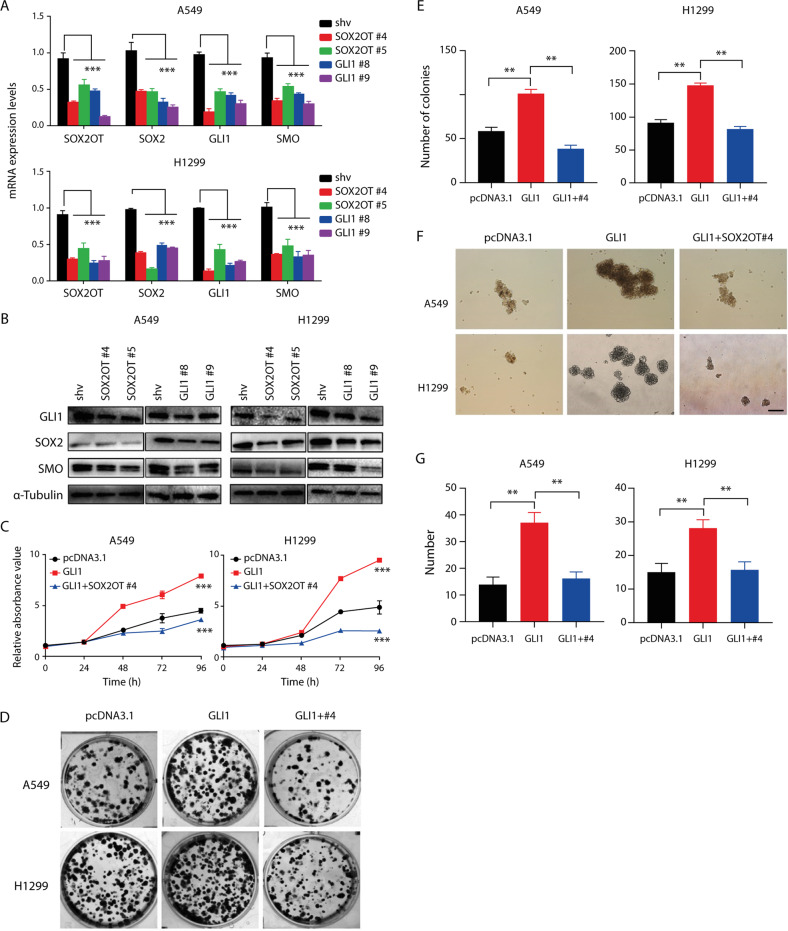
SHH pathway components and SOX2OT reciprocally regulate each other. **A** mRNA expression of *SOX2OT* and SHH pathway members upon SOX2OT or GLI1 knockdown detected using RT-qPCR. **B** Expression of the key components of the SHH pathway and downstream SOX2 upon SOX2OT or GLI1 knockdown detected via immunoblotting. **C** Proliferative capability of NSCLC cells upon enforced GLI1 expression or double transfection with shRNA against SOX2OT measured using CCK8 assay. **D**, **E** Clonal formation assay and histogram of NSCLC cells upon GLI1 overexpression or SOX2OT interference in parallel. **F**, **G** Spheroid formation and histogram of NSCLC cells transfected with GLI1-overexpressing and/or SOX2OT shRNA plasmid. Magnification = ×100, scale bar = 200 μm.

As SOX2 was positively modulated by its host gene *SOX2OT* in a cis-acting manner (Supplementary Fig. S2A) [23], we further investigated how SOX2OT interplays with GLI1 and functions in lung cancer stemness. Genetic manipulation of SOX2OT and GLI1 with shRNA knockdown vectors was used for molecular profiling and phenotype analysis. RT-qPCR and immunoblotting displayed SMO/GLI1/SOX2 downregulation upon SOX2OT knockdown at transcriptional and translational levels (Fig. 2A, B). In contrast, SOX2OT/SOX2/SMO expression at RNA and protein levels was also repressed upon GLI1 disruption (Fig. 2A, B). Knockdown efficacy of SOX2OT/GLI1 validated via RT-qPCR revealed that SOX2OT#4 and GLI1#9 silenced the target gene efficiently and thus were used for the following experiments (Fig. 2A). Meanwhile, both SOX2OT and GLI1 repression significantly attenuated sphere formation of lung cancer cells (Supplementary Fig. S2B/C). Cell proliferation detected via growth curve and clonal formation assay revealed that forced GLI1 expression promoted NSCLC cell propagation while SOX2OT repression blocked the pro-proliferative effect of GLI1 (Fig. 2C–E). Meanwhile, spheroid formation assay and quantification exhibited that SOX2OT-knockdown/GLI1-overexpression double transfection induced NSCLC stem cell proliferation (Fig. 2F, G). Fluorescent in situ staining indicated that enhanced green fluorescent protein (EGFP)-tagged GLI1-overexpressing lung cancer cells had a stronger SOX2OT signal (Supplementary Fig. S2D). These data suggested that SOX2OT acted as a downstream factor and formed a feed-forward loop with GLI1 to modulate the stemness of NSCLC cells.

### GLI1 acts as a transcription factor and activates SOX2OT transcription through promoter binding

**Fig. 3:**
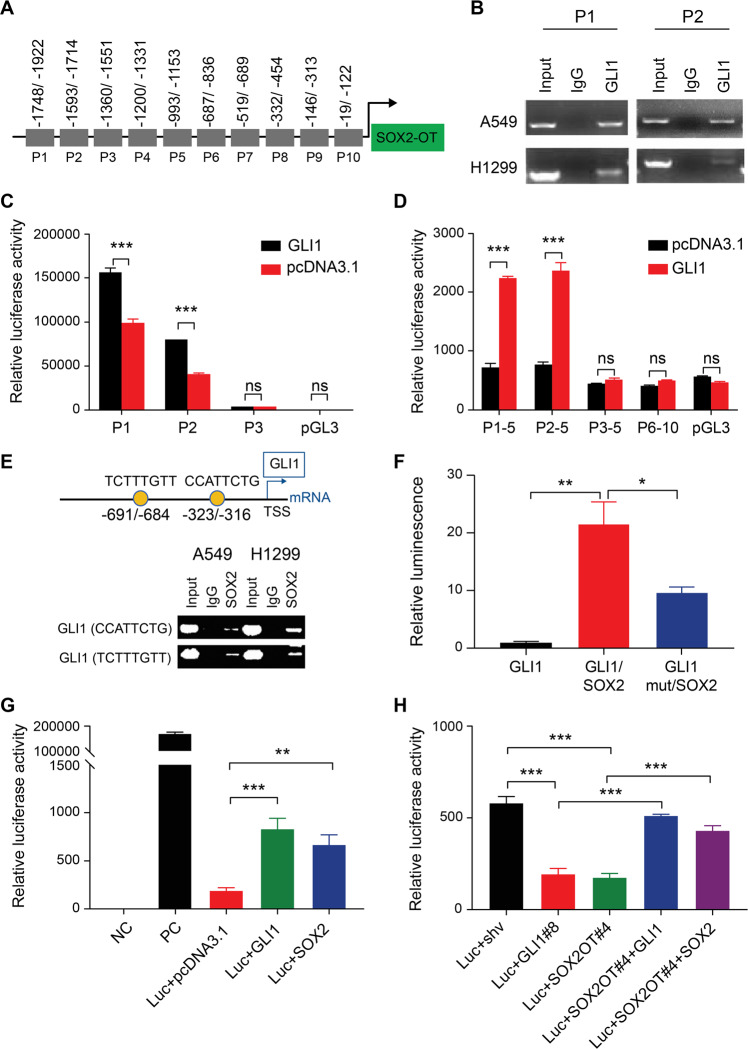
GLI1 acts as a transcription factor to initiate SOX2OT transcription by directly interacting with its promoter. **A** Diagram illustrating primer design for the amplification of the SOX2OT promoter. **B** ChIP demonstrated that GLI1 binds to the P1-P2 sequence upstream of the transcription start site of SOX2OT. **C** Luciferase assay displayed specific stimulation of the P1/P2 sequence compared to P3 and empty vector backbone in 293T cells. **D** Luciferase assay validated selective activation of the P1-5/P2-5 but not the P3-5/P6-10 sequence corresponding to the SOX2OT promoter by GLI1 in 293T cells. **E** JASPAR prediction and ChIP validation of the binding motifs of SOX2 on GLI1 promoter. **F** Luciferase assay of SOX2 binding to wild-type and mutated GLI1 promoter sequences in 293T cells. **G** Luciferase assay of GLI1-responding elements upon exogenous GLI1 or SOX2 overexpression. **H** Luciferase assay in 293T cells upon SOX2OT knockdown or double transfection with GLI1- or SOX2-overexpressing plasmids.

We investigated whether GLI1 promotes SOX2OT transcription, as previously reported for SOX2 [24]. Ten pairs of primers spread across 2000 bp upstream of the transcription start site (TSS) at the SOX2OT promoter were designed (Fig. 3A). ChIP revealed that GLI1 bound to the promoter was amplified using P1 and P2 primers (Fig. 3B). Next, sequences amplified using P1/P2 and P3 as negative controls were subcloned into the pGL3 luciferase reporter vector. Luciferase assay after GLI1/pcDNA3.1 co-transfection and various promoters driving the pGL3 reporter plasmids demonstrated that GLI1 activated the P1 and P2 regions, driving luciferase expression (Fig. 3C). Even without exogenous GLI1 transfection, P1 and P2 promoter sequences demonstrated stronger activity compared to P3 and the blank control (Fig. 3C). To further confirm if the functional binding sites were located within the P1-P2 sequences, P1-5/P2-5 sequences, which covered P1-P5 or P2-P5 separately, and P3-5 and P6-10 sequences as negative controls were subcloned into the pGL3 vector. Luciferase reporter assay revealed that GLI1 triggered luciferase activity via P1-5 and P2-5 rather than P3-10, confirming the direct binding of GLI1 to 1922–1593 bp upstream of the TSS at the SOX2OT promoter (Fig. 3D). Additionally, the transcription factor-binding site predicted by JASPAR revealed that there were two interaction sequences of SOX2 at 691 and 323 bp upstream of the TSS at the GLI1 promoter and the binding was authenticated using ChIP (Fig. 3E). Luciferase reporter assay proved that enforced SOX2 expression remarkably promoted wild-type GLI1 promoter activity while mutation of target sequences (CCATTCTG) significantly decreased SOX2-stimulated GLI1 promoter activity (Fig. 3F). Further luciferase reporter assay using the pGM-GLI1 vector demonstrated that both GLI1 and SOX2 transfection significantly induced exogenous GLI1 promoter activity (Fig. 3G). shRNA targeting GLI1 or SOX2OT remarkably decreased exogenous GLI1 promoter activity, and this phenomenon was counterbalanced by the enforced expression of GLI1 or SOX2 in parallel (Fig. 3H). RT-qPCR demonstrated that GLI1 or SOX2 overexpression remarkably stimulated the SMO/GLI1/SOX2OT/SOX2 axis in lung cancer cells (Supplementary Fig. S3A, B). Collectively, these data showed that GLI1 acted as a transcription factor and stimulated the transcription of both SOX2 and SOX2OT.

### *GLI1* mRNA m6A modification by METTL3/14 and IGF2BP2 accounts for its persistent activation

**Fig. 4:**
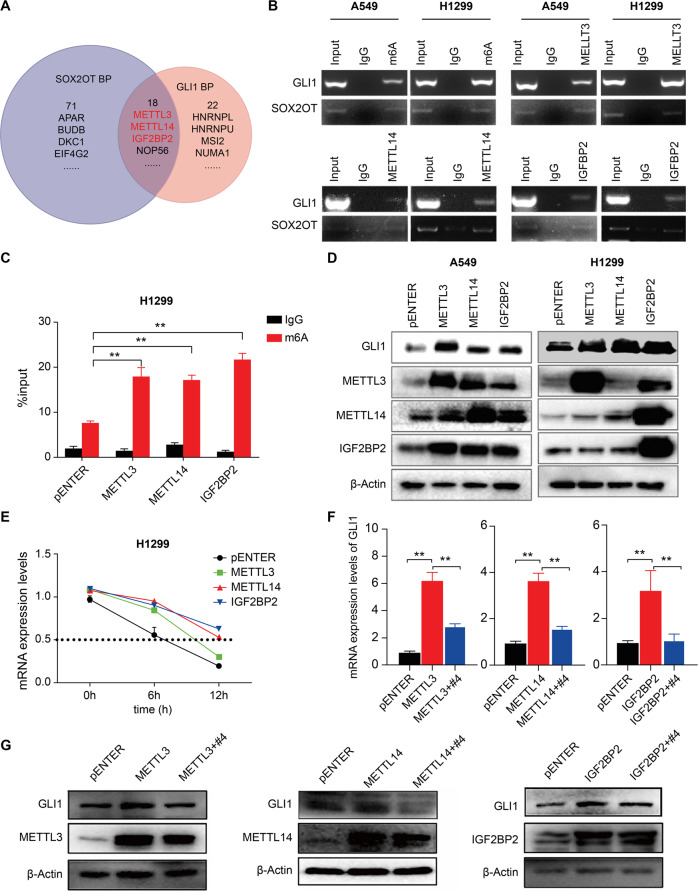
SOX2OT-dependent m6A modification and stabilization of *GLI1* mRNA by METTL3/14 and IGF2BP2. **A** Proteins interacted with both *GLI1* mRNA and SOX2OT were predicted using StarBase. **B** RIP assay demonstrated that SOX2OT and *GLI1* mRNA were enriched for m6A modification and interacted with METTL3/14/IGF2BP2. **C** RIP-PCR validated that METTL3/14/IGF2BP2 overexpression promoted m6A modification of *GLI1* mRNA. **D** METTL3/14/IGF2BP2 upregulation promoted GLI1 expression detected via immunoblotting. **E** RNA stability assay revealed that METTL3/14/IGF2BP2 overexpression increased *GLI1* mRNA stability compared to the pENTER backbone. **F** Expression of *GLI1* mRNA upon METTL3/14/IGF2BP2 overexpression or co-transfection with SOX2OT shRNA. **G** Expression of GLI1 at the protein level upon METTL3/14/IGF2BP2 overexpression or co-transfection with SOX2OT shRNA.

The above data illustrated that GLI1 acted as an upstream modulator to directly regulate SOX2OT transcription, and how SOX2OT modulates GLI1 expression to form the positive loop remains to be elucidated. After cytoplasmic/nuclear RNA purification, SOX2OT is located mainly in the cytoplasm of A549 and H1299 lung cancer cells (Supplementary Fig. S4A). LncRNA in cytoplasm usually acts as a scaffold to recruit target proteins or as competing endogenous RNAs (ceRNA) to absorb miRNA and protect the degradation of target RNAs [10]. Our database search did not reveal any miRNA that bridges SOX2OT and GLI1 directly. Therefore, we investigated the proteins that could bind to both *GLI1* mRNA and SOX2OT using StarBase. Eighteen proteins were found to interact with both molecules, and three of them were RNA m6A modifiers (Fig. 4A). Among them, METTL3/14 were two key components forming a complex responsible for m6A writing, and IGF2BP2 and HNRNPC for m6A reading. A high expression of the IGF2BP family proteins, including IGF2BP2, showed a remarkable association with poor overall survival of patients with lung cancer [25]. Hence, we select METTL3/14 and IGF2BP2 to further validate how m6A modification influences the GLI1/SOX2OT loop. RIP assay coupled with PCR confirmed the m6A modification of GLI1/SOX2OT and the direct interaction between GLI1/SOX2OT and METTL3/14 or IGF2BP2 in NSCLC cells (Fig. 4B). In addition, METTL3/14 or IGF2BP2 overexpression significantly enhanced m6A modification of the *GLI1* mRNA compared to that of the vector backbone in H1299 cells (Fig. 4C). The expression of GLI1 at the protein level was accordingly increased upon METTL3/14/IGF2BP2 overexpression in two NSCLC cell lines (Fig. 4D). The mechanism of how m6A modification influences GLI1 expression was then investigated through RNA stability, translation initiation, and protein degradation. RNA stability assay with RT-qPCR revealed that all three m6A modifiers counteracted dactinomycin-induced RNA degradation, particularly IGF2BP2 (Fig. 4E). In line with this, the protein degradation assay followed by immunoblotting revealed that all three m6A modifiers halted cycloheximide-induced protein degradation ratio, particularly IGF2BP2 (Supplementary Fig. S4B). To determine whether the protein stabilization effect of m6A was due to an increased number of mRNA molecules or more efficient protein synthesis, the binding effect of *GLI1* mRNA with ribosomal protein L22 (RPL22), a component of the 60S subunit of cytoplasmic ribosomal protein, was detected. First, exogenous RPL22 expression in H1299 cells was validated via immunoblotting (Supplementary Fig. S4C). RIP assay revealed that m6A modification induced by METTL3/14 or IGF2BP2 did not increase the interaction between *GLI1* mRNA and RPL122, suggesting that m6A modification was not involved in the initiation of *GLI1* mRNA translation (Supplementary Fig. S4D). To verify the role of SOX2OT in m6A-mediated post-transcriptional stabilization of GLI1, H1299 cells were co-transfected with SOX2OT shRNA and METTL3/14/IGF2BP2 overexpression vector. Detection of GLI1 expression at the protein level confirmed that SOX2OT knockdown effectively counteracted METTL3/14/IGF2BP2-mediated increase in *GLI1* mRNA expression (Fig. 4F, G). Meanwhile, SOX2OT silencing produced only minor effects on exogenous METTL3/14/IGF2BP2 transcript levels, according to RT-qPCR analysis (Supplementary Fig. S4E). These data suggested that SOX2OT might act as a scaffold to guide METTL3/14/IGF2BP2 for half-life-extending modification of GLI1 transcripts.

### GLI1 suppression reverses METTL3/14/IGF2BP2-induced lung cancer stemness

**Fig. 5:**
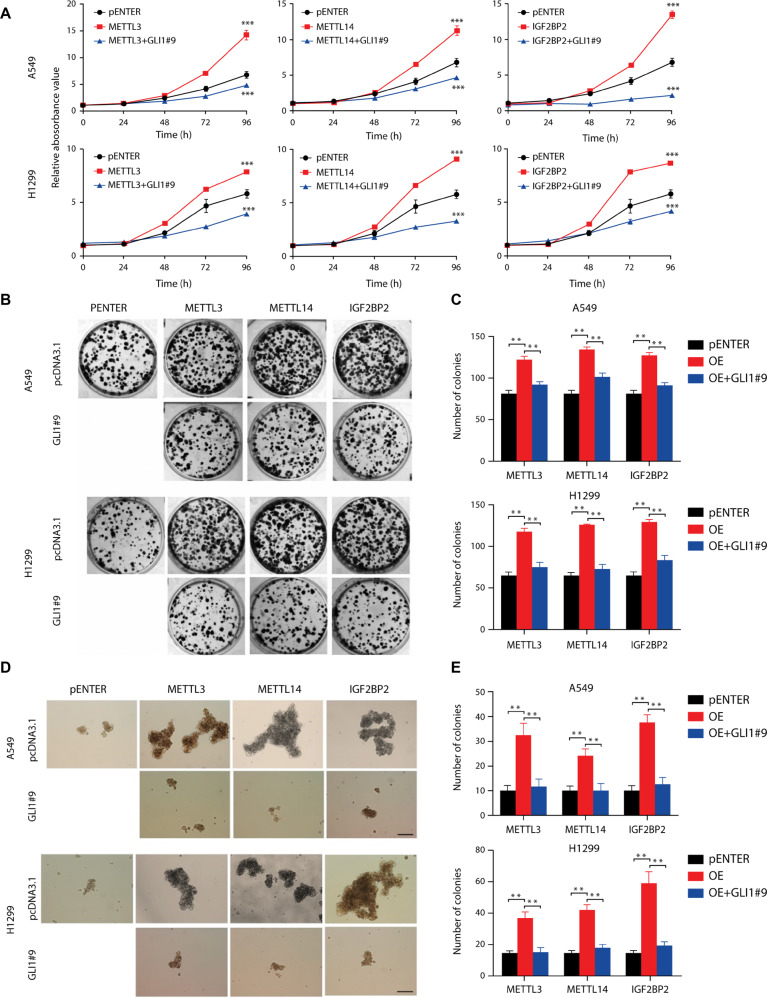
METTL3/14/IGF2BP2-mediated proliferation of NSCLC stem cells upon GLI1 repression. **A** Proliferation assay of NSCLC cells transfected with METTL3/14/IGF2BP2 and/or GLI1 shRNA. **B** and **C** Spheroid formation assay and histogram of NSCLC cells transfected with METTL3/14/IGF2BP2 and/or GLI1 shRNA. **D** and **E** Clonal formation assay and histogram of NSCLC cells transfected with METTL3/14/IGF2BP2 and/or GLI1 shRNA. Magnification = ×100, scale bar = 200 μm.

We then investigated whether three m6A modifiers investigated in this study propel cellular proliferation through GLI1 using functional analysis. CCK8 and clonal formation assay revealed that METTL3/14 and IGF2BP2 overexpression promoted lung cancer and stem cell proliferation compared to the pENTER empty vector backbone. Co-transfection with the GLI1 knockdown plasmid in parallel blocked the pro-proliferative effect of exogenous METTL3/14 and IFG2BP2 on NSCLC cells (Fig. 5A–C). Spheroid formation assay and the established histogram corroborated that GLI1 repression disrupted METTL3/14/IGF2BP2-stimulated propagation of lung cancer cells (Fig. 5D, E). These data suggested that GLI1 acted as a downstream modulator of METTL3/14 and IFG2BP2 to regulate the malignant behavior of lung cancer cells.

### GLI1 positively regulates endogenous METTL3/14 and IGF2BP2 expression via SOX2OT

**Fig. 6:**
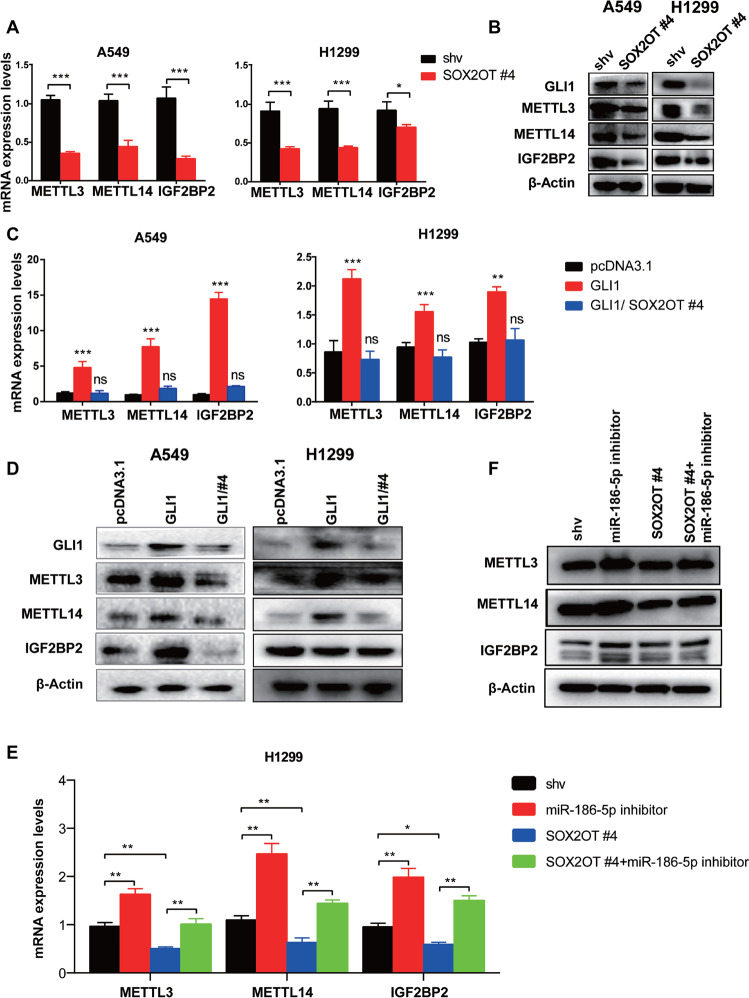
GLI1 positively regulates the expression of METTL3/14/IGF2BP2 via SOX2OT. **A**, **B** METTL3/14/IGF2BP2 expression at the mRNA and protein level in NSCLC cells upon SOX2OT knockdown, detected using RT-qPCR and immunoblotting, respectively. **C**, **D** Upregulation of METTL3/14/IGF2BP2 promoted by GLI1 transfection was blocked by SOX2OT suppression measured via RT-qPCR and immunoblotting. **E**, **F** miR-186-5p inhibitor promoted the expression of METTL3/14/IGF2BP2 and counteracted their downregulation upon SOX2OT interference detected via RT-qPCR and immunoblotting.

To fully elucidate the m6A-modified GLI1-SOX2OT network, we explored whether METTL3/14/IGF2BP2 is mediated by GLI1 or SOX2OT. RT-qPCR and immunoblotting revealed that SOX2OT suppression significantly reduced the endogenous expression of METTL3/14 and IGF2BP2 at mRNA and protein levels (Fig. 6A, B). GLI1 transfection also enhanced the expression of METTL3/14 and IGF2BP2, whereas SOX2OT suppression offset the stimulation of METTL3/14 and IGF2BP2 expression by GLI1 (Fig. 6C, D). This suggested that GLI1 indirectly regulated endogenous METTL3/14 and IGF2BP2 expression through SOX2OT. Data mining using StarBase predicted the common miR-186-5p to bridge SOX2OT and METTL3/14/IGF2BP2 (Supplementary Fig. S5). RT-qPCR and immunoblotting demonstrated that an miR-186-5p inhibitor significantly increased the expression of METTL3/14/IGF2BP2 in H1299 cells and rescued METTL3/14/IGF2BP2 downregulation upon SOX2OT knockdown (Fig. 6E, F). Therefore, SOX2OT might promote the transcription of METTL3/14/IGF2BP2 by sponging miR-186-5p.

### Repression or m6A modification of GLI1 attenuates tumorigenesis of lung cancer cells in vivo

**Fig. 7:**
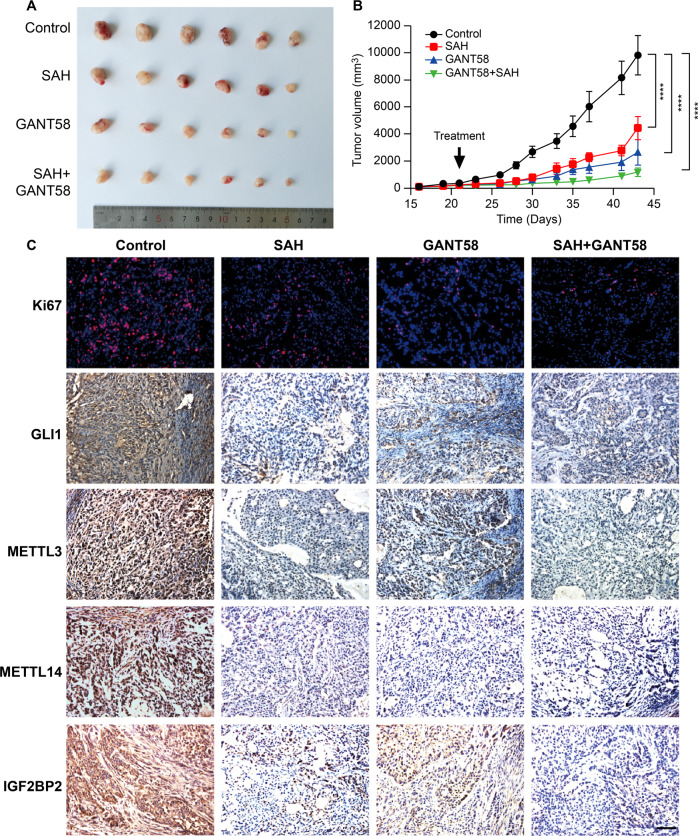
Pharmacological intervention of GLI1 and METTL3 attenuates lung cancer tumor growth in vivo. **A** Tumor bulk in mice subcutaneously implanted with A549. **B** Tumor growth curves in groups treated with GANT58, SAH, or their combination. The control group was treated with saline. **C** IHC staining of Ki67, GLI1, METTL3/14, and IGF2BP2 in treatment and control groups. Scale bar = 100 µM.

Therapeutic intervention was performed by injection of GLI1 specific inhibitor GANT58, METTL3 inhibitor SAH, or their combination in nude mice implanted with A549 subcutaneously. Administration of GANT58 or SAH significantly retarded tumor growth, and combined administration further diminished tumor propagation in mice with a neglectable effect on body weight (Fig. 7A, B, Supplementary Fig. S6A–C). Ki67 staining of tumor specimens demonstrated that cellular proliferative capability was repressed in the treatment group (Fig. 7C). RT-PCR results showed that transcriptional levels of the GLI1-SOX2OT loop, METTL3/14, and IGF2BP2 were significantly downregulated by both GLI1 and METTL3 inhibitor (Supplementary Fig. S6D). Immunohistochemistry staining of GLI1, METTL3/14, and IGF2BP2 also confirmed that inhibitors against GLI1 or METTL3 decreased protein levels of GLI1, METTL3/14, and IGF2BP2 in tumor samples (Fig. 7C). These results validated that repression of the GLI1-SOX2OT loop robustly prohibited tumor growth in lung cancer mouse model.

### GLI1-SOX2OT loop and relevant m6A modifiers were upregulated in human lung cancer specimens compared to adjacent normal tissues

**Fig. 8:**
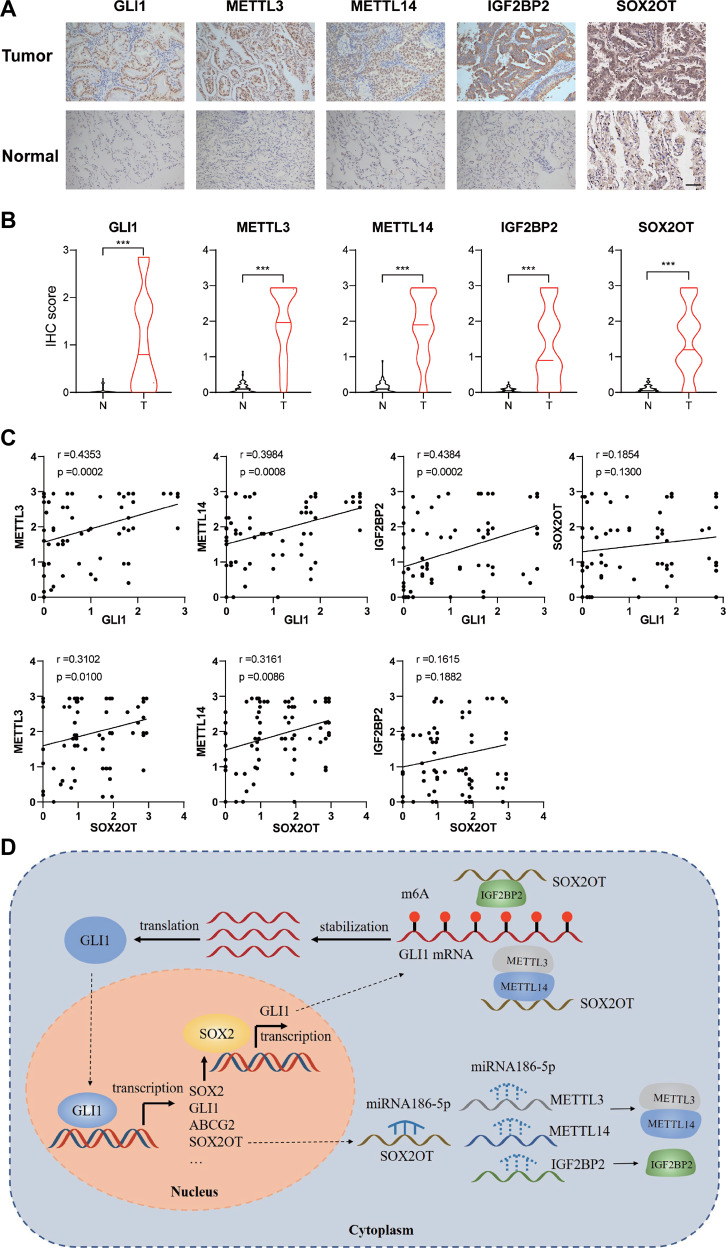
Positive correlation of the GLI1/SOX2OT loop and METTL3/14/IGF2BP2 expression at the tissue level. **A** Representative immunohistochemistry images of the GLI1/SOX2OT loop and METTL3/14/IGF2BP2. Magnification = ×200, Scale bar = 100 μm. **B** Expression level of the GLI1/SOX2OT loop and METTL3/14/IGF2BP2 in paired NSCLC specimens and adjacent normal tissues. **C** Correlation analysis between the expression levels of GLI1 with that of METTL3, METTL14, IGF2BP2, and SOX2OT in lung cancer specimens. **D** Diagram illustrating the postulated molecular mechanisms of the GLI1/SOX2OT loop m6A modification in NSCLC cells.

To test the expression of the GLI1/SOX2OT loop and the relevant m6A modifiers investigated in this study, we obtained a tissue chip comprising 75 pairs of lung cancer specimens and adjacent normal tissues. Immunohistochemistry indicated that GLI1 and METTL3/14/IGF2BP2 were significantly upregulated in lung cancer tissues compared to those in paired normal tissues (Fig. 8A, B). Additionally, in situ hybridization revealed that SOX2OT expression was higher in lung cancer specimens than that in adjacent normal tissues (Fig. 8A, B). Correlation analysis indicated that the expression level of GLI1 is positively correlated to that of IGF2BP2, METTL3/14, and SOX2OT in lung cancer bulk tumor (Fig. 8C). Our results suggested that in addition to the canonical SMO-dependent activation, GLI1 could be stimulated by SOX2OT-mediated m6A modification in a non-canonical SMO-independent way. On the one hand, SOX2OT promoted METTL3/14/IGF2BP2 transcription by acting as a ceRNA to sponge miR-186-5p and blocking its interference with METTL3/14/IGF2BP2. On the other hand, SOX2OT might guide METTL3/14/IGF2BP2 to *GLI1* mRNA to promote its m6A modification and protect it from degradation. Accumulated GLI1 activated SOX2/SOX2OT transcription by interacting with their promoter, ensuring positive self-regulation and consistent activation of the GLI1-SOX2OT loop in lung cancer cells (Fig. 8C).

## Discussion

In this study, expression level and phenotypic analyses (including proliferation and sphere formation assays) revealed that GLI1 and SOX2OT formed a positive loop to regulate the stemness of lung cancer. Mechanistic analysis using ChIP and luciferase reporter assay robustly proved that GLI1 stimulated SOX2OT expression upon binding to 300 bp upstream of the TSS of SOX2OT. SOX2OT positively regulates SOX2 transcription by sponging miR-200c and endorses stemness in bladder cancer and pancreatic ductal adenocarcinoma cells [26, 27]. SOX2 is a downstream target of GLI1, and the GLI1/SOX2 axis contributes to stemness-related EGFR resistance in lung cancer cells [27]. In melanoma cells, SOX2 activates GLI1 in a non-canonical SMO-independent way by binding to the CTTGGATT sequence at -423 bp upstream of the TSS and activating GLI1 expression [28]. In line with this, we found that SOX2 also promoted GLI1 transcription by directly binding to its promoter in NSCLC cells.

Hyperactivation of the SHH cascade in basal cell carcinoma and medulloblastoma is mainly due to repressive mutations in PTCH or activating mutations in SMO. We and other groups have previously found that the key transcription factor of the SHH pathway, GLI1, is upregulated and serves as a therapeutic target in lung cancer specimens; however, genetic alterations were rarely detected [6, 7, 9]. Patients with SHH pathway overactivation are primarily insensitive or develop secondary drug resistance to SMO inhibitors owing to new mutations in SMO or compensatory activation of GLI1 in a non-canonical way. Here, we unveiled a novel SMO-independent non-mutational manner of GLI1 activation via SOX2OT-facilitated and METTL3/14/IGF2BP2-mediated m6A modification. Mechanistic disruption of the GLI1/SOX2OT loop provided experimental evidence of how the positive loop was formed and exerted stemness upon m6A modification.

Recent studies have reported that the three m6A modifiers investigated in this study promote the propagation of various cancer cells, including lung cancer cells. METTL3 is highly expressed in distinct tumor types and boosts stemness by enhancing *SOX2* mRNA stability upon m6A modification [29–31]. METTL3 positively regulates prostate cancer cell proliferation and metastasis through GLI1 rather than other components of the SHH cascade in an m6A modification-dependent manner, while the underlying mechanism remains to be investigated further [32]. IGF2BP1 interacts with and stabilizes *GLI1* mRNA from decay in hepatocellular carcinoma [33]. Our data revealed that the GLI1/SOX2OT RNA loop is functionally mediated by m6A modifiers in lung cancer cells. SOX2OT facilitated METTL3/14/IGF2BP2-mediated m6A modification of *GLI1* mRNA and rescued them from decay. In contrast, GLI1 promoted the expression of METTL3/14/IGF2BP2 through the SOX2OT/miR-186-5p axis, forming a positive feedback loop. We unveiled a novel GLI1/SOX2OT positive loop activated by METTL3/14/IGF2BP2-modified m6A methylation to reinforce the stemness of lung cancer cells.

The existence of cancer stemness is the main cause of drug resistance and distal metastasis- and CSC-targeted therapies hold promise in the clinical treatment of cancers [34, 35]. The SHH pathway and its key transcription factor GLI1 play an essential role in cancer stemness-mediated tumorigenesis and multiple drug resistance in various cancers. Combined inhibition of the JAK2-STAT3 and Hedgehog pathways attenuates stemness and metastasis of breast cancer [36]. GLI1 inhibition facilitates multiple therapeutic approaches in distinctive cancer types [37–39]. GLI1 is also essential for stemness-related malignancy in lung cancer. In vitro experiments indicated that GLI1 activation leads to resistance of NSCLC cells to erlotinib treatment [40]. Natural products targeting GLI1 exert potent anti-angiogenesis capability in lung cancer [41]. Arsenic trioxide retards the cancer stem-like transition of small-cell lung cancer cells and regulates stem cell marker expression both in vitro and in vivo through GLI1 repression [42]. GLI1 expression level predicts poor outcomes in patients diagnosed with squamous cell carcinoma [43]. Our data unveiled that GLI1 repression reversed METTL3/14/IGF2BP2 overexpression-induced proliferative advantage of lung cancer stem-like cells, suggesting its pivotal role in the whole network. Based on the postulated mechanisms, GLI1 inhibition may lead to the downregulation of its target genes, including stemness-related SOX2 and ABCG2 in lung cancer cells, thus underlying its potential therapeutic interference. Pharmacological intervention of GLI1 to block signaling transduction may reverse the stemness-associated malignant features of lung cancer cells.

Our data demonstrate that compared with parental cells, paired lung cancer specimens and spheroids exhibit GLI1 overexpression, promoting stem-like cell proliferation. Further, the SHH cascade and its executor GLI1 are aberrantly overexpressed in both CSCs and DDP-resistant lung cancer cells. SOX2 promotes GLI1 transcription by directly interacting with its promoter; SOX2OT stabilizes the *GLI1* mRNA via METTL3/14/IGF2BP2-mediated m6A modification. In contrast, GLI1 promotes SOX2OT transcription by stimulating its promoter activity, thereby forming a GLI1-SOX2OT positive loop. SOX2OT has been demonstrated to promote GLI1 expression via histone H3 methylation and acetylation, leading to multiple drug resistance in lung cancer [19]. We believe that SOX2OT acting as ceRNA to protect mRNA of METTL3/14/IGF2BP2 and guiding METTL3/14/IGF2BP2 to GLI1 mRNA is the pivotal mode of action of SOX2OT in GLI1 regulation since SOX2OT is located mainly in the cytoplasm of lung cancer cells (>85% in A549 and >70% in H1299 cells).

The data disclose a new GLI1 stimulation pathway independent of the SHH/PTCH/SMO cascade and offer new insights into the stemness-related malignancy of lung cancer cells. Pharmacological interventions of the loop remarkably retard lung cancer tumor growth by repressing the expression of relative molecules in vivo. The present study provides insights into improving CSC-targeted therapy in cancer. Combination therapy of chemo drugs or targeting drugs with inhibitors or nucleic acid drugs targeting the GLI1-SOX2OT loop in vivo may further help in identifying novel therapeutic sensitizers for multiple regimens of cancer treatment.

## Methods

### Cell culture and treatment

Human lung cancer cell lines H1299 and H358 and the parental A549 and cisplatin (DDP)- and 5-fluorouracil (5-FU)-resistant cells were used in the study. Details regarding their cultivation are provided in the Supplementary Methods.

### RNA immunoprecipitation (RIP) assay

RIP assay was performed with Magna RIP™ RNA-Binding Protein Immunoprecipitation Kit (Millipore, Massachusetts, Massachusetts, USA) according to the manufacturer’s instructions. Briefly, cells (1 × 10^8^) were collected and washed with pre-cold PBS and lysed in polysome lysis buffer. The cell lysates were then incubated with RIP buffer containing magnetic beads that were conjugated with the indicated antibody or IgG as the negative control. Then, the mixtures were digested with proteinase K to purify the immunoprecipitated RNA. Finally, the isolated RNA was subjected to quantitative reverse transcription (RT-qPCR) to amplify the binding targets.

### Chromatin immunoprecipitation (ChIP)

ChIP was performed with Magna ChIP® HiSens Chromatin Immunoprecipitation Kit (Millipore) as described previously [44]. Cells (2 × 10^8^) were fixed with 37% formaldehyde, and the reaction was quenched using 2.5 M glycine. Cells were washed with ice-cold PBS and collected via centrifugation. Cell pellets were lysed using a lysis buffer containing protease inhibitors, and chromatin DNA was sheared into 100–500 base pair (bp) fragments using nucleic acid lyase. Anti-Gli1 (Santa Cruz Biotechnology, Beijing, China, 1:100) and anti-SOX2 (Zen-BioScience, Chengdu, China, 1:100) antibodies were used for immunoprecipitation. The chromatin-antibody complexes were precipitated with magnetic beads and washed with lysis buffer and 1× Tris buffer saline. Crosslinked sections were incubated overnight at 65 °C to reverse the cross-linking. Finally, DNA was extracted using Dzup Genomic DNA Isolation Reagent (Sangon Biotech, Shanghai, China) and analyzed using PCR. The primers used are listed in Supplementary Table S1.

### RT-qPCR

Total RNA was extracted and purified from cultured cells using an RNA extraction kit (Promega, Madison, Wisconsin, USA) and reverse-transcribed using the RevertAid First Strand cDNA Synthesis Kit (Thermo Fisher Scientific, Waltham, USA). RT-qPCR was performed using 2×EasyTaq ® PCR SuperMix (TransGen Biotech, Beijing, China). Glyceraldehyde-3-phosphate dehydrogenase (*GAPDH*) was amplified in parallel as a control. Three parallel reactions were performed using the Bio-Rad CFX96 Touch Real-time PCR Detection System (Bio-Rad). The relative expression level was calculated using the 2^−^^ΔΔCt^ method as described previously [23]. The primers used are listed in Supplementary Table S2.

### Western blot

Cells were collected and lysed with Radioimmunoprecipitation assay buffer containing protease inhibitor cocktails on ice for 30 min. The total protein concentration was measured by a BCA protein Assay kit (Sangon Biotech). Protein in whole cell lysate (30 µg/sample) was separated on SDS-PAGE gels and transferred to PVDF membranes using electroblotting. The membranes were blocked with 5% milk at 25 °C for 1 h. Membranes were then incubated at 4 °C for 12 h with primary antibodies, including anti-GLI1 (Santa Cruz Biotechnology,1:300), anti-SOX2 (Zen-BioScience, Chengdu, China, 1:500), anti-SMO (Proteintech, Chicago, Illinois, USA, 1:1000), anti-ERCC1 (BOSTER Biological Technology, Wuhan, China, 1:800), anti-METTL3(BOSTER Biological Technology, 1:800), anti-METTL14 (BOSTER Biological Technology, 1:1000), anti-IGF2BP2 (Proteintech, 1:1000), anti-m6A (Abcam, Cambridgeshire, England, 1:800), anti-RPL22 (Proteintech, 1:1000), β-actin (BOSTER Biological Technology, 1:2500), and α-Tubulin (BOSTER Biological Technology, 1:2500). Subsequently, membranes were incubated in HRP-conjugated secondary antibodies (goat anti-rabbit or goat anti-mouse, BOSTER Biological Technology, 1:2000) at room temperature for 1 h. Finally, the immunoreaction was visualized using ECL HRP substrates and detected using Bio-rad ChemiDoc XRS. The band intensity was quantified using ImageJ software (National Institutes of Health, Bethesda, Maryland, USA).

### Luciferase reporter assay

Candidate sequences were inserted into the pGMGLI-Lu plasmid (Genomeditech, Shanghai, China) using a ClonExpress^®^ II One Step Cloning Kit (Vazyme, Nanjing, China). Wild-type or mutant luciferase reporter vector generated using Mut Express II Fast Mutagenesis Kit V2 (Vazyme) were transfected into 293T cells with Lipo3000. After 48 h, luciferase activity was measured according to the instructions provided with the Firefly & Renilla Dual Luciferase Assay Kit (UElandy, Suzhou, China).

### RNA and protein stability assays

To measure RNA stability, 5 μg/mL actinomycin D (Sigma, St. Louis, Missouri, USA) was added to cells, and total RNA was extracted using an RNA extraction kit (Promega) after 0, 6, and 12 h, followed by RT-qPCR analysis. For protein stability analysis, 10 μg/mL cycloheximide was added to cells, and total protein was extracted (as previously described) after 0, 2, 4, 6, and 8 h, followed by western blot analysis.

### RNA in situ hybridization

A tissue microarray (HlugA150CS03) was obtained, and probe hybridization was performed by Shanghai Outdo Biotech Co., Ltd. (Shanghai, China) to detect the transcription levels of lncRNA SOX2OT in 75 paired lung cancer and adjacent specimens. Probes detecting SOX2OT were synthesized and used according to the instructions of the Enhanced Sensitive ISH Detection Kit I (MK11012-h, BOSTER Biological Technology). Results were screened and scored according to the signal intensity. The whole visual field of tissues was observed under a low-power microscope. The staining was classified into weak positive, medium positive, and strong positive signals, which appeared light yellow (1+), brownish yellow (2+), and brown (3+), respectively.

### Xenograft mouse model establishment and drug administration

Female BALB/C nude mice (Ji’nan Pengyue Laboratory Animal Breeding Co., Ltd., Ji’nan, China) aged 6–8 weeks were used in animal experiments. To construct the subcutaneous xenograft mouse model, each mouse was subcutaneously injected with 200 μL PBS containing 5 × 10^6^ A549 cells. Two weeks after the injection, saline or drugs were injected intraperitoneally. Specifically, 100 μL/d physiological saline was injected into mice in the control group. Mice in three treatment groups (*n* = 6 each group) were injected with either 50 mg/kg/d GANT58, 10 mg/kg/d SAH, or 50 mg/kg/d GANT58 + 10 mg/kg/d SAH every 2 days, and tumor dimensions and mouse weight were measured every 2 days via digital caliper measurements until day 21. The tumor volume was calculated using the formula V = (length × width^2^)/2. All mice were sacrificed humanely after day 23, and tumors were collected for subsequent experiments.

### Immunohistochemistry staining (IHC)

Tumor tissues were fixed with 4% paraformaldehyde for 24 h and then embedded in paraffin. Paraffin-embedded slices (4 μm) were dried at 62 °C for 60 min and dewaxed twice in fresh xylene for 30 min, followed by hydration using gradient alcohol. After antigen retrieval in citrate buffer at 100 °C for 10 min, the slices were blocked in 5% bovine serum albumin for 1 h and incubated at 4 °C for 12 h in primary antibodies, including anti-GLI1 (Santa Cruz Biotechnology, 1:200), anti-METTL3 (BOSTER Biological Technology, 1:500), anti-METTL14 (BOSTER Biological Technology, 1:500), and anti-IGF2BP2 (Proteintech1:500). Next, the slices were incubated with biotin-labeled secondary antibodies (BOSTER Biological Technology) for 60 min and incubated with horseradish enzyme-labeled chain avidin solution (BOSTER Biological Technology) for 30 min at room temperature. Positive staining was visualized with brown 3,3’-diaminobenzidine tetrahydrochloride (DAB, ZSGB, Beijing, China) and counterstained for 3 min using hematoxylin. Finally, the slices were dehydrated by increasing concentrations of ethanol and xylene and sealed with neutral balsam. Images were taken by a microscope (Olympus, Japan) with ×200 magnification.

### Immunofluorescence (IF) staining

Frozen sections (6 μm) of tumor tissues were fixed using acetone at −20 °C for 10 min. Cell membranes were permeated in PBS containing 0.1% Triton X-100 at room temperature for 8 min. The slices were blocked in 3% BSA/PBS at room temperature for 1 h and then incubated with the primary antibody (anti‐Ki‐67, Abclonal, Wuhan, China, 1:100) overnight at 4 °C. Subsequently, the slides were incubated with a fluorescent-labeled secondary antibody (Invitrogen, Carlsbad, California, USA, 1:2000) for 1 h at room temperature. Next, the nucleus was stained by DAPI (0.5 mg/mL) for 15 min at 37 °C. Images were photographed using a spectral laser scanning confocal microscope (Olympus, Japan) after the slices were sealed by antifade solution.

### Statistical analysis

Data are presented as the mean ± standard deviation. Prism software (version 8.0; GraphPad, San Diego, CA, USA) was used for statistical analysis. Comparisons of the two groups were established using a two-tailed Student’s *t*-test. Multiple comparisons were performed using one- or two-way analysis of variance. *P* < 0.05 was considered significant and indicated with asterisks.

## Supplementary information

Supplementary material

Original Data File

COI Form

Checklist

## Data Availability

The datasets generated and/or analyzed during the current study are available from the corresponding author upon reasonable request.
