# Supplementary material for: N6-methyladenine-mediated aberrant activation of the lncRNA SOX2OT-GLI1 loop promotes non-small-cell lung cancer stemness
Source: Cell Death Discov. 2023 May 6;9:149. doi: 10.1038/s41420-023-01442-w (PMC10164154; doi:10.1038/s41420-023-01442-w)
Supplement: Supplementary file 1 — Supplementary material [file 41420_2023_1442_MOESM1_ESM.docx]

**SUPPLEMENTARY INFORMATION**

**Table S1. Primers used for detecting GLI1 bound to the promoter of SOX2OT in ChIP**

|  | Primer | Sequence (5′-3′) |
| --- | --- | --- |
|  | P1-F | ACCTACTATTGGATTTTCCTC |
|  | P1-R | AAGCATTGGCCAAGATACTG |
|  | P2-F | CACAGGGATTTCAACAGAG |
|  | P2-R | TGAGGGTAGCAACTTCTTT |
|  | P3-F | CTCGTTTGTTGTGATAGGC |
|  | P3-R | CTGTATCCTGAAGGGTTTT |
|  | P4-F | GCCAATGTGAGATGGATTA |
|  | P4-R | CCAGAAGGACCTATGGAGA |
|  | P5-F | TGGCCCTATGACTCTATGA |
|  | P5-R | AACCATTACTACCCGAGCT |
|  | P6-F | GGGAACATAAACAGAGCAA |
|  | P6-R | AAAGATAACTGGGAGGATT |
|  | P7-F | GTAAGCCTAGGCTGAAAATA |
|  | P7-R | AAAGGATCTGAGGGAATGA |
|  | P8-F | GTGTAAAACTATTAGCTCTAGGC |
|  | P8-R | ATCCATTCTGGCTGGTTCT |
|  | P9-F | TTAGAGTTTTGCCCCTGAA |
|  | P9-R | TAAATTGCTTGGTTGGTTT |
|  | P10-F | TTAACAAAAGCAGGGATAT |
|  | P10-R | AGTCAATCTTTCCCATCTC |

**Table S2. Primers used for RT-qPCR analysis**

| Gene | Primer | Sequence (5′-3′) |
| --- | --- | --- |
| GLI1 | GLI1-F | CTATGGCGAGCCCTGCTGT |
|  | GLI1-R | TGGGTGAGGTGCGGATAA |
| SOX2 | SOX2-F | GGGAAATGGGAGGGGTGCAAAAGA |
|  | SOX2-R | TTGCGTGAGTGTGGATGGGATTGG |
| SOX2-OT | SOX2-OT-F | GTGTAAGGCGATGTGGGT |
|  | SOX2-OT-R | CAGGGTGTTGTCTTGTAGCA |
| SMO | SMO-F | GGGAGGCTACTTCCTCATCC |
|  | SMO-R | GGCAGCTGAAGGTAATGAGC |
|  |  |  |
| METTL3 | METTL3-F | GTCCATCTGTCTTGCCATCT |
|  | METTL3-R | CTTGTAGGAGACCTCGCTTT |
| METTL14 | METTL14-F | GTGGACGAGAAAGAAATAGA |
|  | METTL14-R | AAGAAGGTTAGAGGAGGATG |
| IGF2BP2 | IGF2BP2-E | GAAGGTCAGCCAGGTTTG |
|  | IGF2BP2-R | GATAGCGTCGTGGGAGTT |
| GAPDH | GAPDH -F | CTCCTCCTGTTCGACAGTCAGC |
|  | GAPDH -R | CCCAATACGACCAAATCCGTT |
| 18S | 18S-F | GTAACCCGTTGAACCCCATT |
|  | 18S-R | CCATCCAATCGGTAGTAGCG |


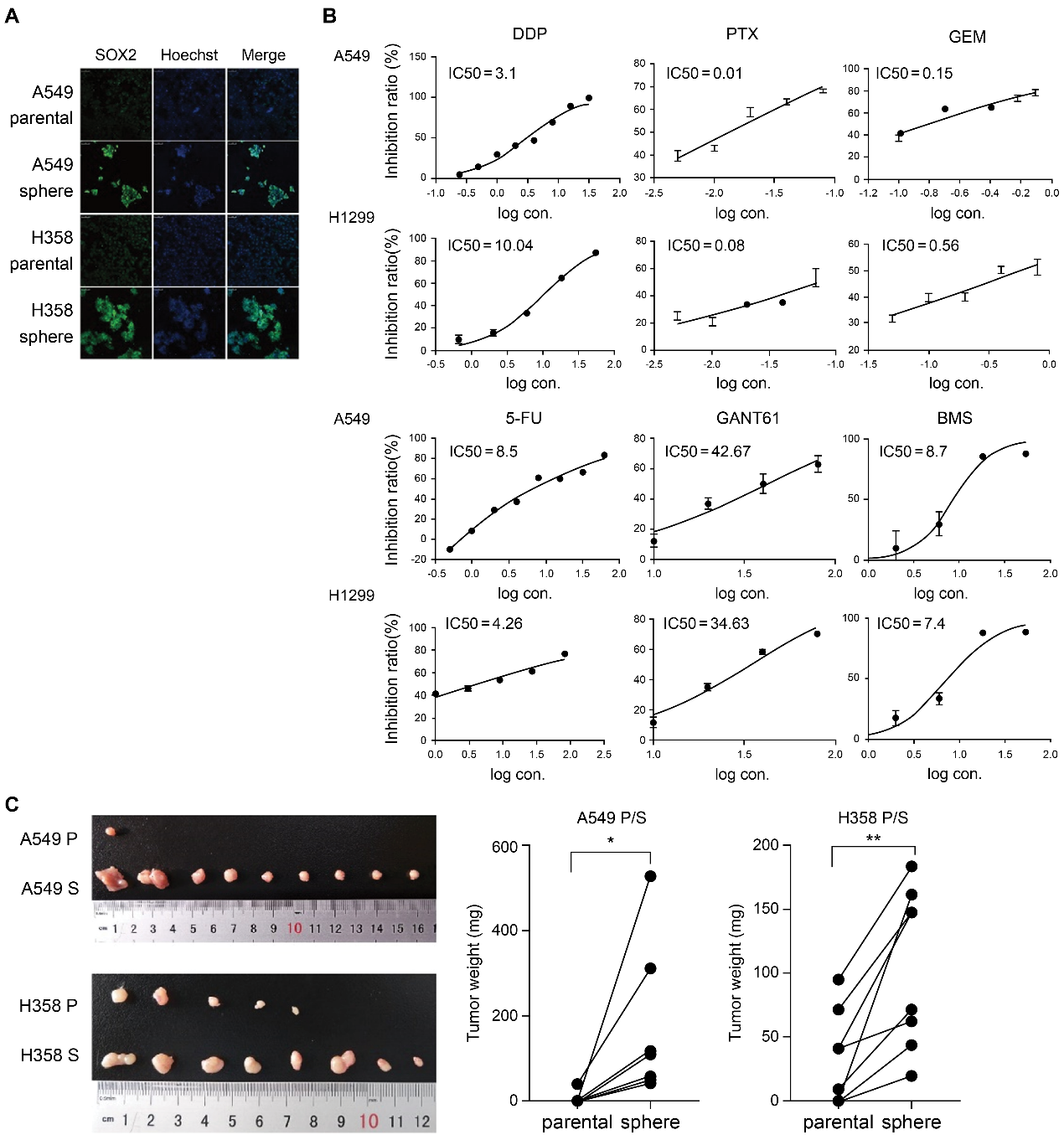


**Figure S1**. Identification of stemness features isolated by spheroid culture.


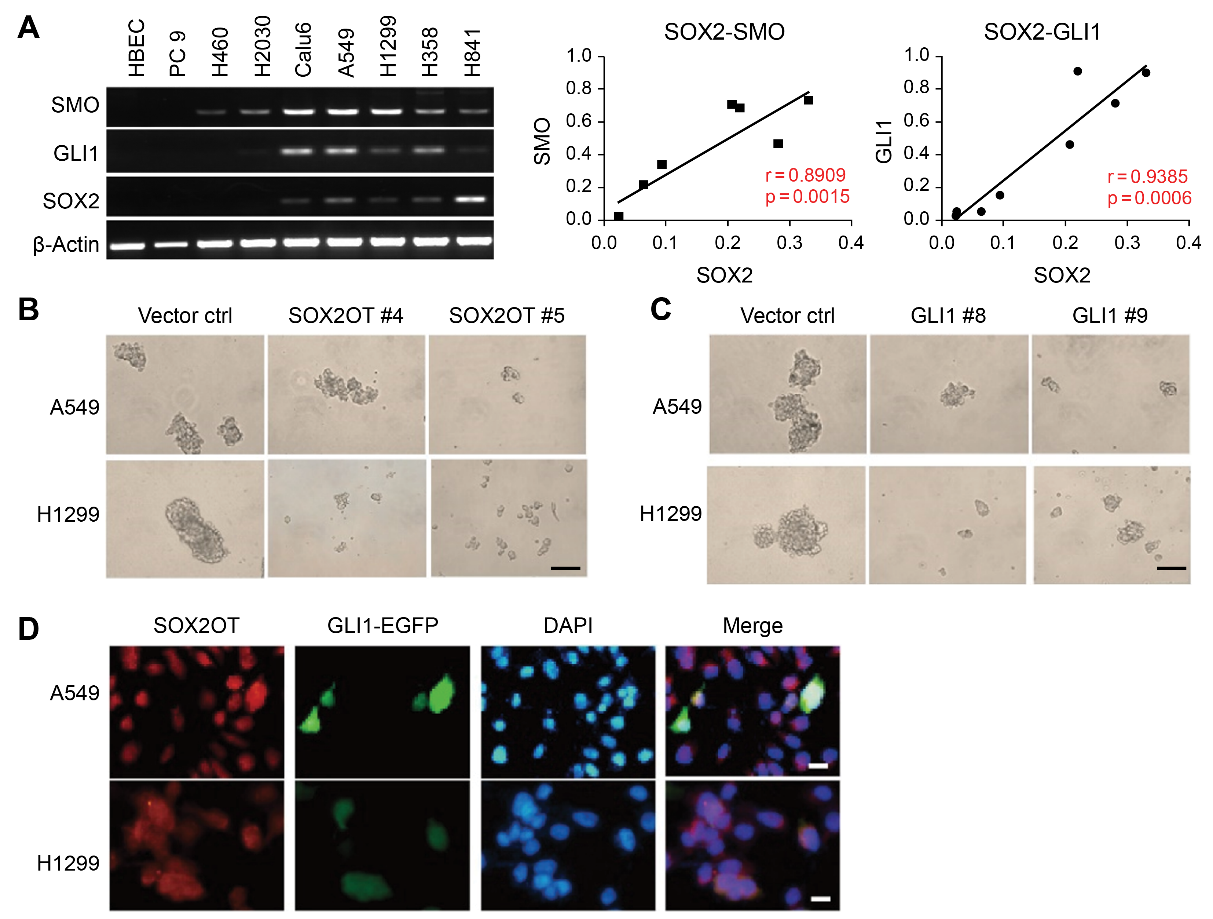


**Figure S2**. Co-expression of SOX2/SOX2OT and Hedgehog cascade.


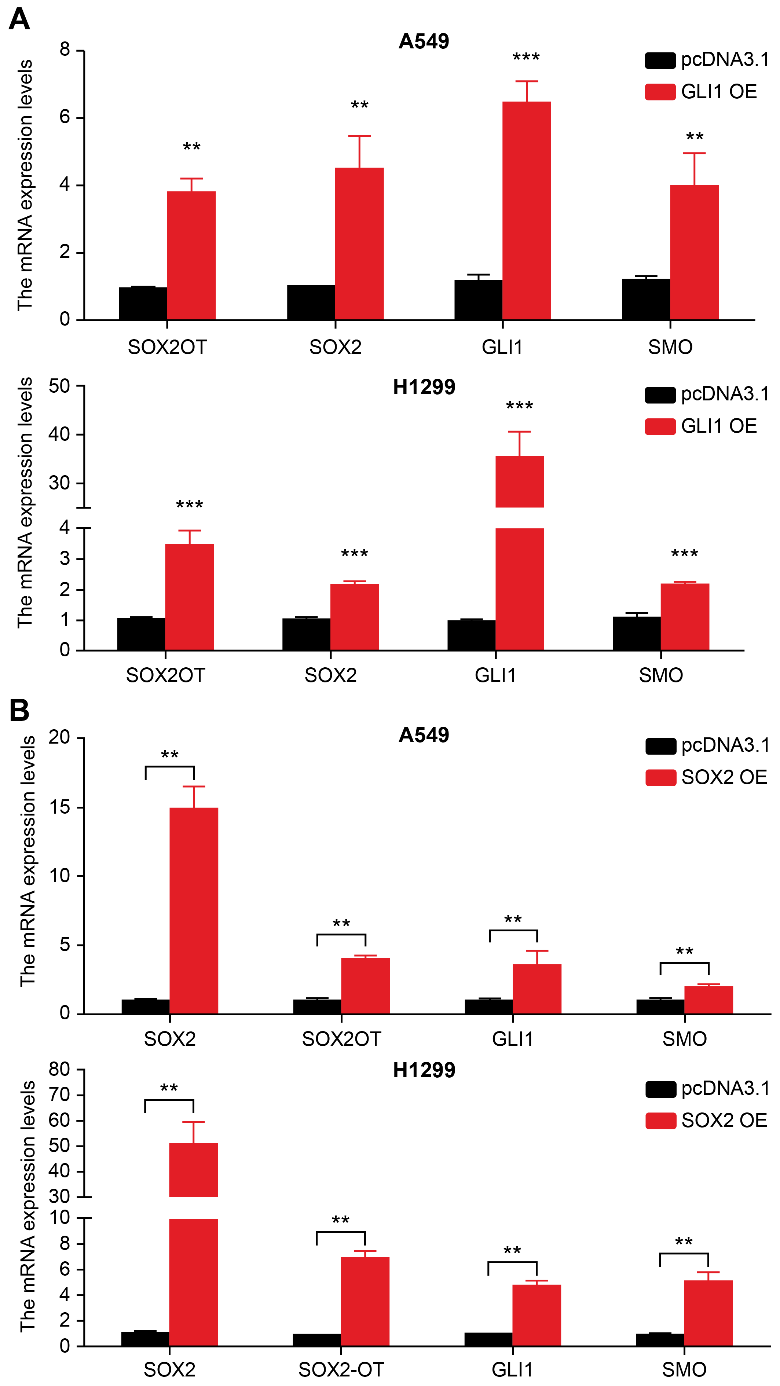


**Figure S3**. Mutual regulation of SOX2 and GLI1 in NSCLC cells.


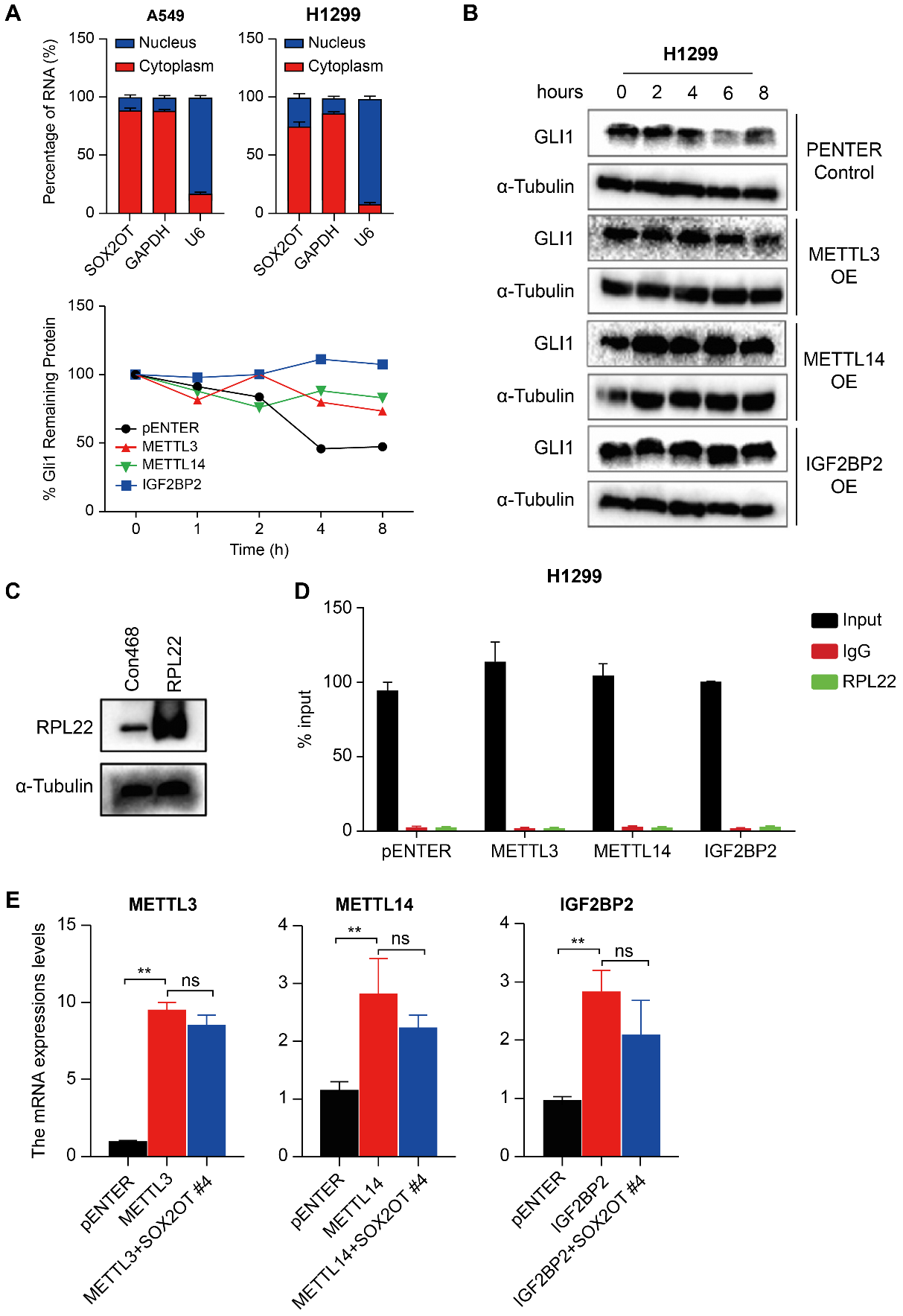


**Figure S4**. Stabilization of GLI1 protein in H1299 cells upon METTL3/14/IGF2BP2 overexpression.


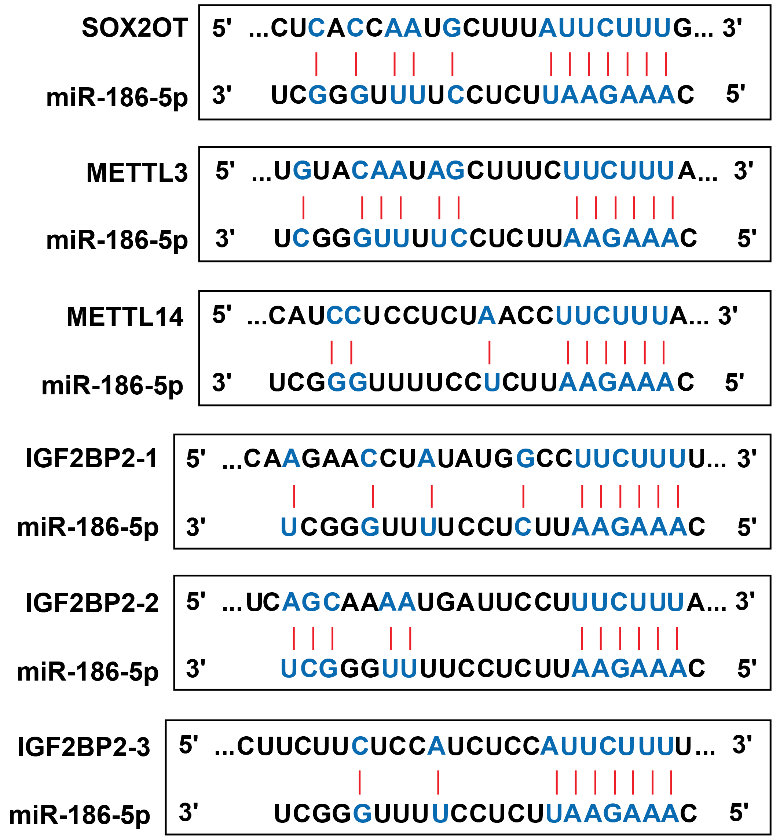


**Figure S5**. Binding sites of miR-186-5p on SOX2OT/METTL3/14/IGF2BP2 as predicted by Starbase.


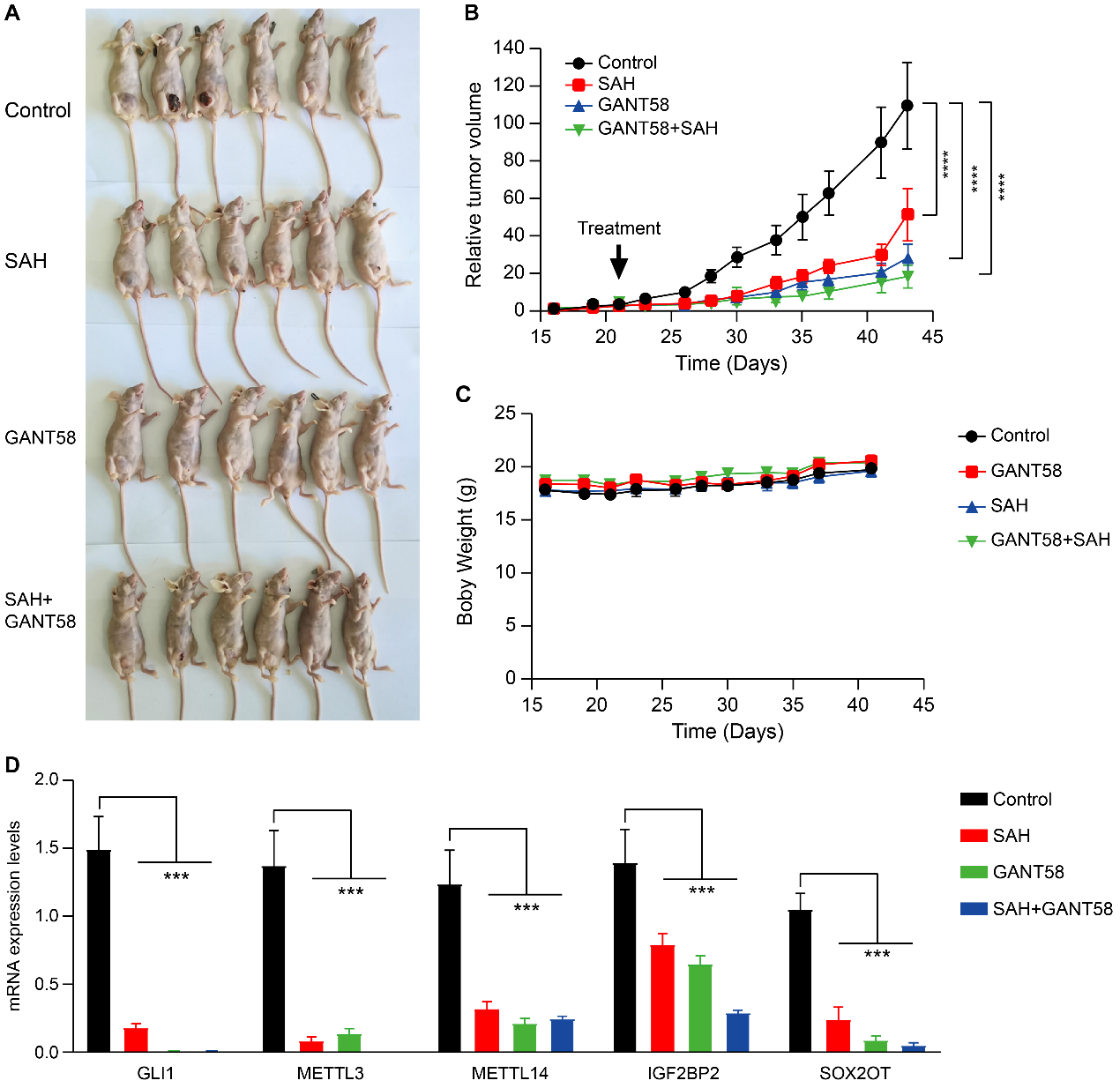


**Figure S6**. Therapeutic intervention using the GLI1-SOX2OT loop and its m6A modification in vivo.

**SUPPLEMENTAL METHODS**

**2.1 Cell culture and treatment**

The human lung cancer cell lines H1299 and H358 were purchased from the American Type Culture Collection and maintained in our laboratory. A549, H1299, and H358 cells were cultivated in Roswell Park Memorial Institute (RPMI)-1640 medium supplemented with 10% fetal bovine serum (FBS; Hyclone, USA) and penicillin/streptomycin. 293T cells were cultured in Dulbecco’s modified eagle’s medium supplemented with 10% FBS (Hyclone) and penicillin/streptomycin. All cells were incubated at 37 °C in 5% CO_2_. The parental A549 and cisplatin (DDP)- and 5-fluorouracil (5-FU)-resistant cells (A549/DDP and A549/5-FU, respectively) were purchased from the Cancer Hospital Chinese Academy of Medical Science (Peking, China) and cultivated in RPMI-1640 medium supplemented with 10% FBS and 2 μg/mL DDP or 4 μg/mL 5-FU. For individual experiments, doses of chemotherapeutic drugs were described accordingly in the figure legends.

## 2.2 Cell transfection

GLI1 and SOX2 overexpression plasmids pcDNA3.1-GLI1 and pcDNA3.1-SOX2, respectively, were cloned using the ClonExpress II One Step Cloning Kit (Vazyme, China). The short hairpin RNA plasmids GLI1#8 and GLI1#9 and the corresponding backbone GV112 were purchased from Genechem. The short hairpin RNA plasmids SOX2OT#4 and SOX2OT#5 and the empty vector pLV were purchased from Cyagen. The overexpression plasmids pENTER-METTL3, pENTER-METTL14, pENTER-IGF2BP2, and pENTER were obtained from Vigene Biosciences. The RPL22 overexpression plasmid GV657-RPL22 was purchased from Genechem. The hsa-miR-186-5p inhibitor was purchased from RiboBio. All plasmids were extracted and purified with a DNA Midiprep kit (Qiagen, Germany) and transfected into cells using Lipofectamine 3000 Reagent (Thermo Fisher Scientific, USA) following the manufacturer’s instructions. Forty-eight hours post-transfection, cells were harvested for follow-up experiments, and stable cell lines were selected with 400 μg/mL G418 or 1 μg/mL puromycin incubation.

## 2.3 Proliferation assay

Cells (2 × 10^3^) were seeded onto 96-well plates and cultured for 24 h. Cell counting kit-8 (CCK-8, Dojindo, CK04) assay was performed according to manufacturer’s instructions. A spectrophotometer (Bio-Rad, Hercules, CA, USA) was used to measure the optical density of the samples at 450 and 630 nm. For the cell proliferation analysis, CCK-8 assay was performed 24, 48, 72, and 96 h after propagation. Cells were seeded and assessed in triplicates. To calculate the half minimal inhibitory concentration (IC_50_), chemotherapy drugs were applied 24 h after cell seeding; after 48 h, the CCK8 assay was performed. IC_50_ was calculated using Graphpad software.

## 2.4 Colony formation assay

Cells (3 × 10^4^) were seeded onto six-well plates, cultured for 2 weeks, and the medium was changed appropriately. After discarding the medium, the cells were washed thrice with 1× phosphate-buffered saline (PBS), fixed with 4% formaldehyde for 15 min, and washed with 1× PBS. Finally, the cells were stained with crystal violet for 30 min, rinsed slowly under running water until the background was clean, and air dried. Images were acquired with Bio-Rad ChemiDoc XRS+, and the number of clones in each group was determined using ImageJ software (National Institutes of Health, USA).

## 2.5 Sphere formation assay

Cells (2 × 10^4^) were seeded onto 12-well plates and cultured in serum-free 1640 medium supplemented with 20 ng/mL fibroblast growth factor (Peprotech, USA), 20 ng/mL epidermal growth factor (Peprotech), and 2% B27 (Gibco, USA). Half the medium was replaced every three days. Cell spheroids were documented and quantified using an inverted microscope (Olympus, Japan) after two weeks. The diameter and number (>50 μm) of spheres were evaluated and quantified.

## 2.6 Immunoblotting

Cells were harvested and lysed with the lysis buffer on ice for 30 min. The total protein concentration was measured using BCA protein Assay Kit (Sangon Biotech, China). Whole cell proteins were separated and transferred to polyvinylidene fluoride membranes via electroblotting. The membranes were blocked with 5% milk at 25 °C for 1 h. The primary antibodies [anti-GLI1 (Santa Cruz Biotechnology, China, 1:300), anti-SOX2 (Zen-BioScience, China, 1:500), anti-SMO (Proteintech, USA, 1:1000), anti-ERCC1 (BOSTER, China, 1:800), anti-METTL3 (BOSTER, 1:800), anti-METTL14 (BOSTER, 1:1000), anti-IGF2BP2 (Proteintech, 1:1000), anti-m6A (Abcam, UK, 1:800), anti-RPL22 (Proteintech, 1:1000), anti-β-actin (BOSTER, 1:2500), and α-Tubulin (BOSTER, 1:2500)] were added and incubated with blots at 4 °C overnight, followed by horseradish peroxidase (HRP)-conjugated secondary antibody incubation. Finally, the target bands were visualized using enhanced chemiluminescence HRP substrate and detected using Bio-Rad ChemiDoc XRS. The band intensity was quantified using ImageJ.

**2.7 Immunohistochemistry**

The tissue microarray HLugA150CS03 was obtained from Shanghai Outdo Biotech CO., Ltd. to detect the expression of GLI1, METTL3, METTL14, and IGF2BP2 in 75 pairs of lung cancer and adjacent tissue specimens. The paraffin sections of tissues were antigen repaired with TE buffer at 98 °C. Tissue sections were preincubated with 10% normal goat serum, then incubated with the primary antibody (anti-GLI1, anti-METTL3, anti-METTL14, and anti-IGF2BP2) solution overnight at 4 °C. The secondary antibody was incubated with the tissue samples for 30 min at 20-25 °C, then the samples were treated with peroxidase conjugated-biotin streptavidin complex for 10 min. Lastly, samples were stained with 3,3′-diaminobenzidine and hematoxylin. Immunohistochemistry results were interpreted and scored by an experienced pathological expert according to the staining intensity and range of intracellular brown-yellow particles. For staining intensity, the score was 0 for non-yellow particles, 1 for light yellow ones, 2 for brownish yellow ones, and 3 for dark brown ones. The results were presented as immunohistochemistry (IHC) score which is percentage of positive cells multiplied by staining intensity.
