# Supplementary material for: N6-methyladenine-mediated aberrant activation of the lncRNA SOX2OT-GLI1 loop promotes non-small-cell lung cancer stemness
Source: Cell Death Discov. 2023 May 6;9:149. doi: 10.1038/s41420-023-01442-w (PMC10164154; doi:10.1038/s41420-023-01442-w)
Supplement: Supplementary file 2 — Original Data File [file 41420_2023_1442_MOESM2_ESM.docx]

FIG1-C


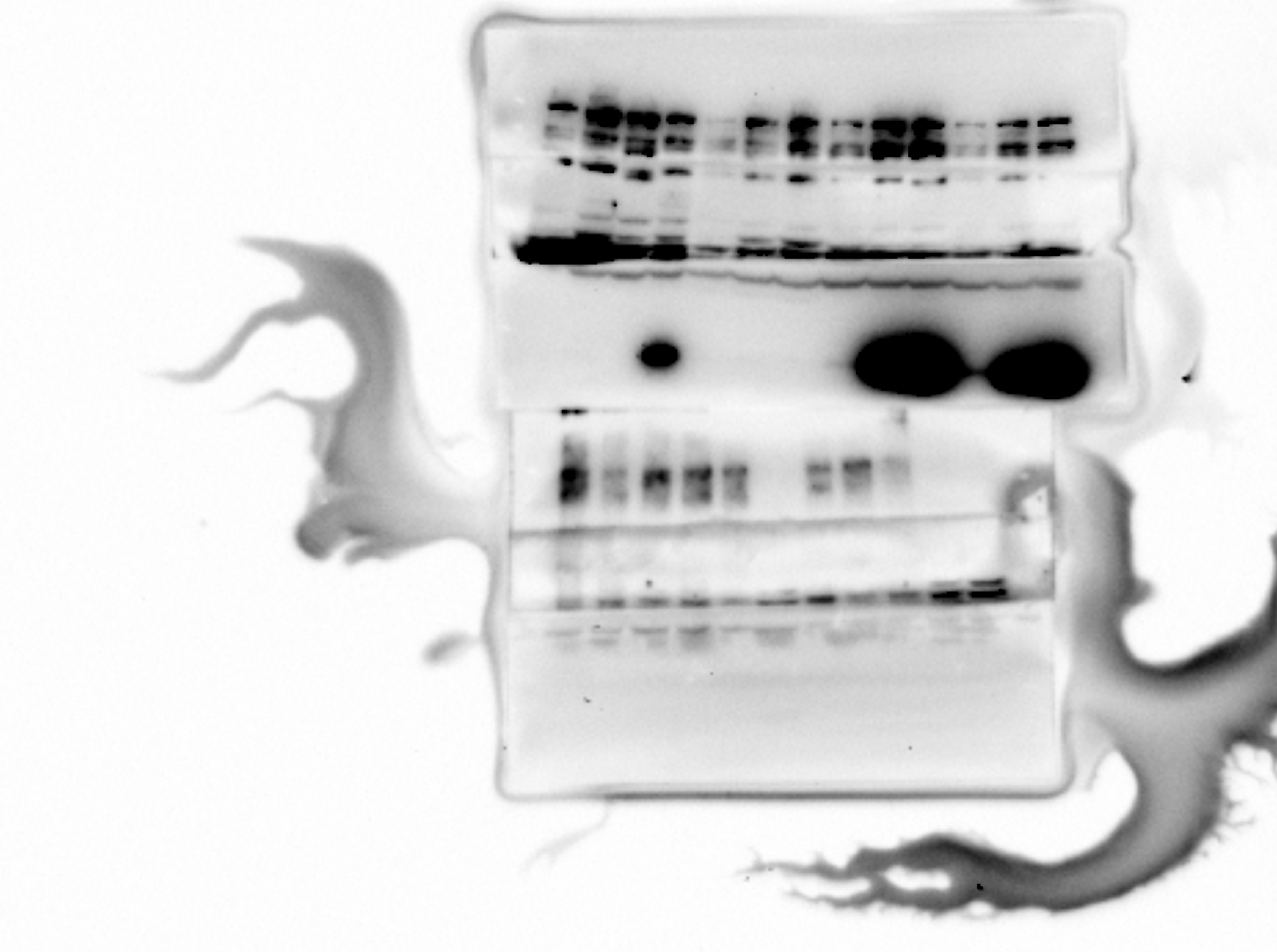


parental

Sphere1

Sphere2

parental

Sphere1

Sphere2

GLI1

SMO


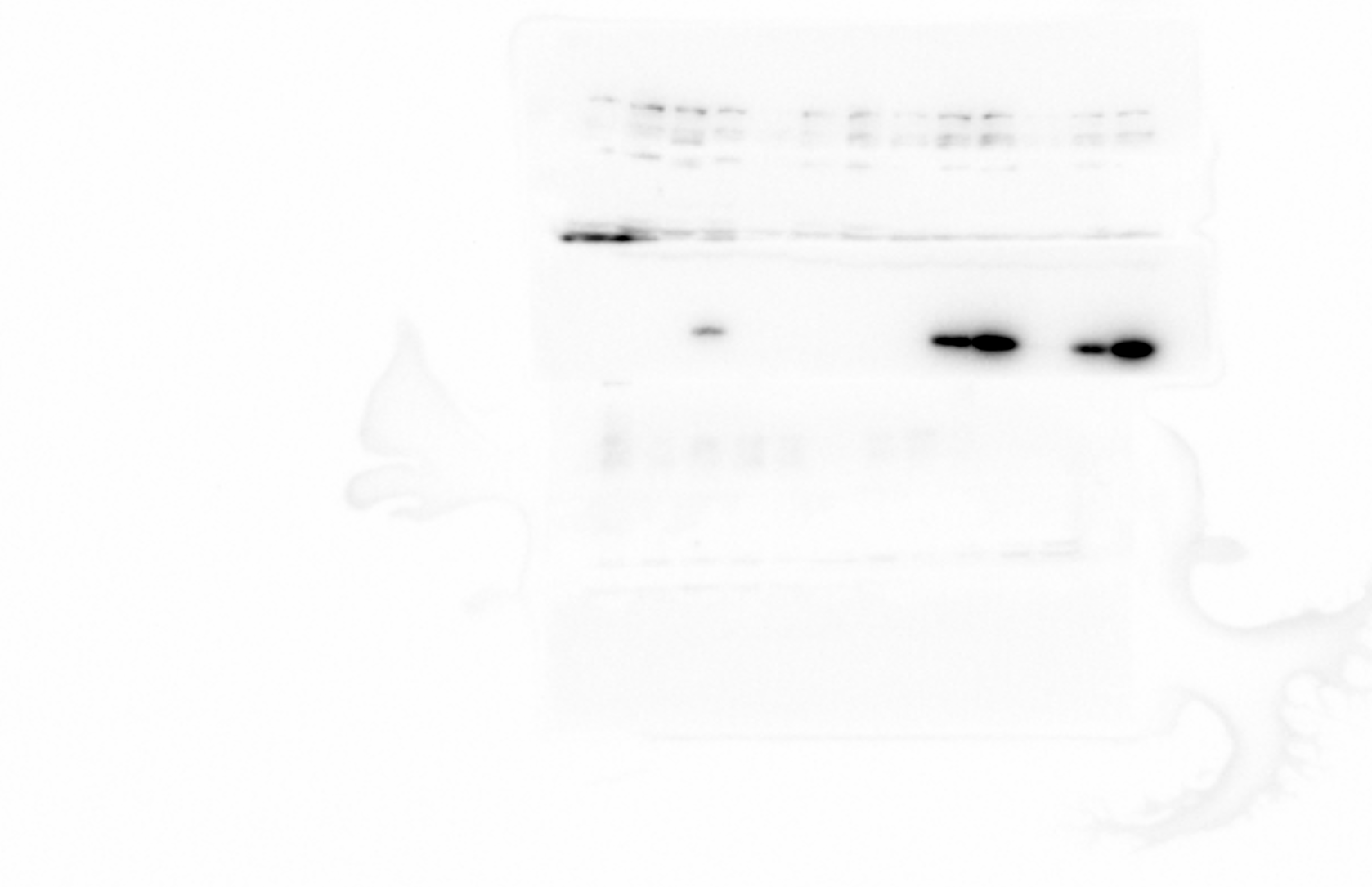

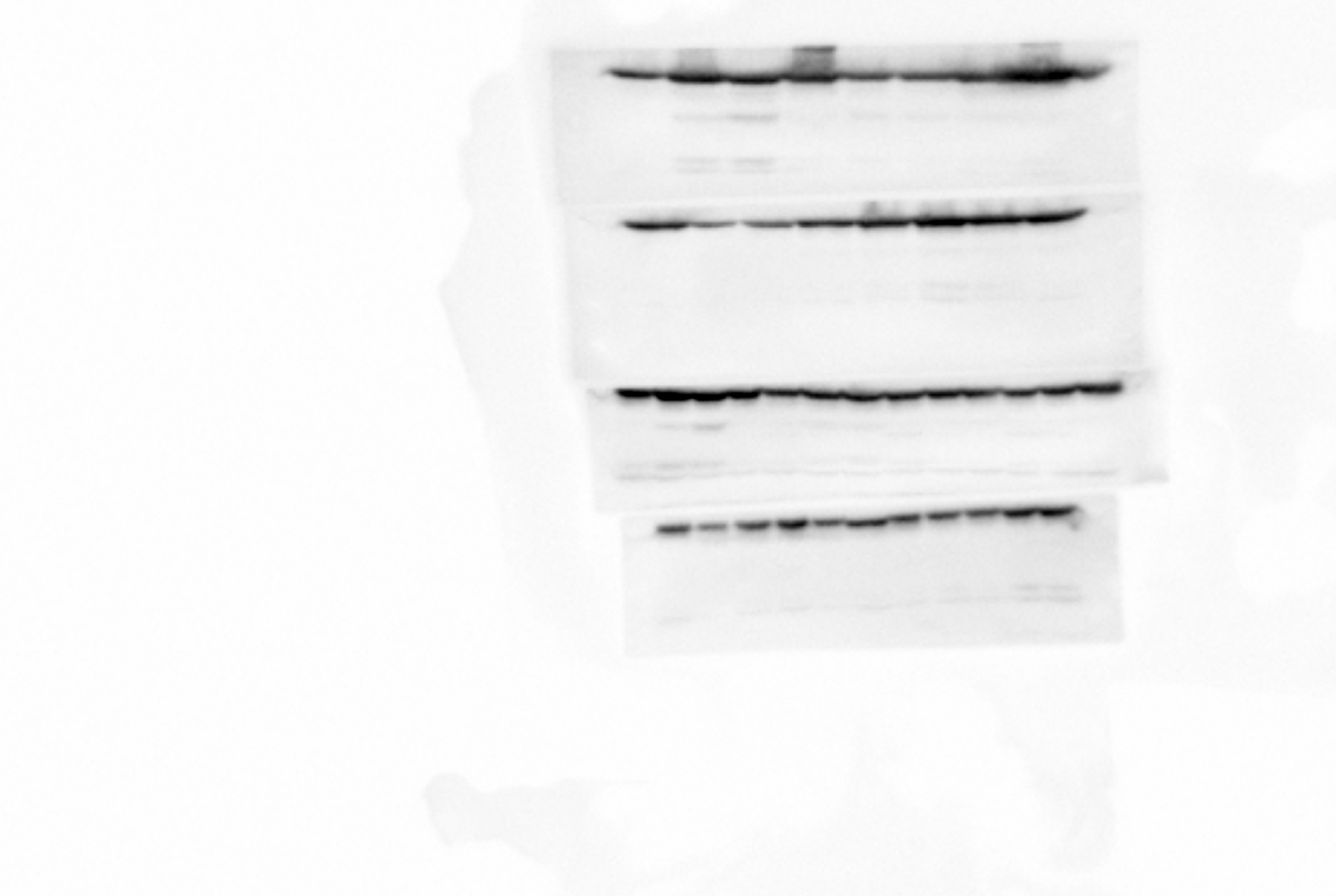


parental

Sphere1

Sphere2

parental

Sphere1

Sphere2

parental

Sphere1

Sphere2

parental

Sphere1

Sphere2

SOX2

Tubulin

FIG1-F
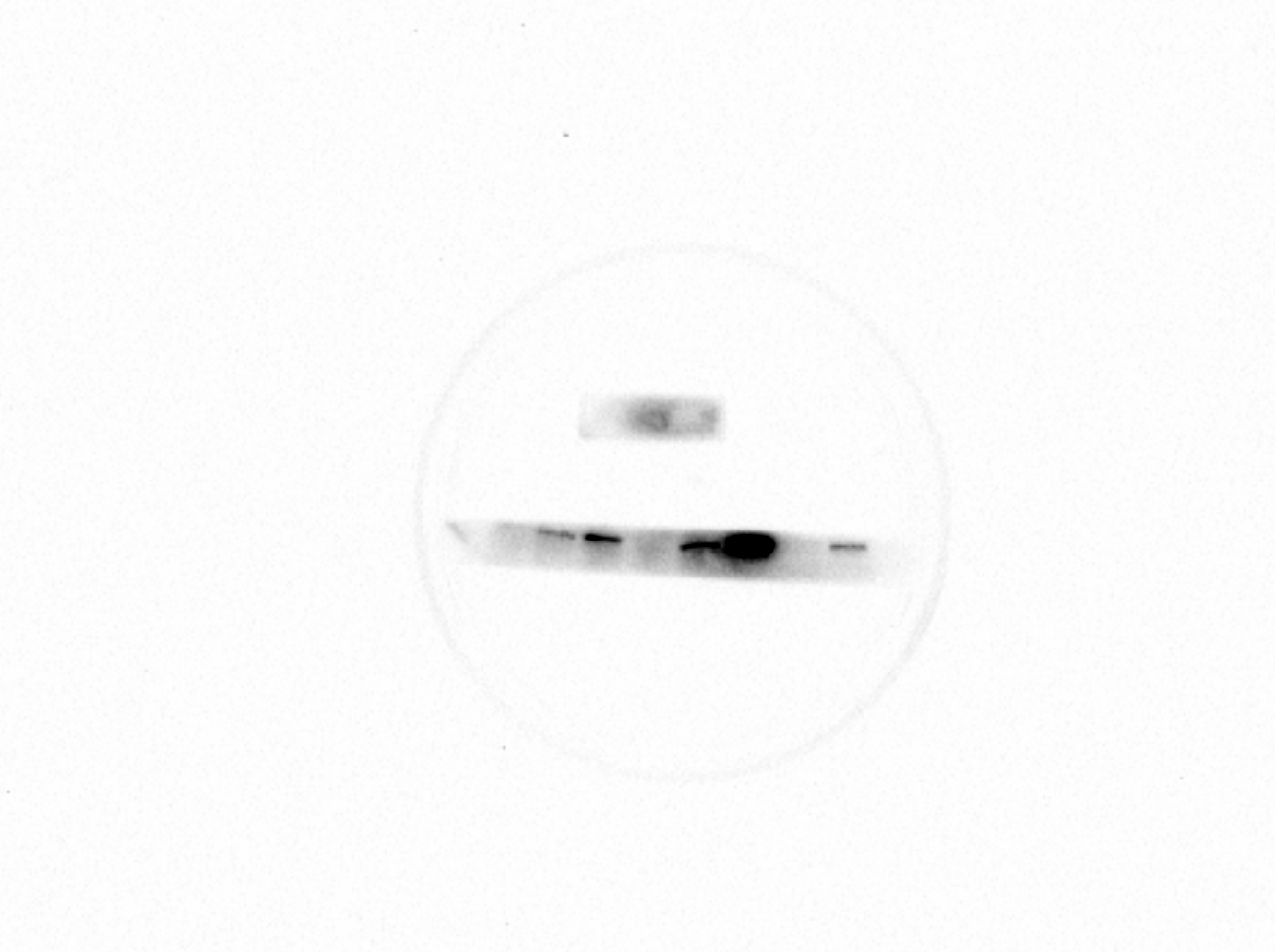


A549

A549 5FU

A549

A549 DDP

SMO


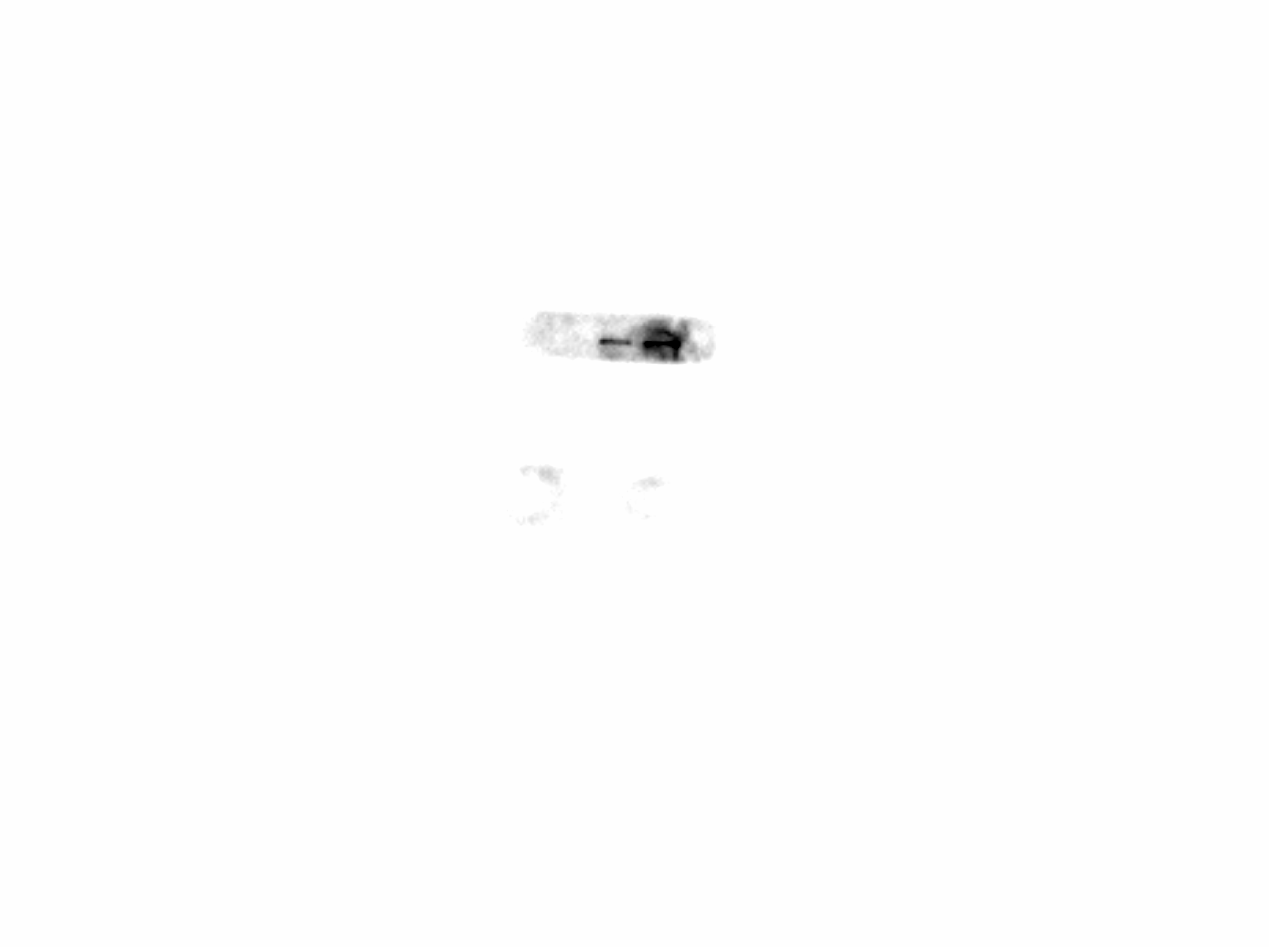


A549

A549 DDP

GLI1


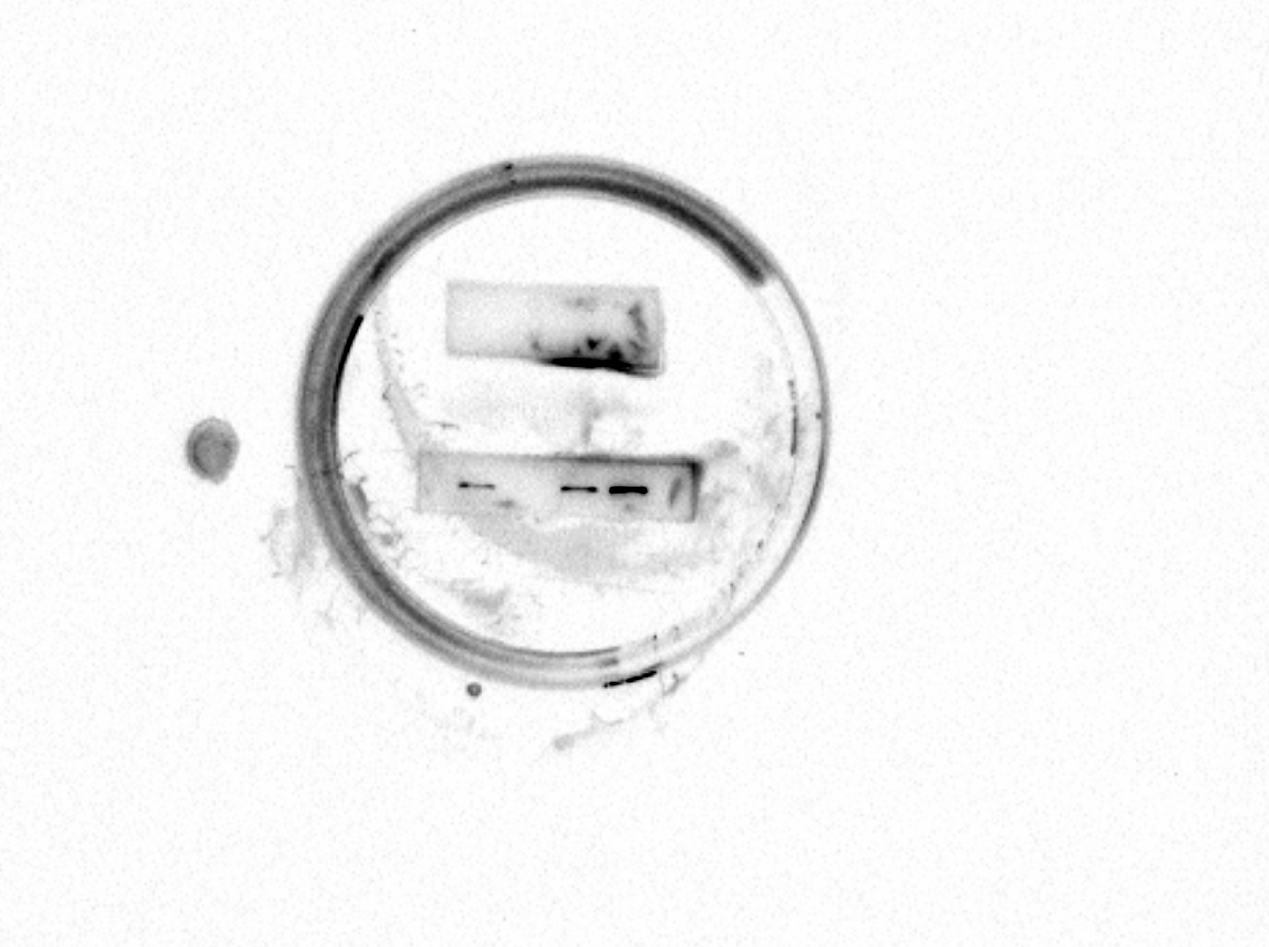


A549

A549 5FU

GLI1


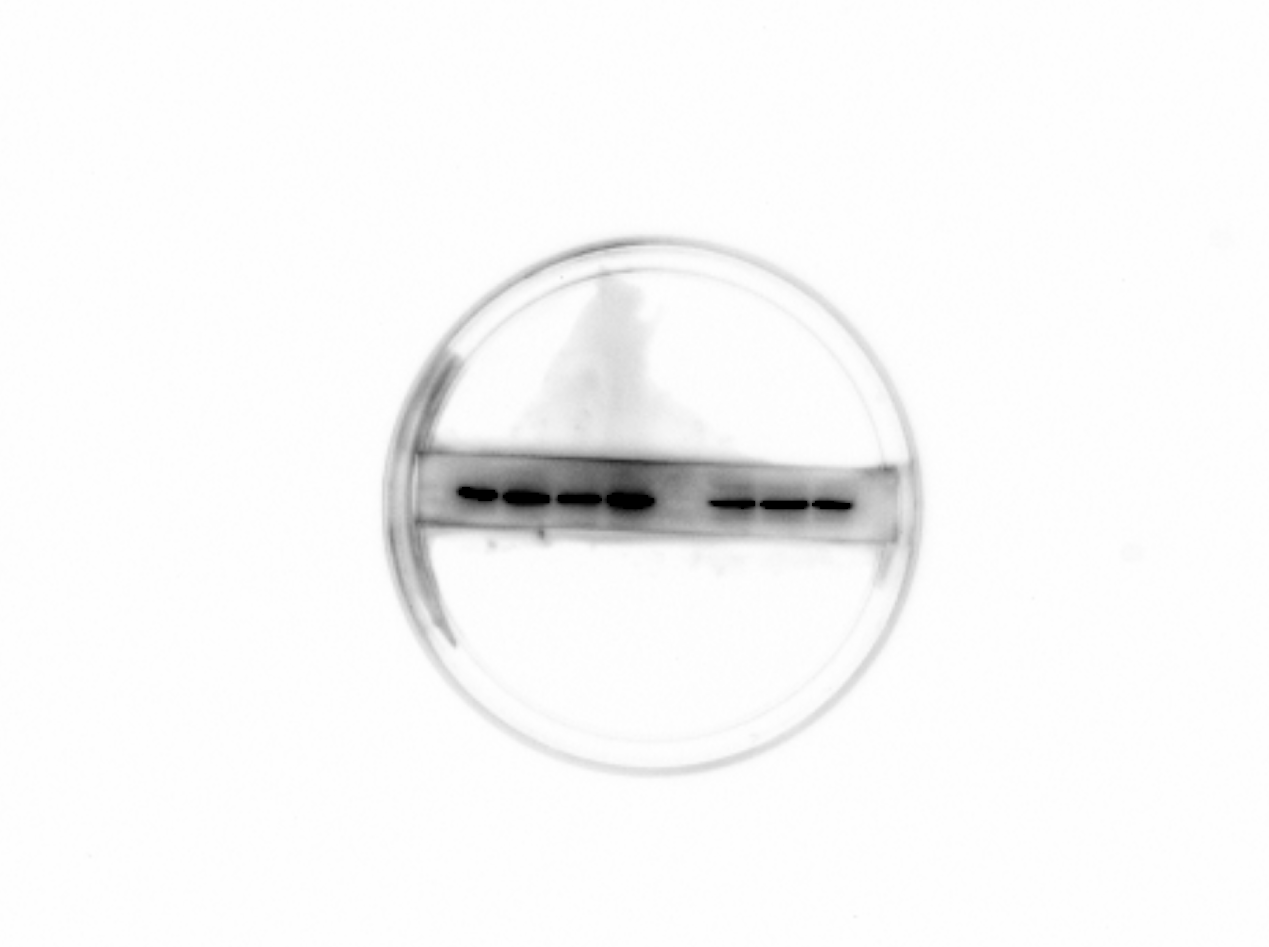


A549

A549 5FU

A549

A549 DDP

SOX2


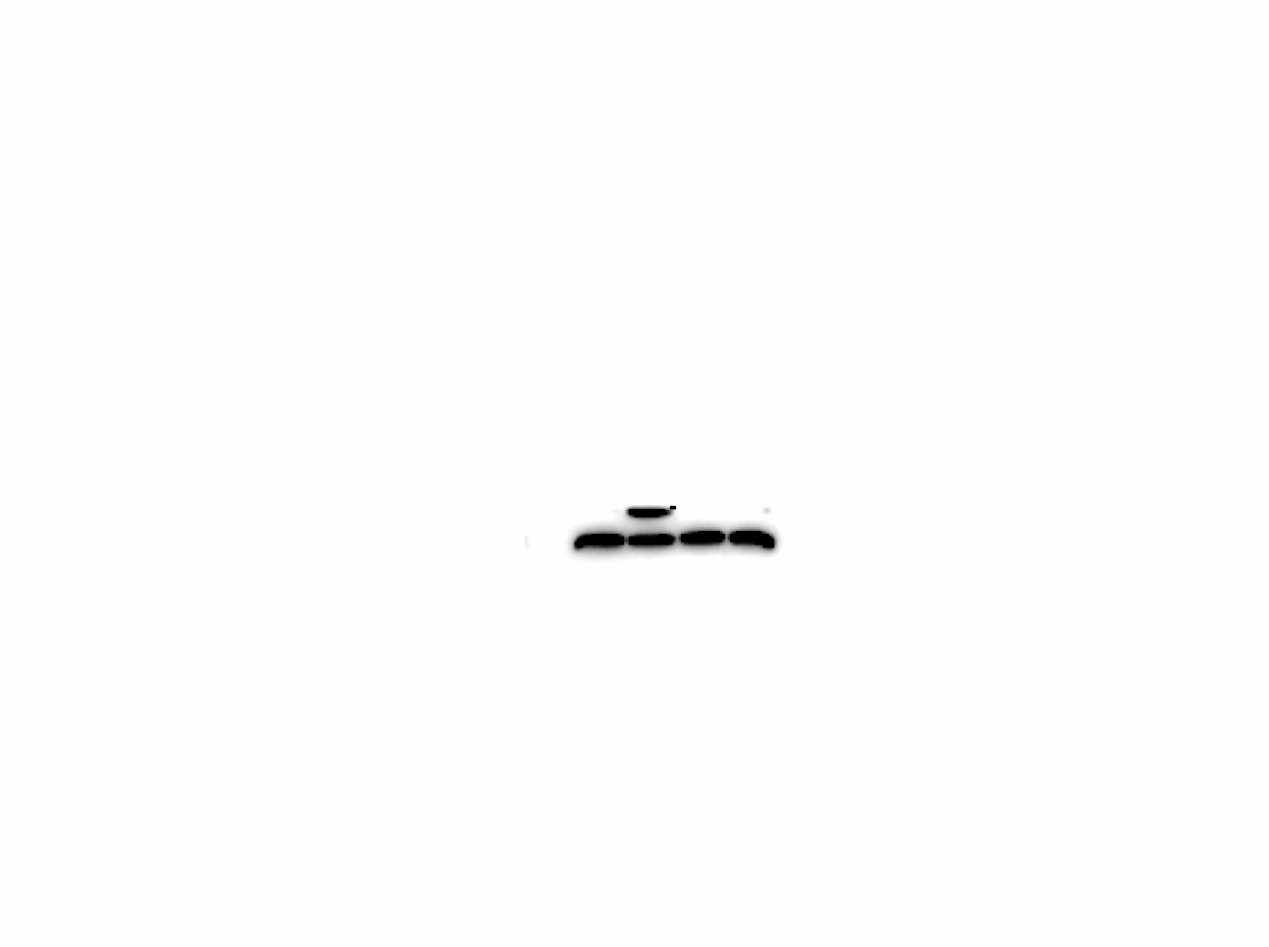


A549

A549 DDP

α-Tublin


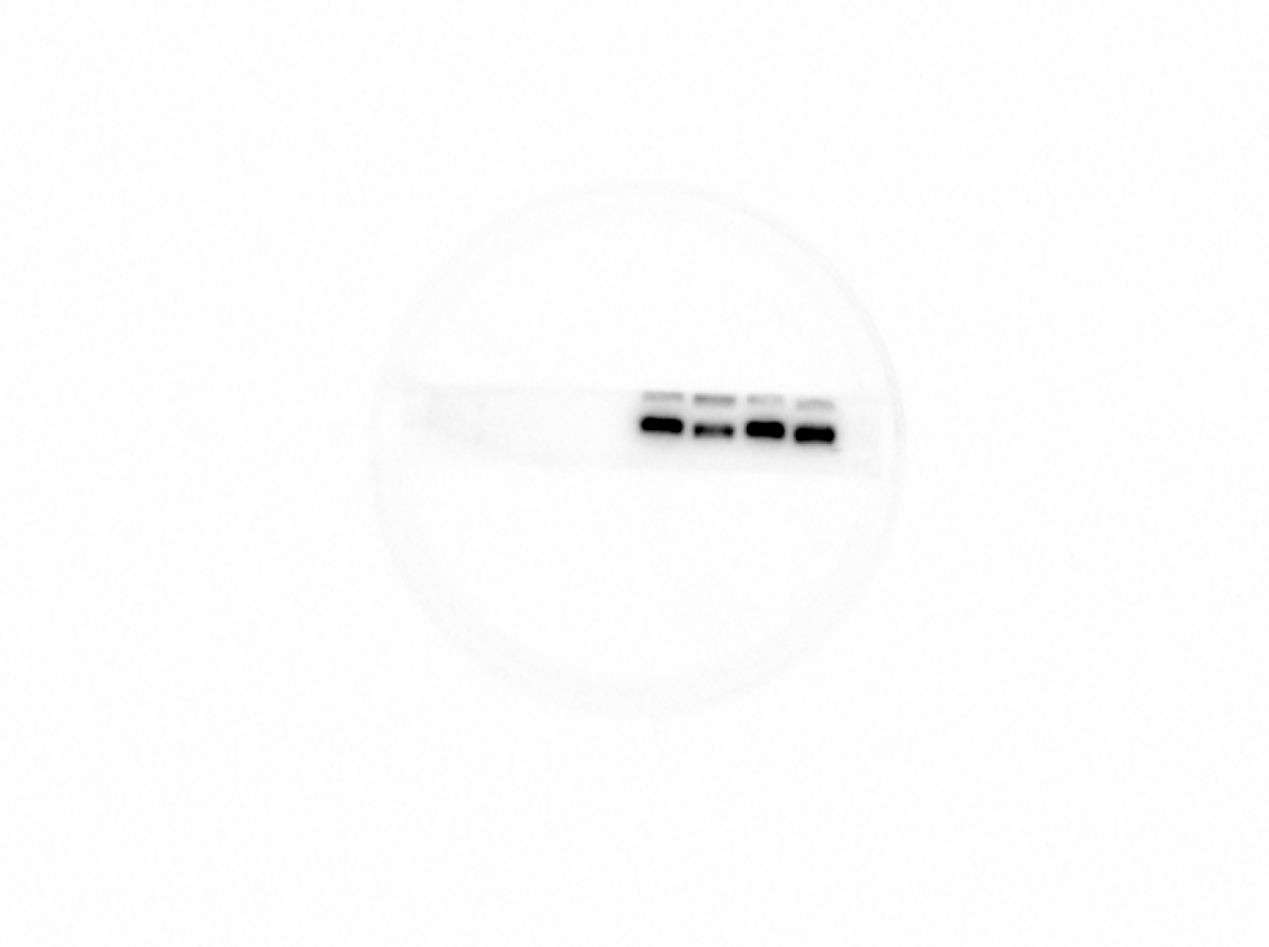


A549

A549 5FU

α-Tublin

FIG-2B
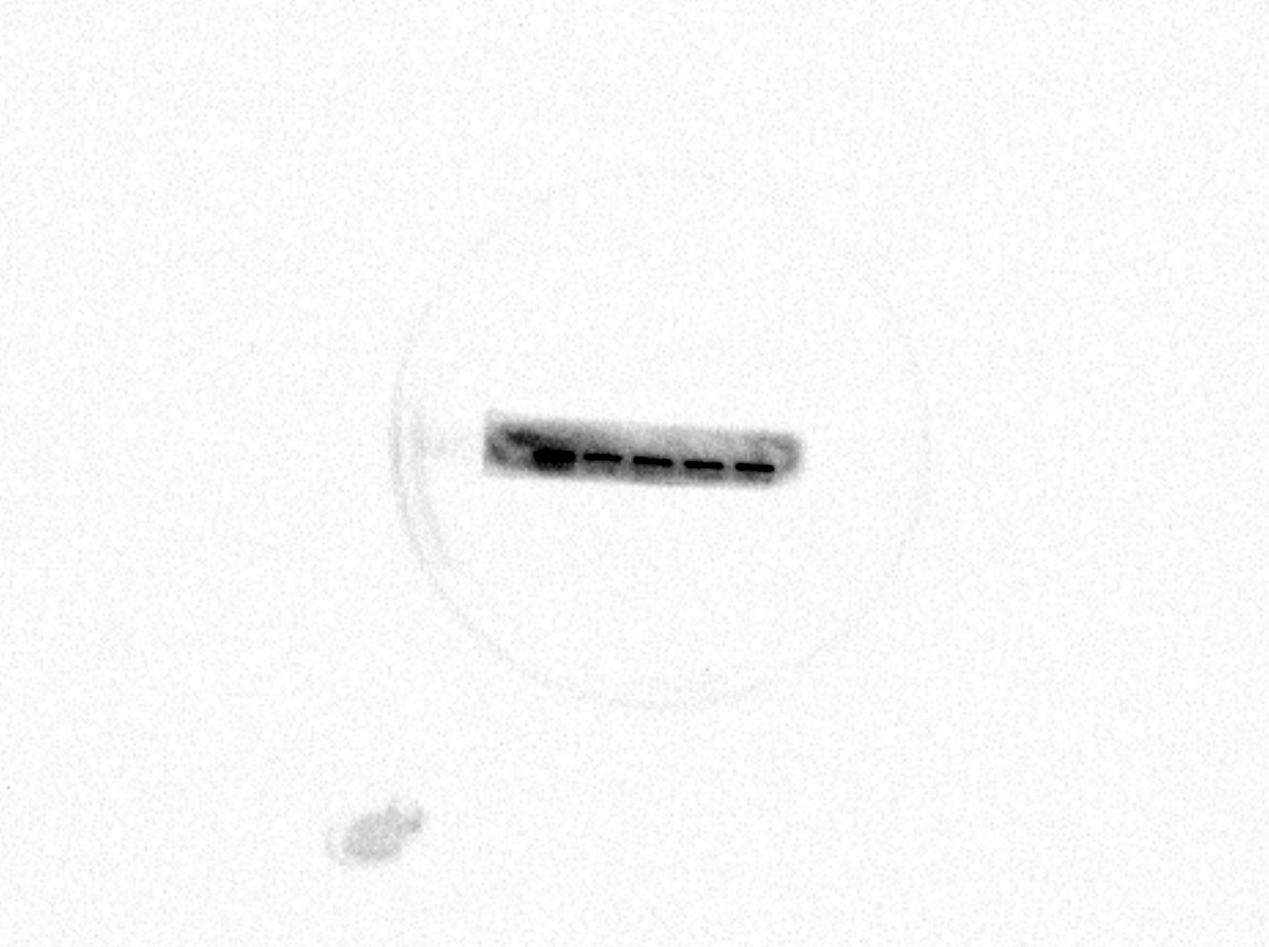


SHV

SOX2-OT #4

SOX2-OT #5

GLI1-A549


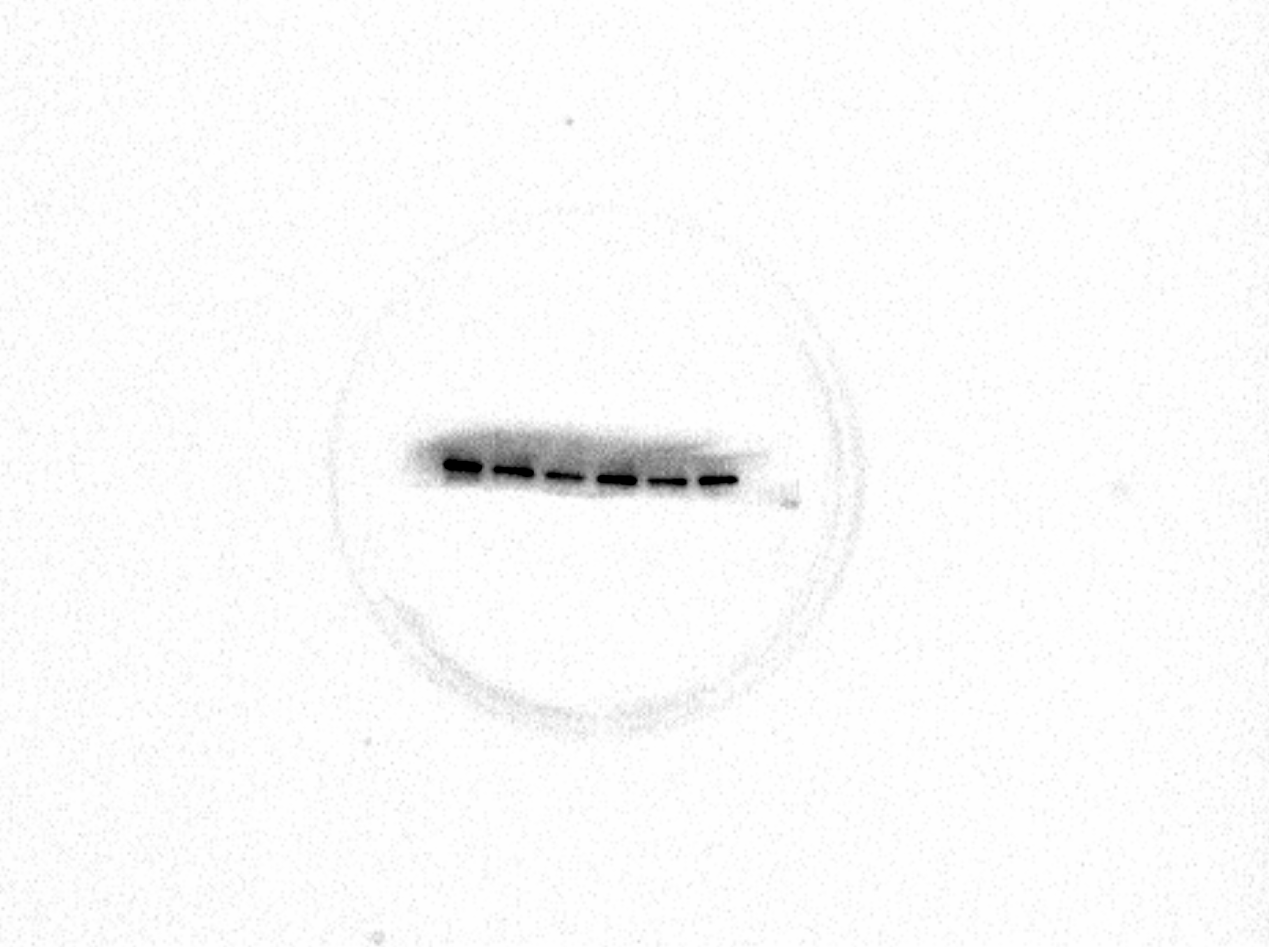


SHV

GLI1 #8

GLI1 #9

GLI1-A549





SOX2-A549

SHV

SOX2-OT #4

SOX2-OT #5


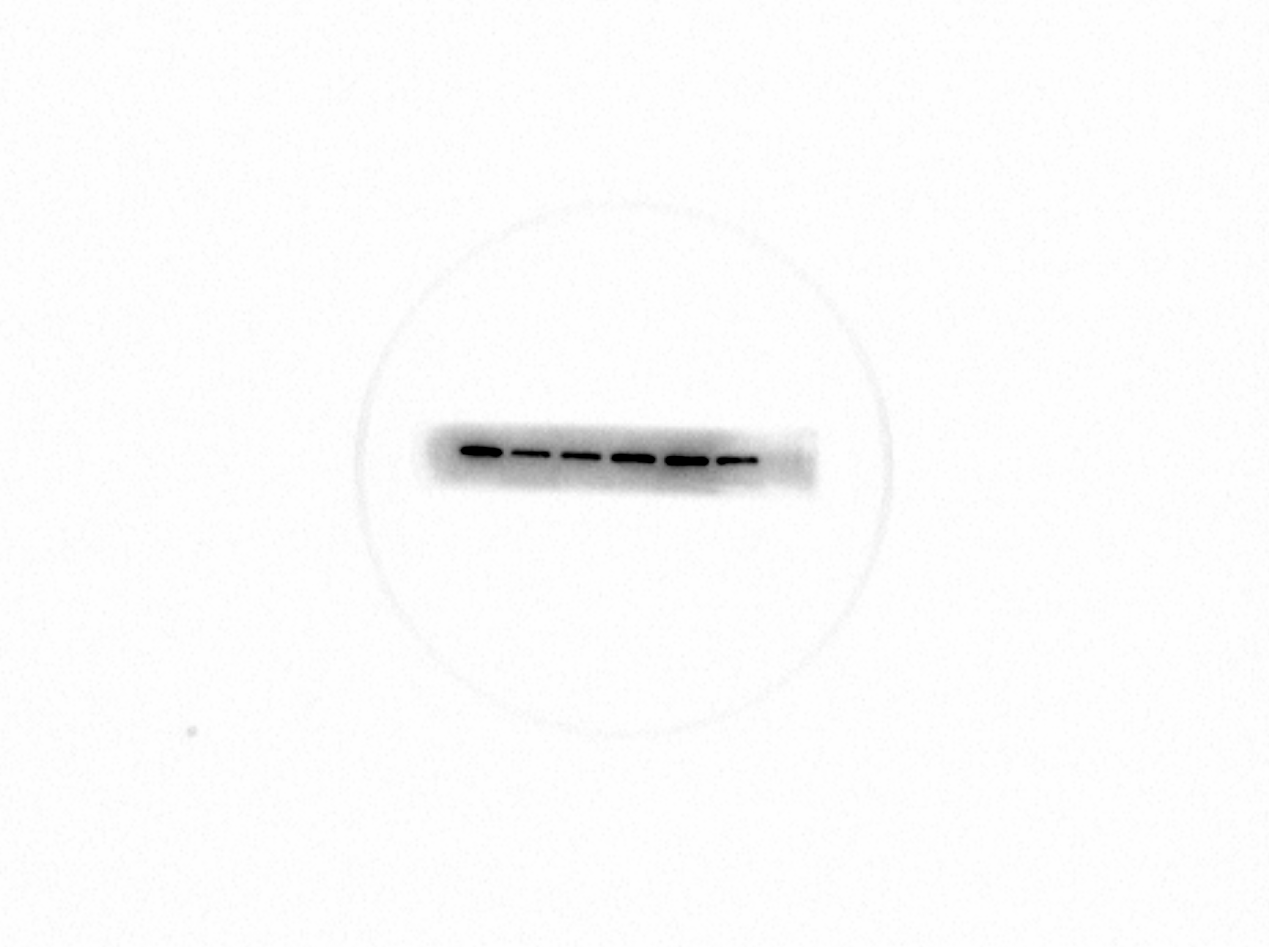


SOX2-A549

SHV

GLI1 #8

GLI1 #9


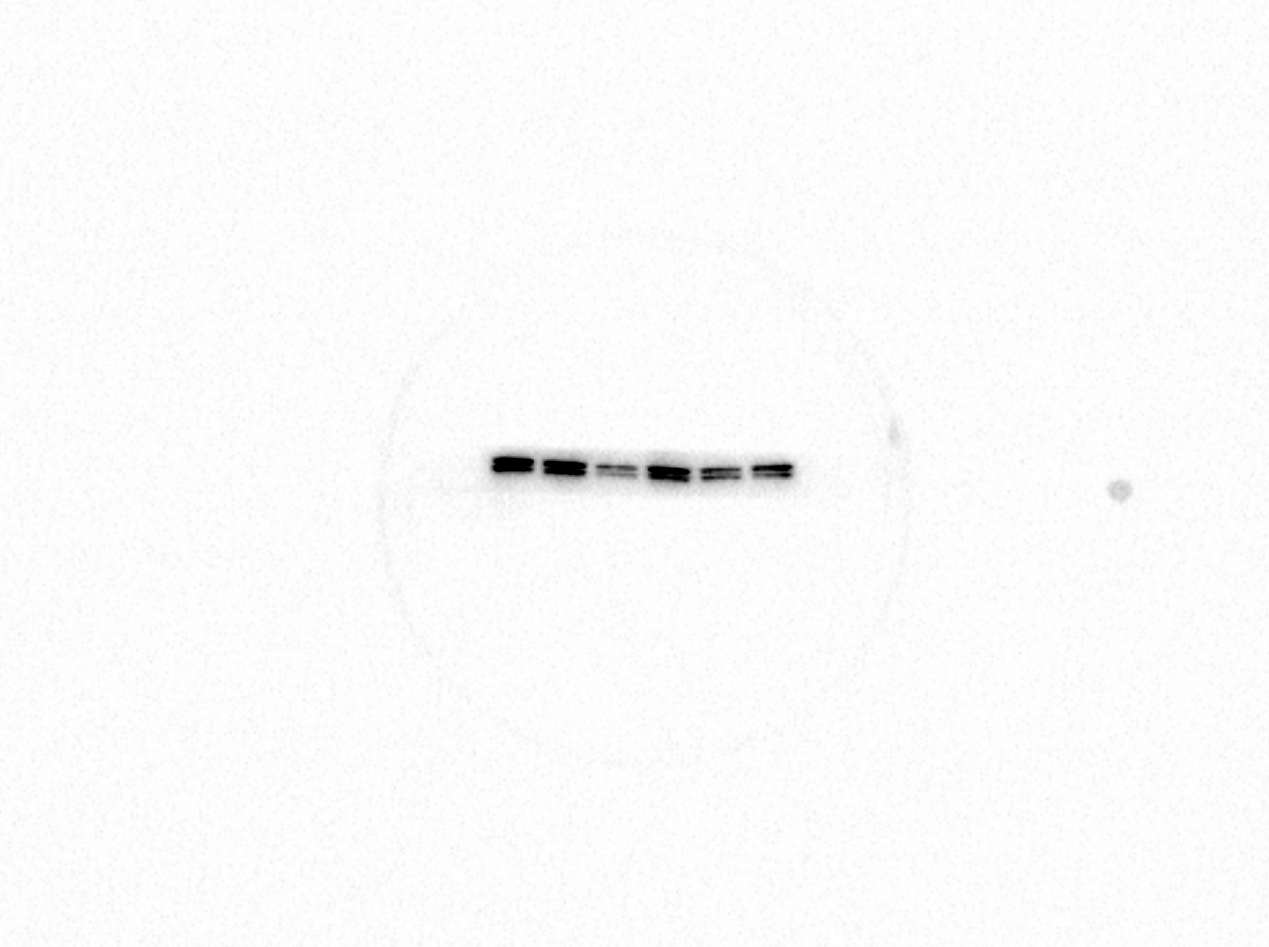

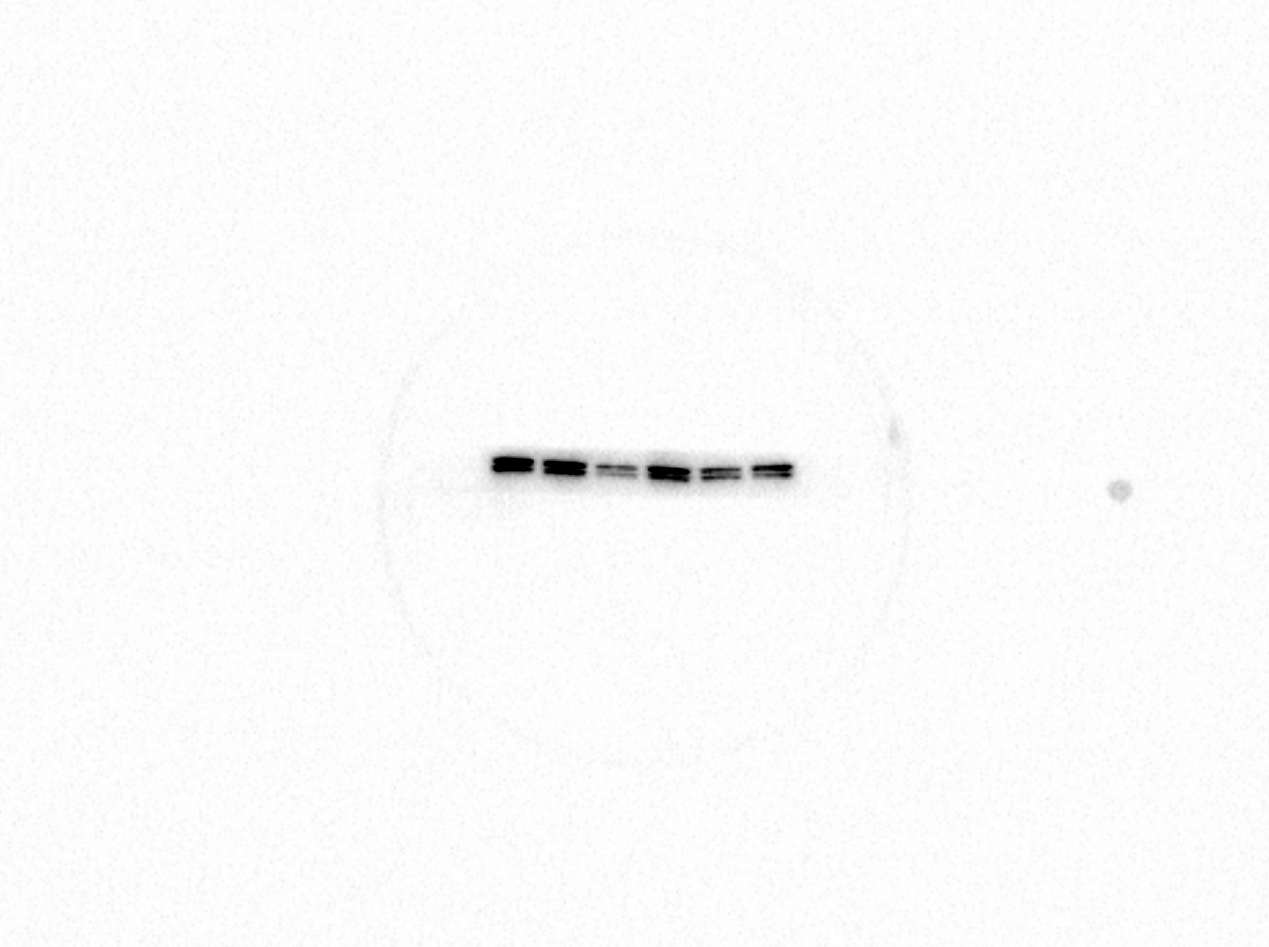

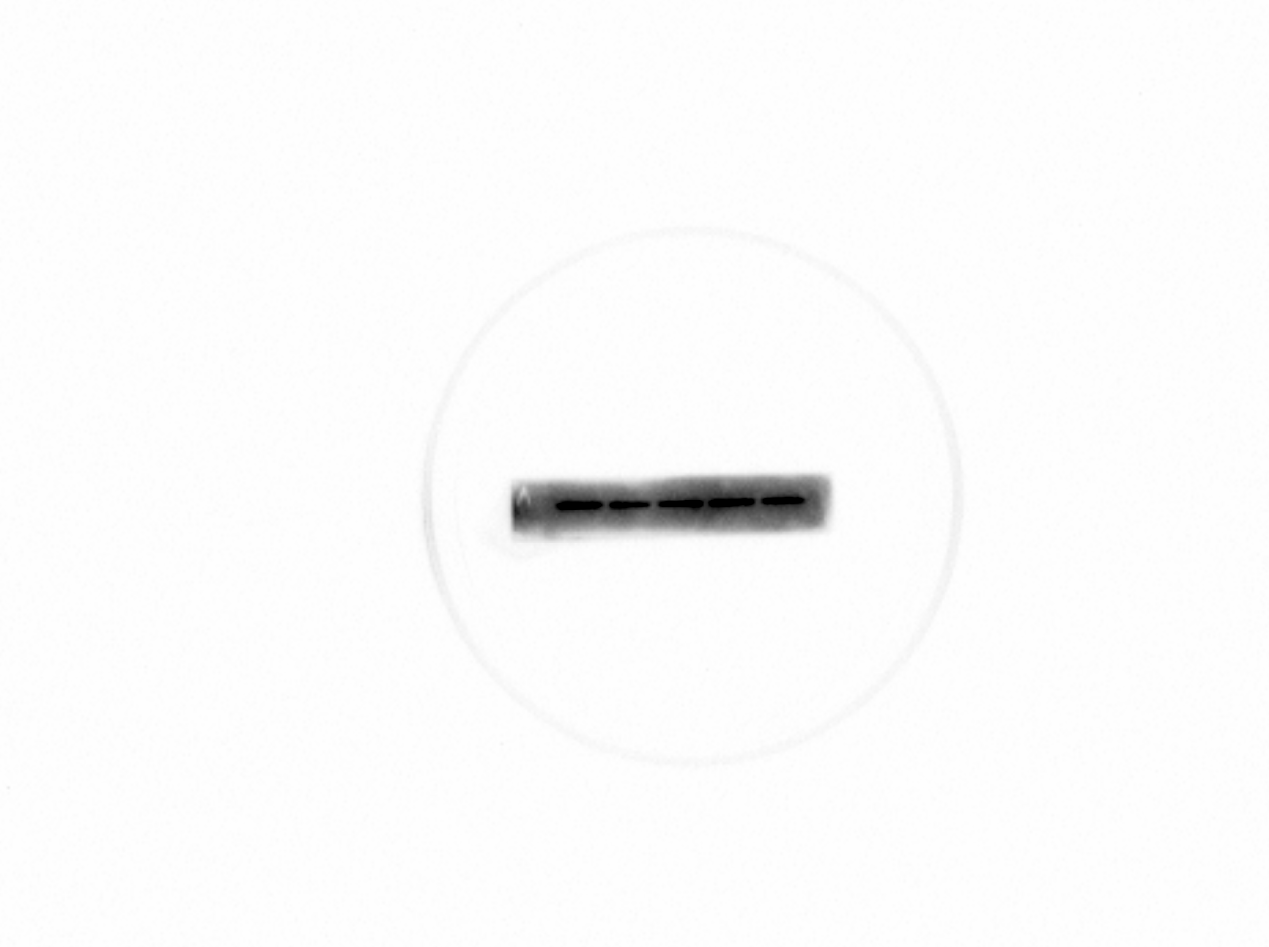

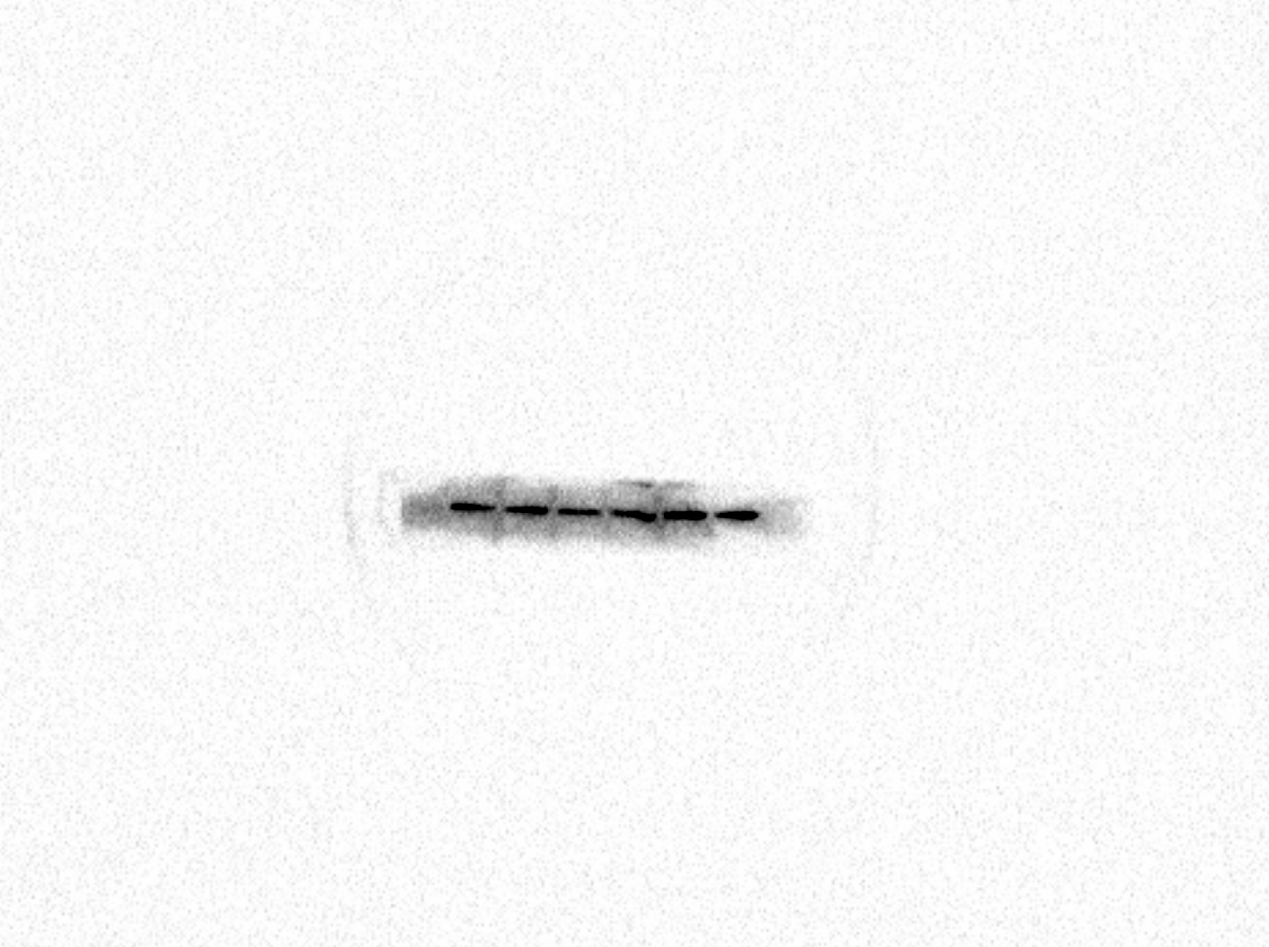


SMO-A549

SMO-A549

SHV

SOX2-OT #4

SOX2-OT #5

SHV

GLI1 #8

GLI1 #9

α-Tublin-A549

SHV

SOX2-OT #4

SOX2-OT #5

α-Tublin-A549

SHV

GLI1 #8

GLI1 #9


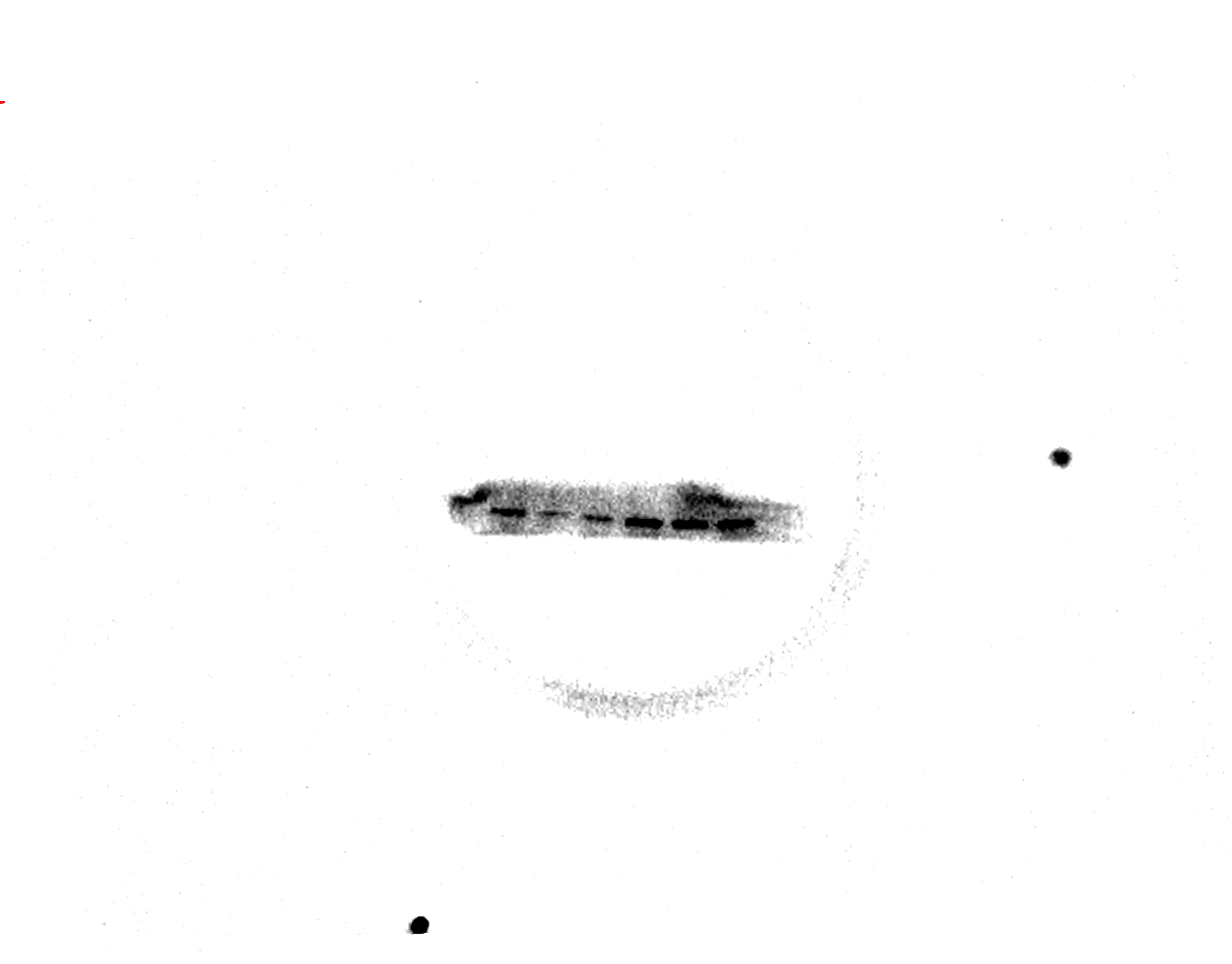


GLI1-H1299

SHV

SOX2-OT #4

SOX2-OT #5


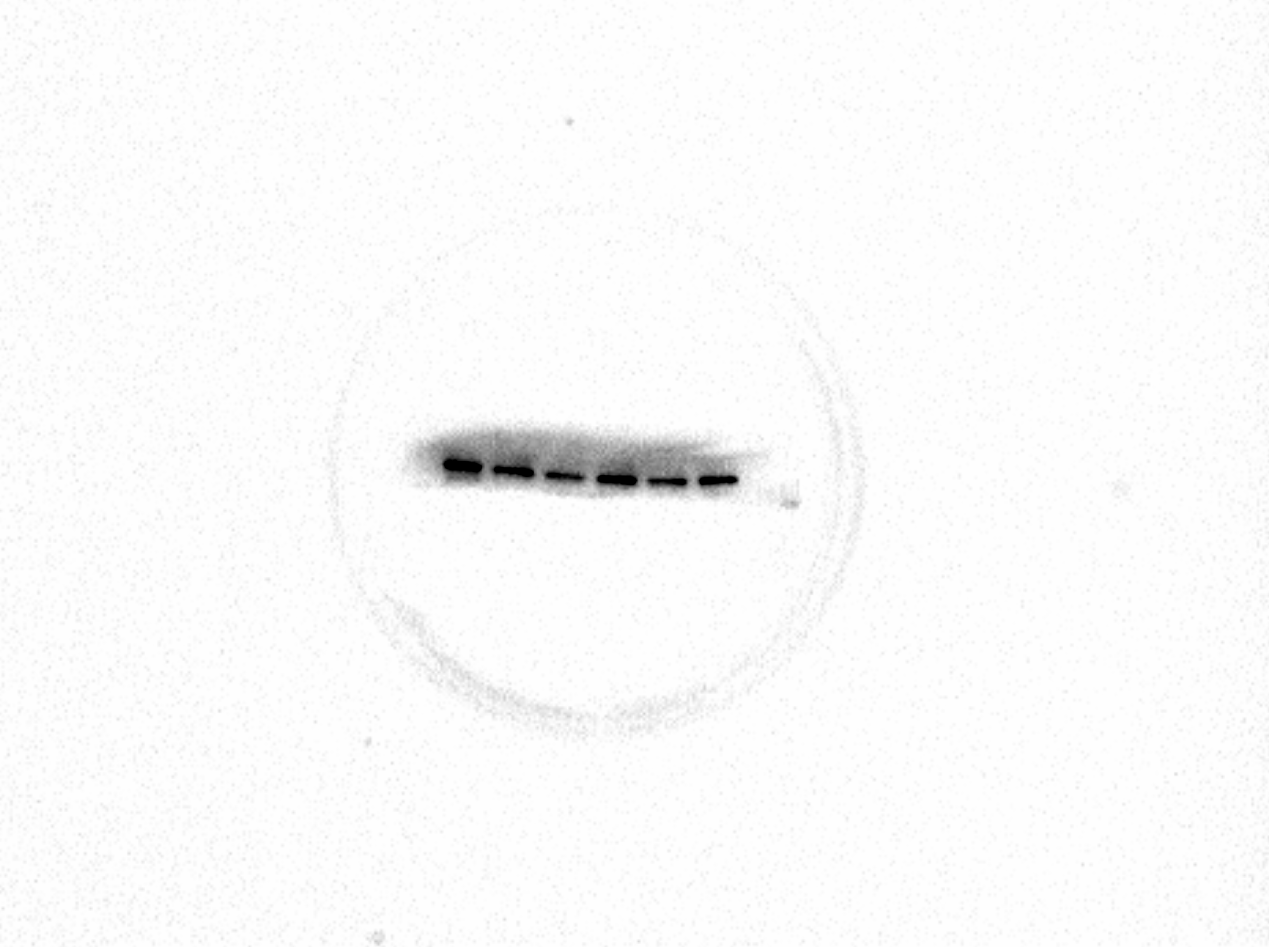


GLI1-H1299

SHV

GLI1 #8

GLI1 #9





SOX2-H1299

SHV

GLI1 #8

GLI1 #9

SHV

SOX2-OT #4

SOX2-OT #5





SOX2-H1299





SMO-H1299

SHV

SOX2-OT #4

SOX2-OT #5


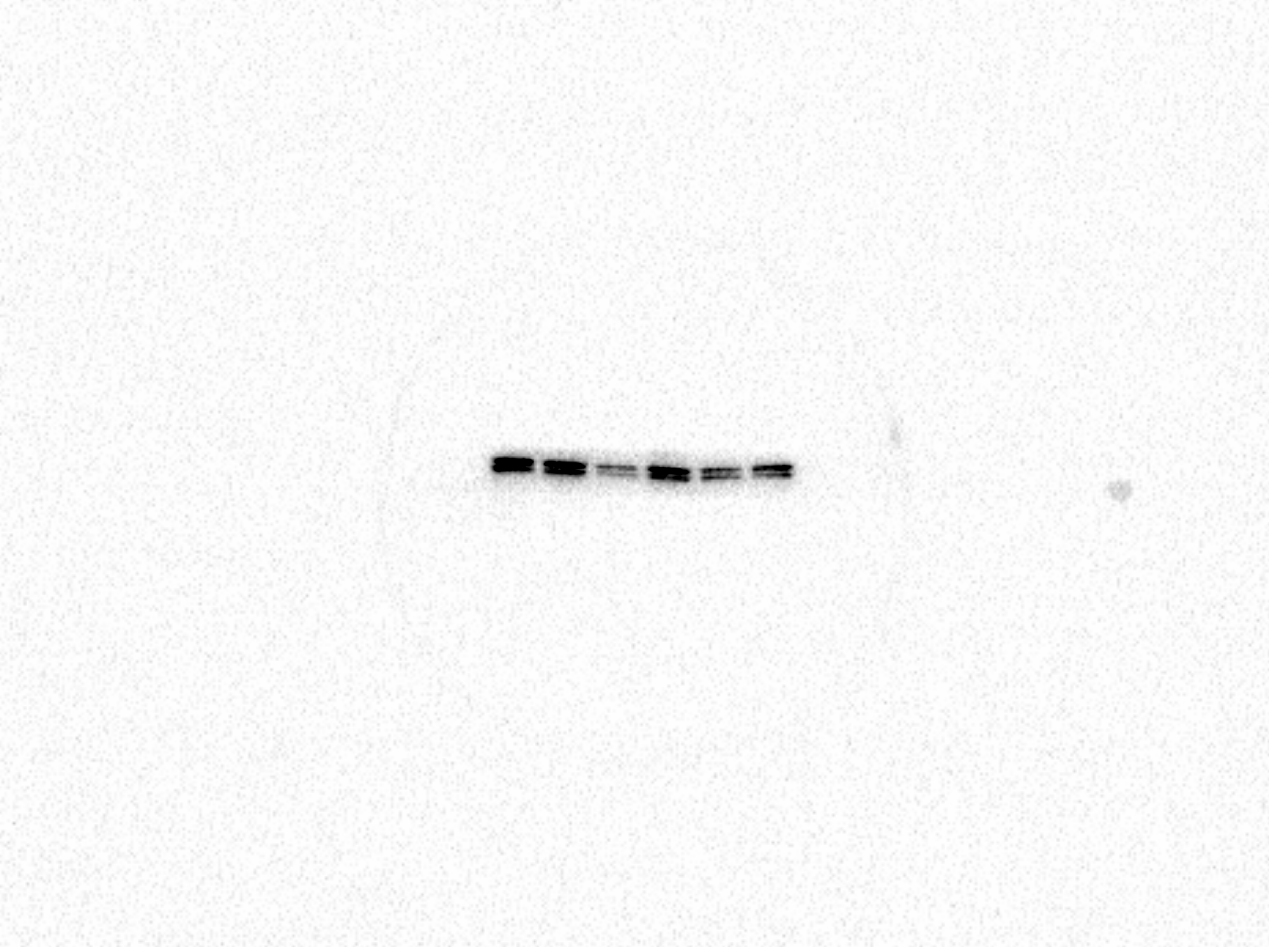


SMO-H1299

SHV

GLI1 #8

GLI1 #9


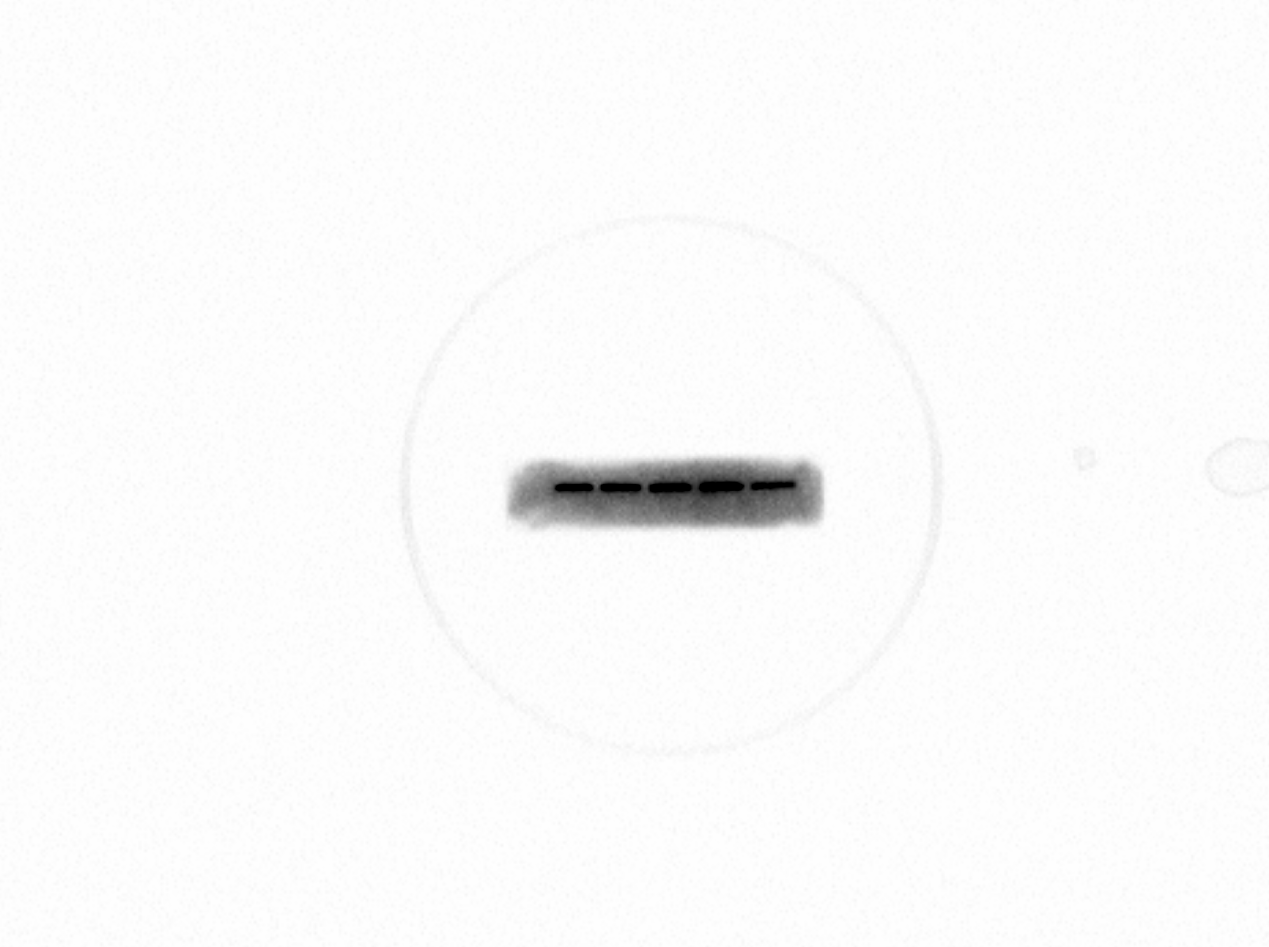


α-Tublin-H1299

SHV

SOX2-OT #4

SOX2-OT #5


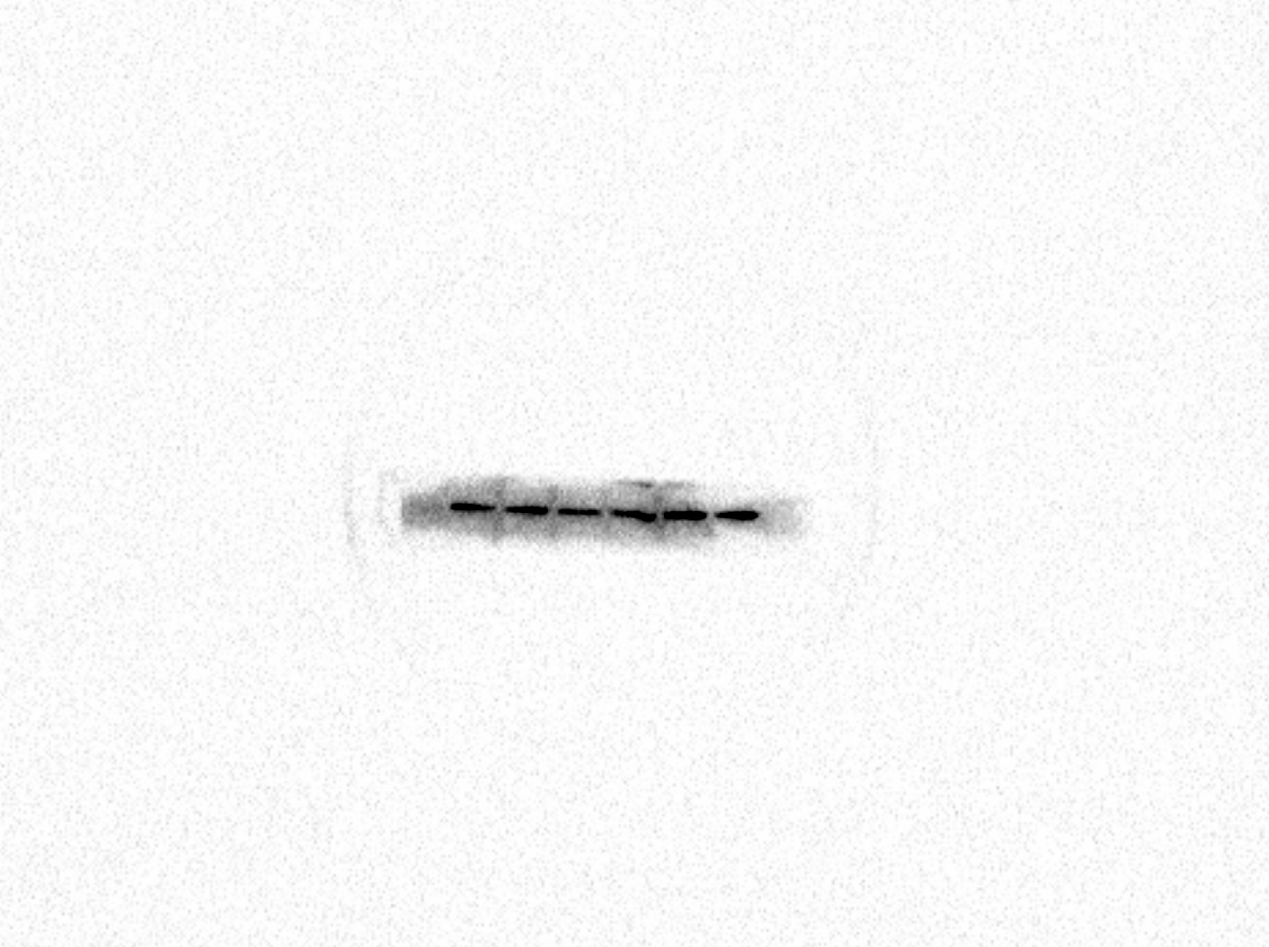


α-Tublin-H1299

SHV

GLI1 #8

GLI1 #9

FIG4D


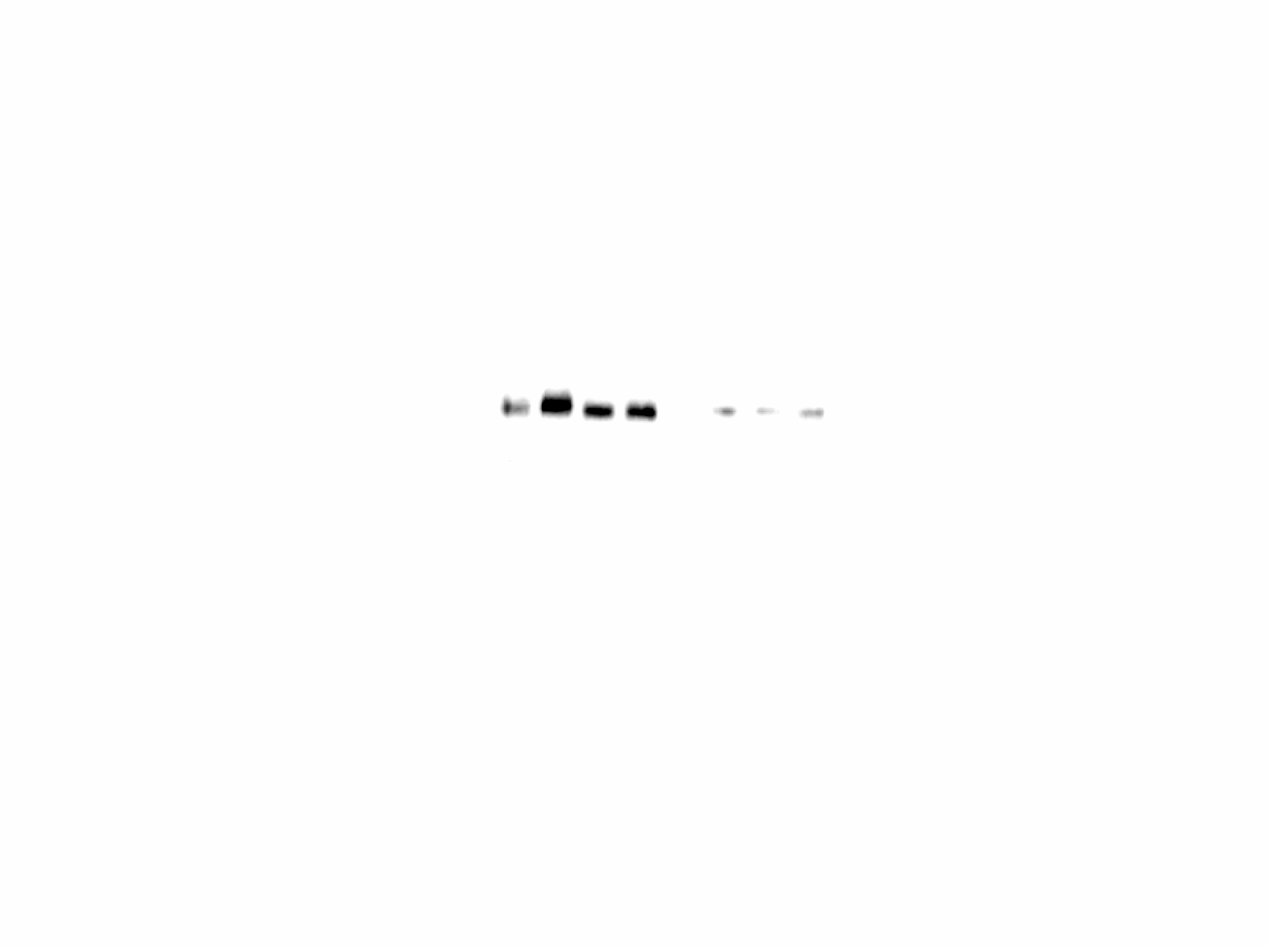


GLI1-A549

pENTER

METTL3

METTL14

IGF2BL2

GLI1 #9


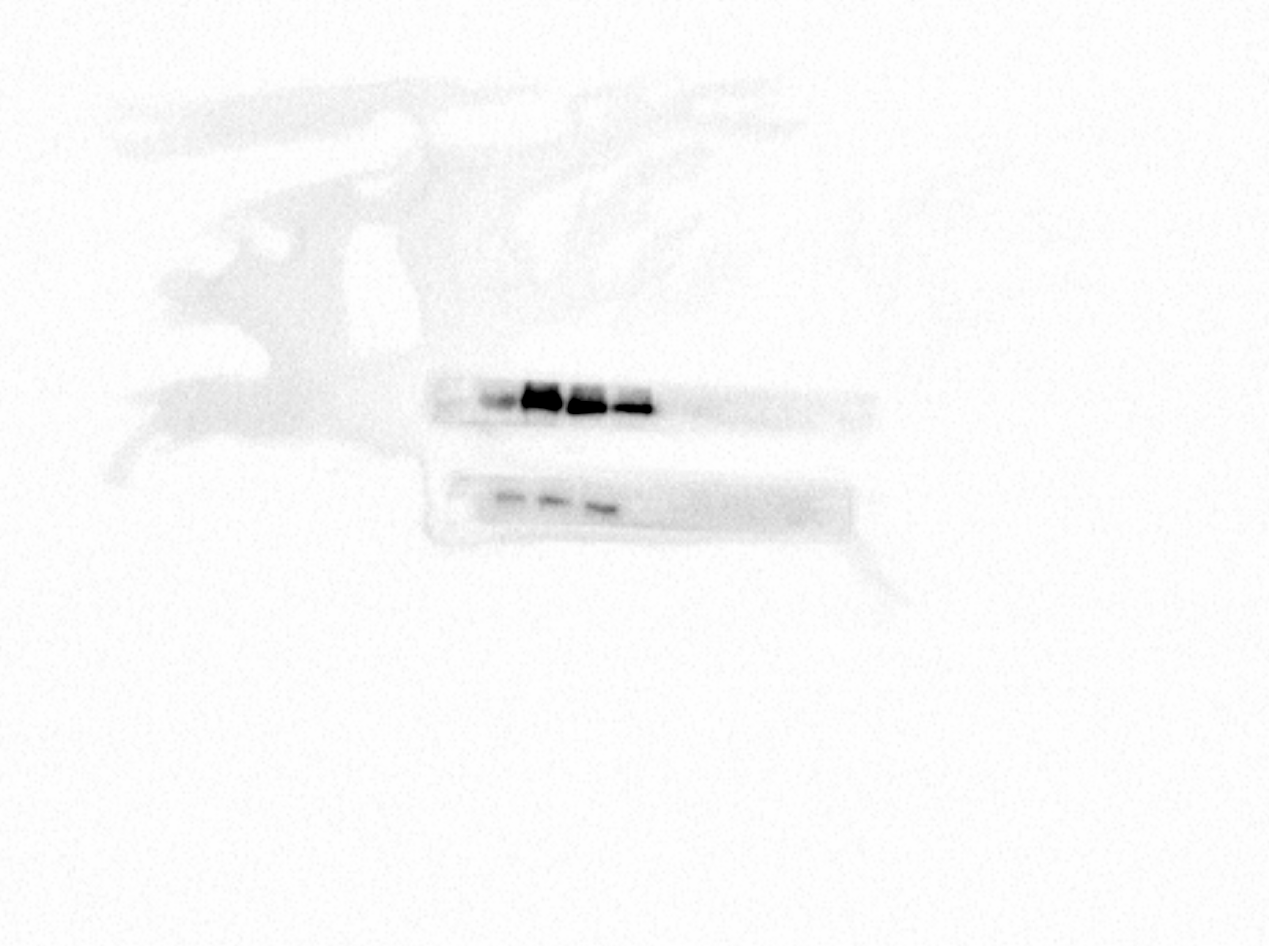


METTL3-A549

pENTER

METTL3

METTL14

IGF2BL2

GLI1 #9


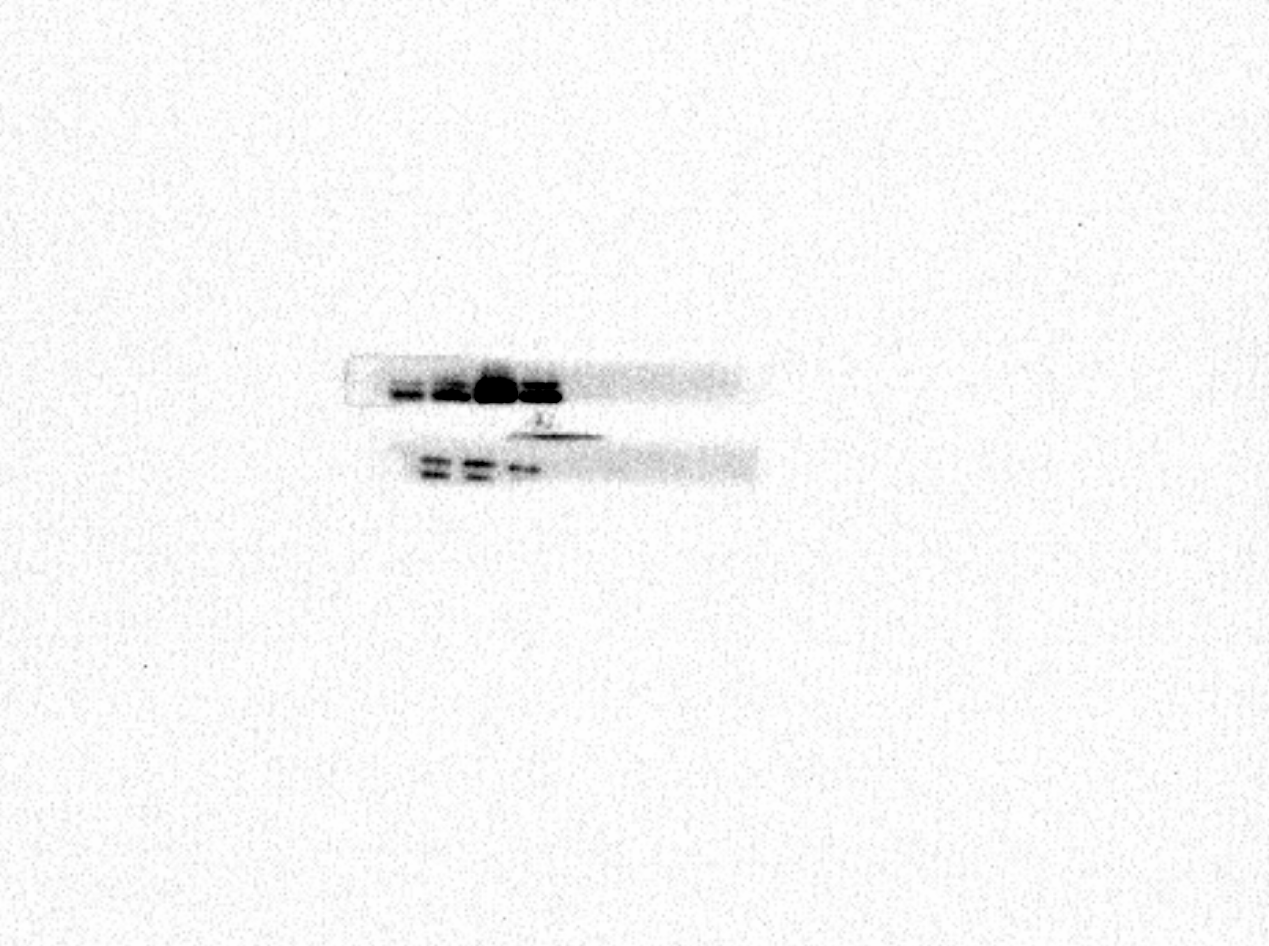


METTL14-A549

pENTER

METTL3

METTL14

IGF2BL2

GLI1 #9


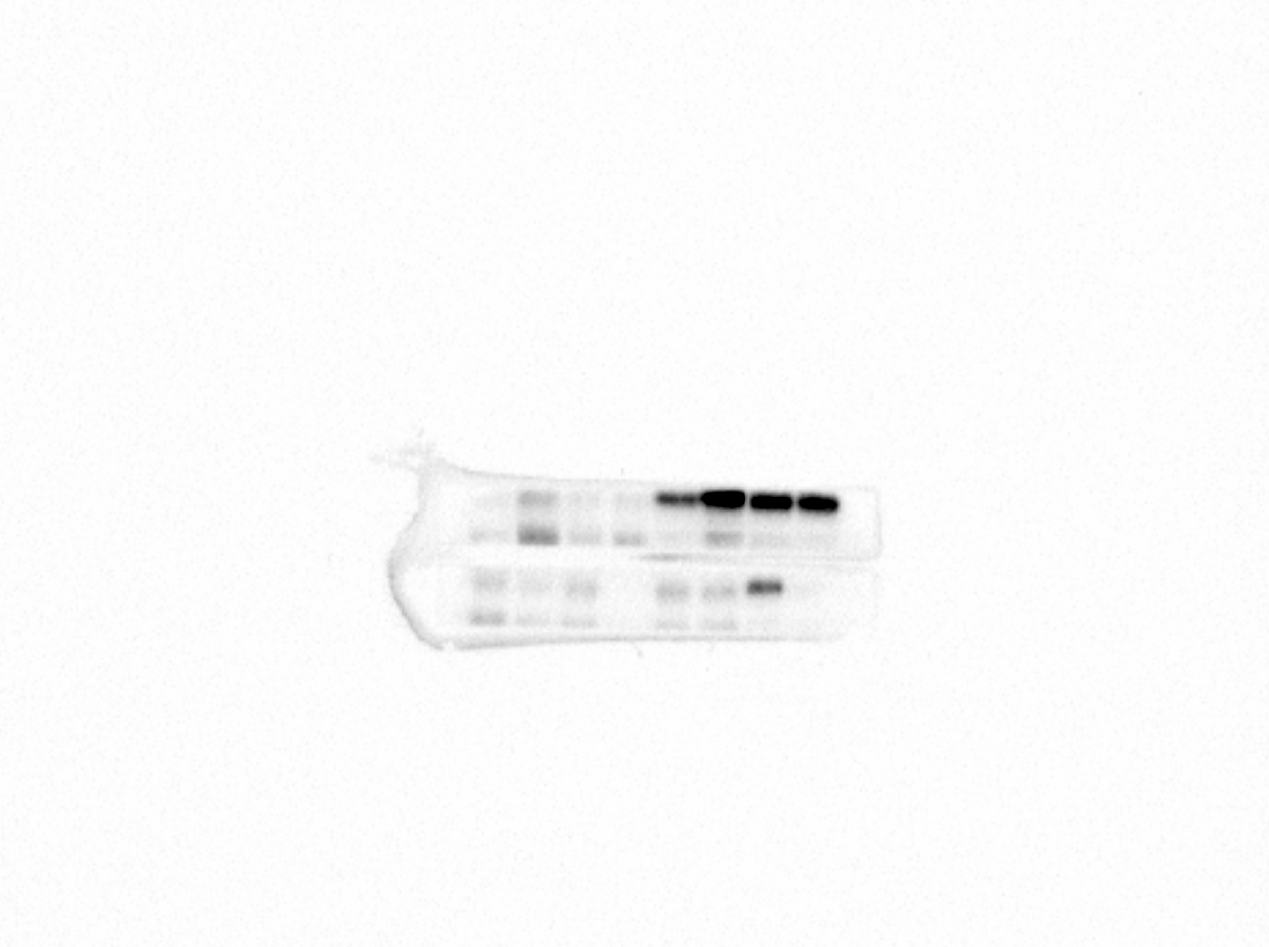


IGF2BP2-A549

pENTER

METTL3

METTL14

IGF2BL2

GLI1 #9


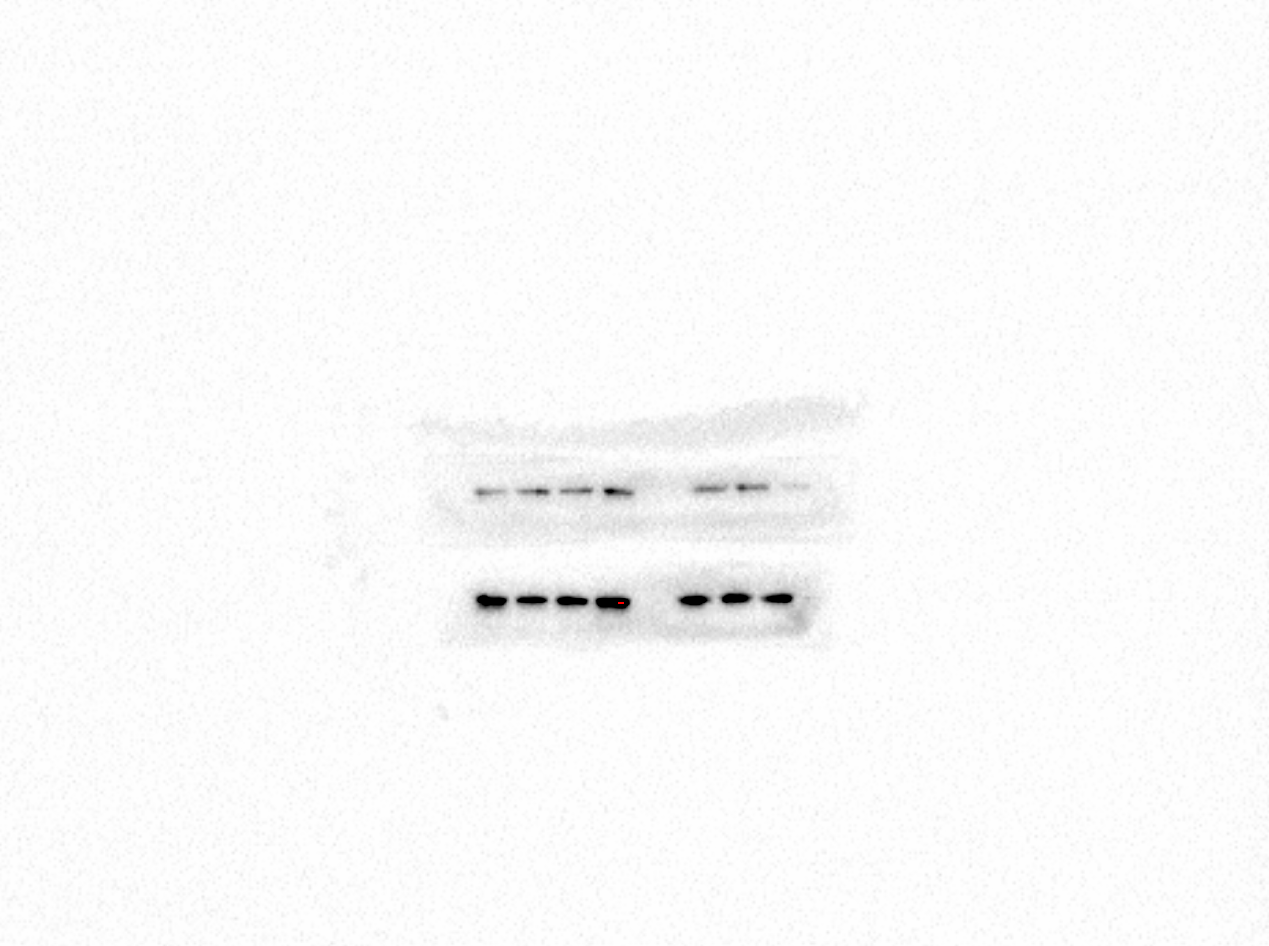


β-Actin-A549

pENTER

METTL3

METTL14

IGF2BL2

GLI1 #9


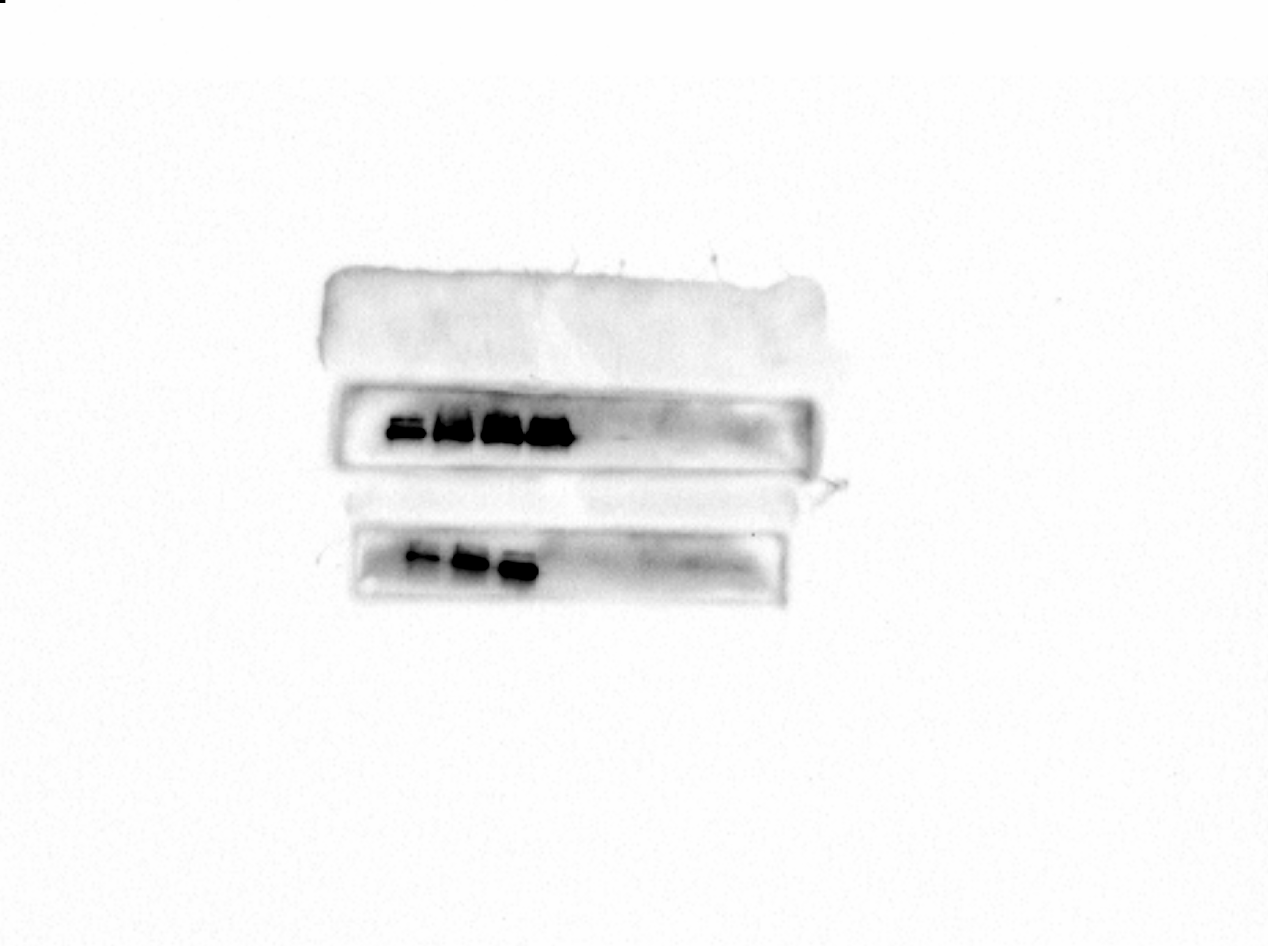


GLI1-H1299

pENTER

METTL3

METTL14

IGF2BL2

GLI1 #9


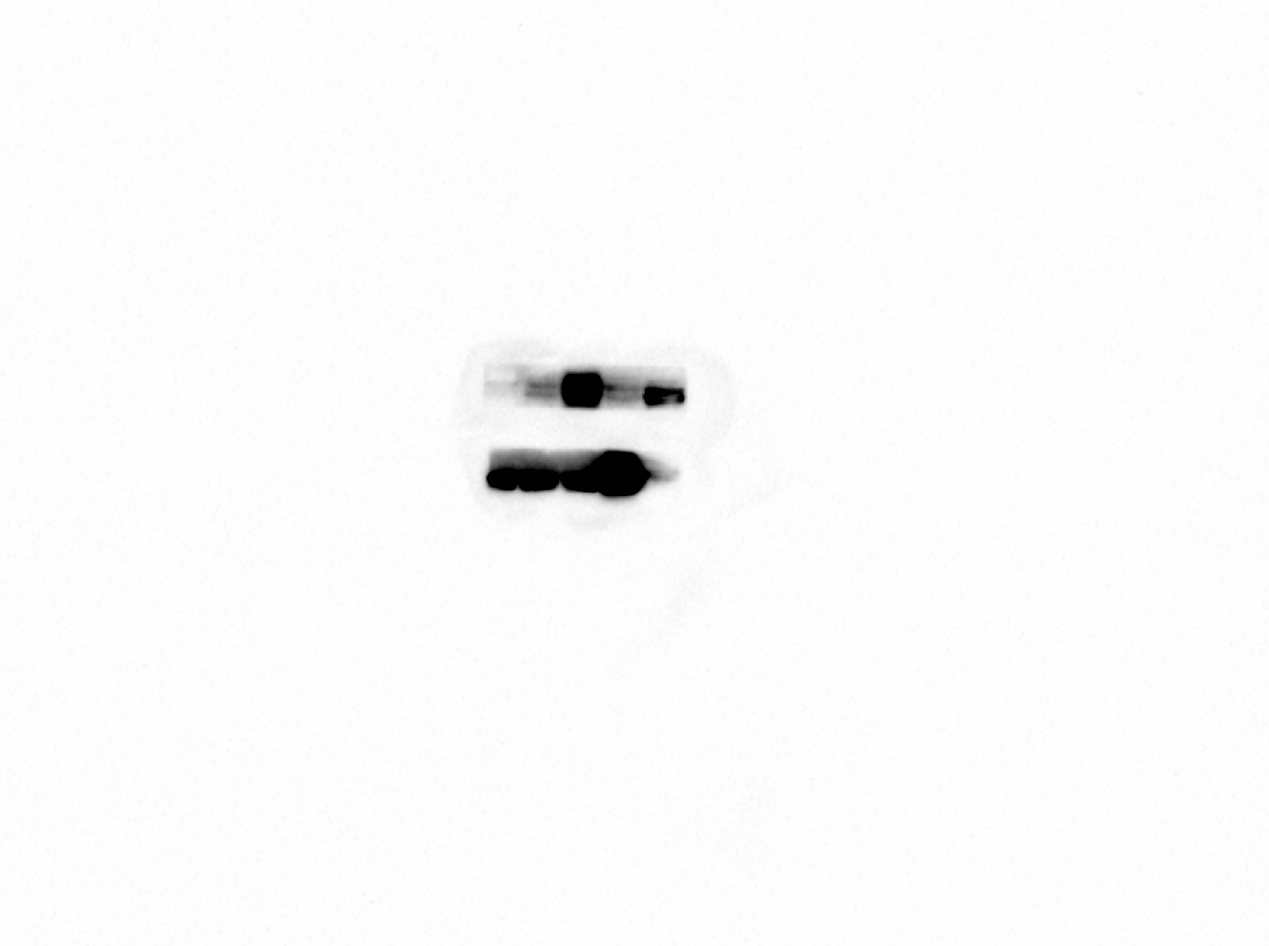


METTL3-H1299

pENTER

METTL3

METTL14

IGF2BL2

GLI1 #9


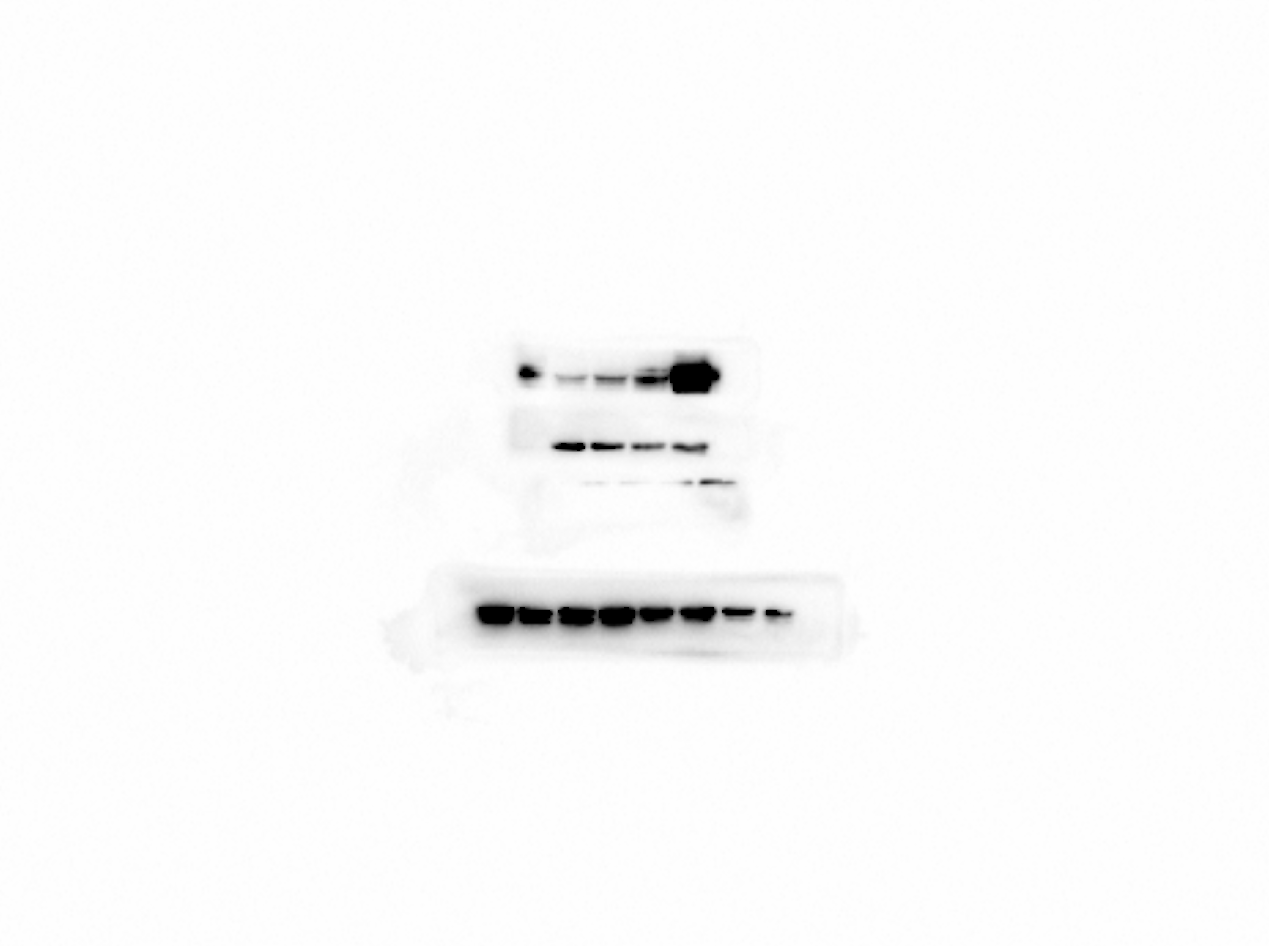


METTL14-H1299

pENTER

METTL3

METTL14

IGF2BL2

GLI1 #9


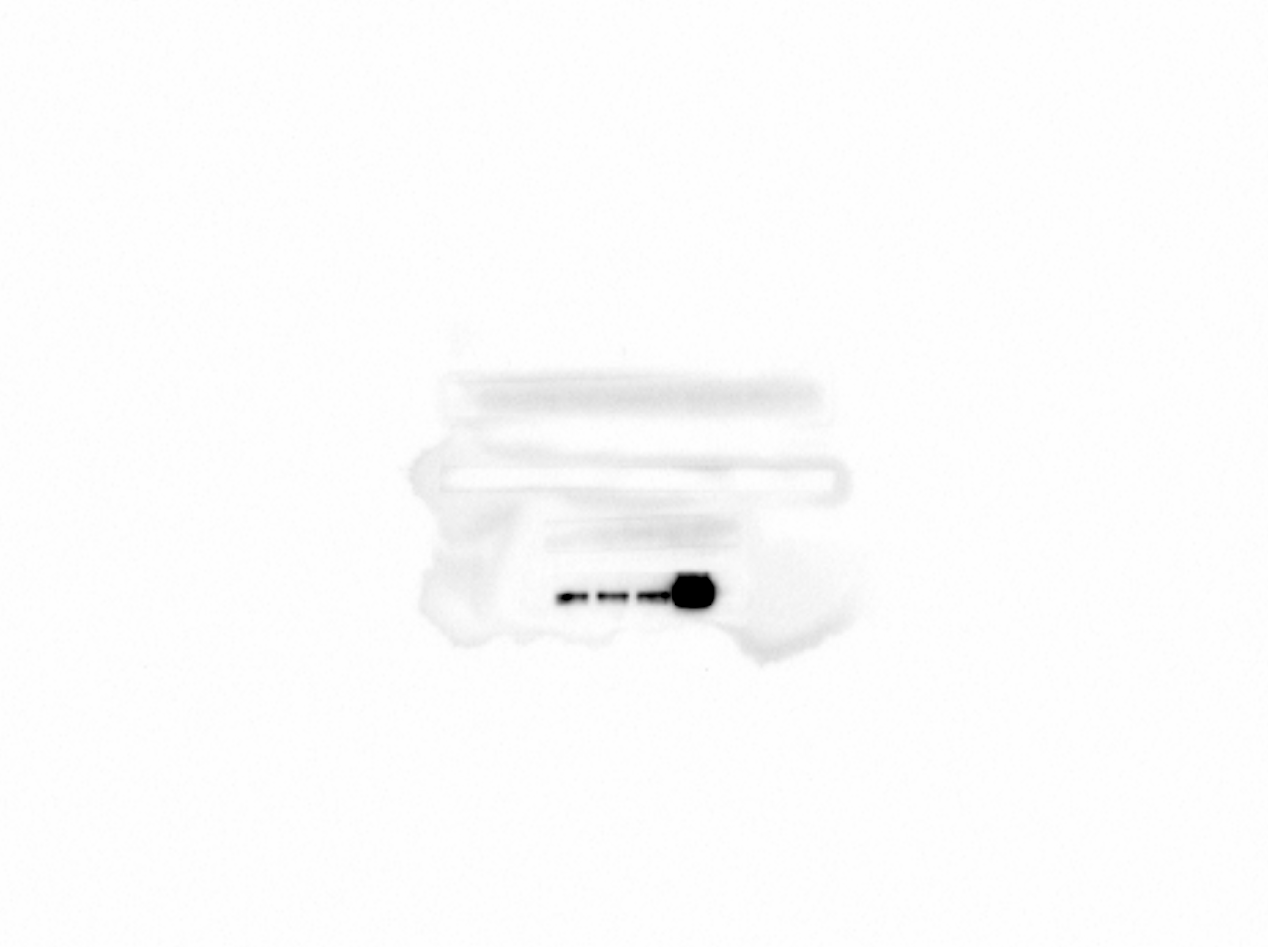


IGF2BP2-H1299

pENTER

METTL3

METTL14

IGF2BL2

GLI1 #9


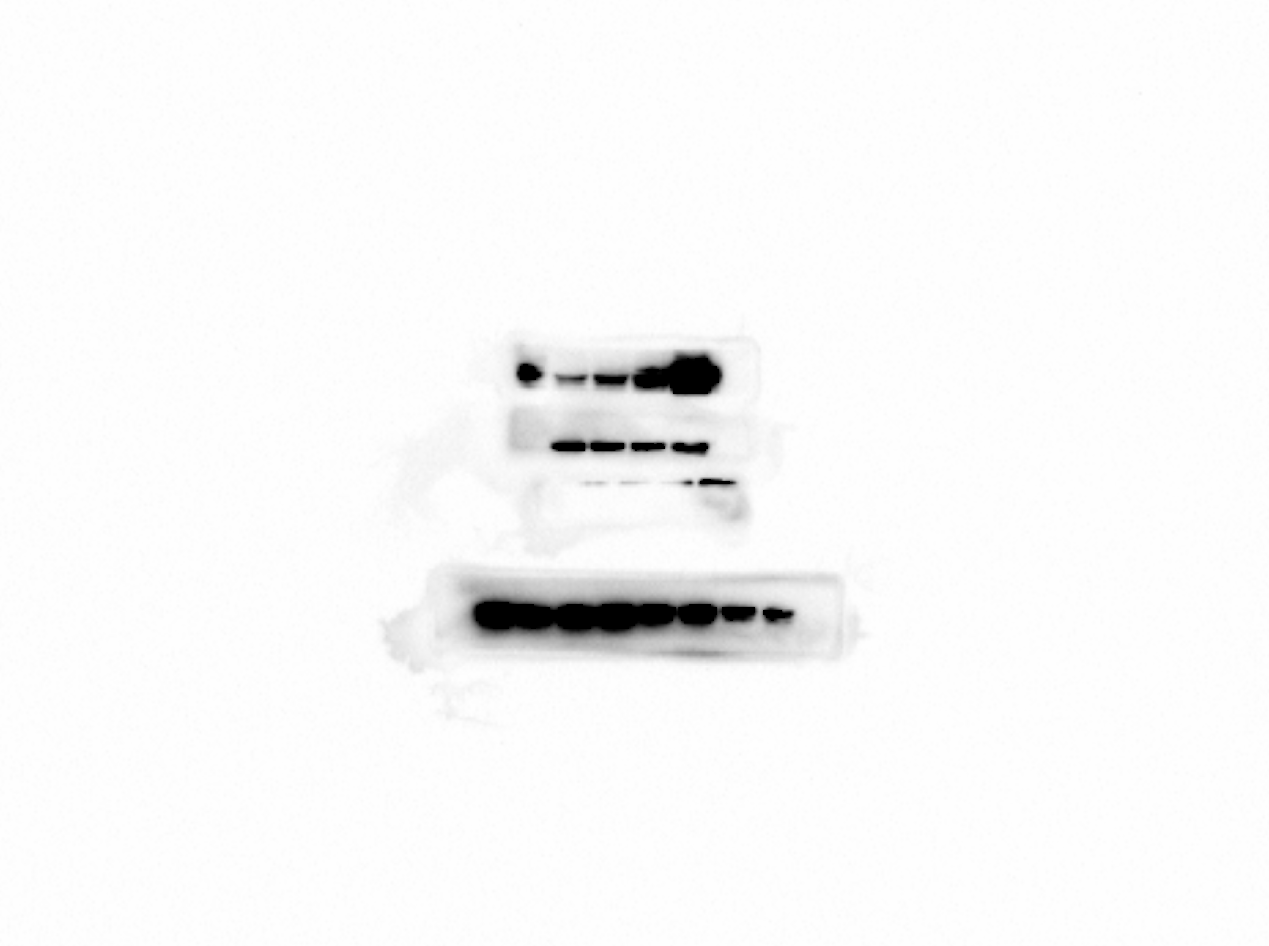


β-Actin-H1299

pENTER

METTL3

METTL14

IGF2BL2

GLI1 #9

FIG4G

pENTER

METTL3

METTL3+#4


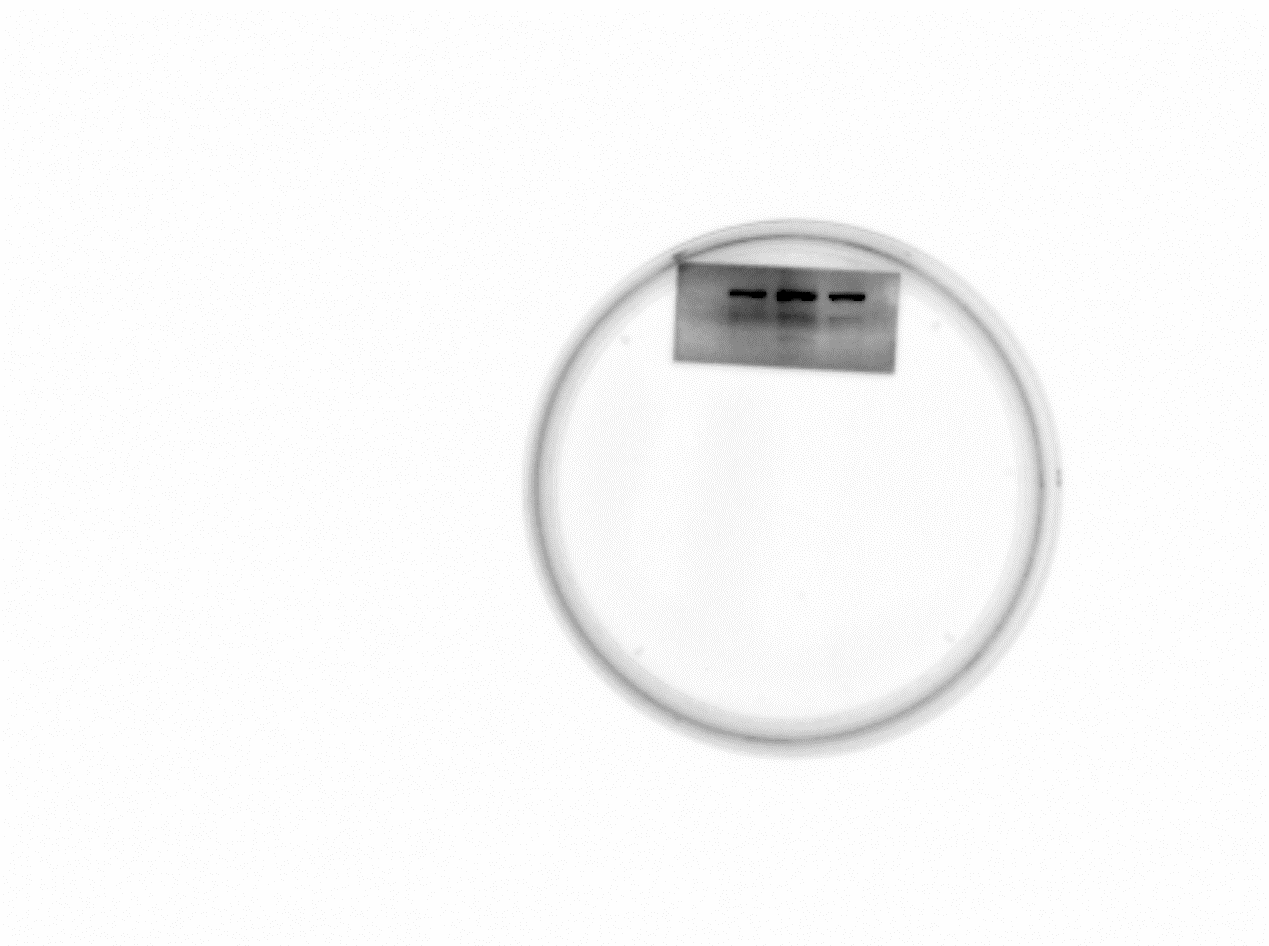


GLI1


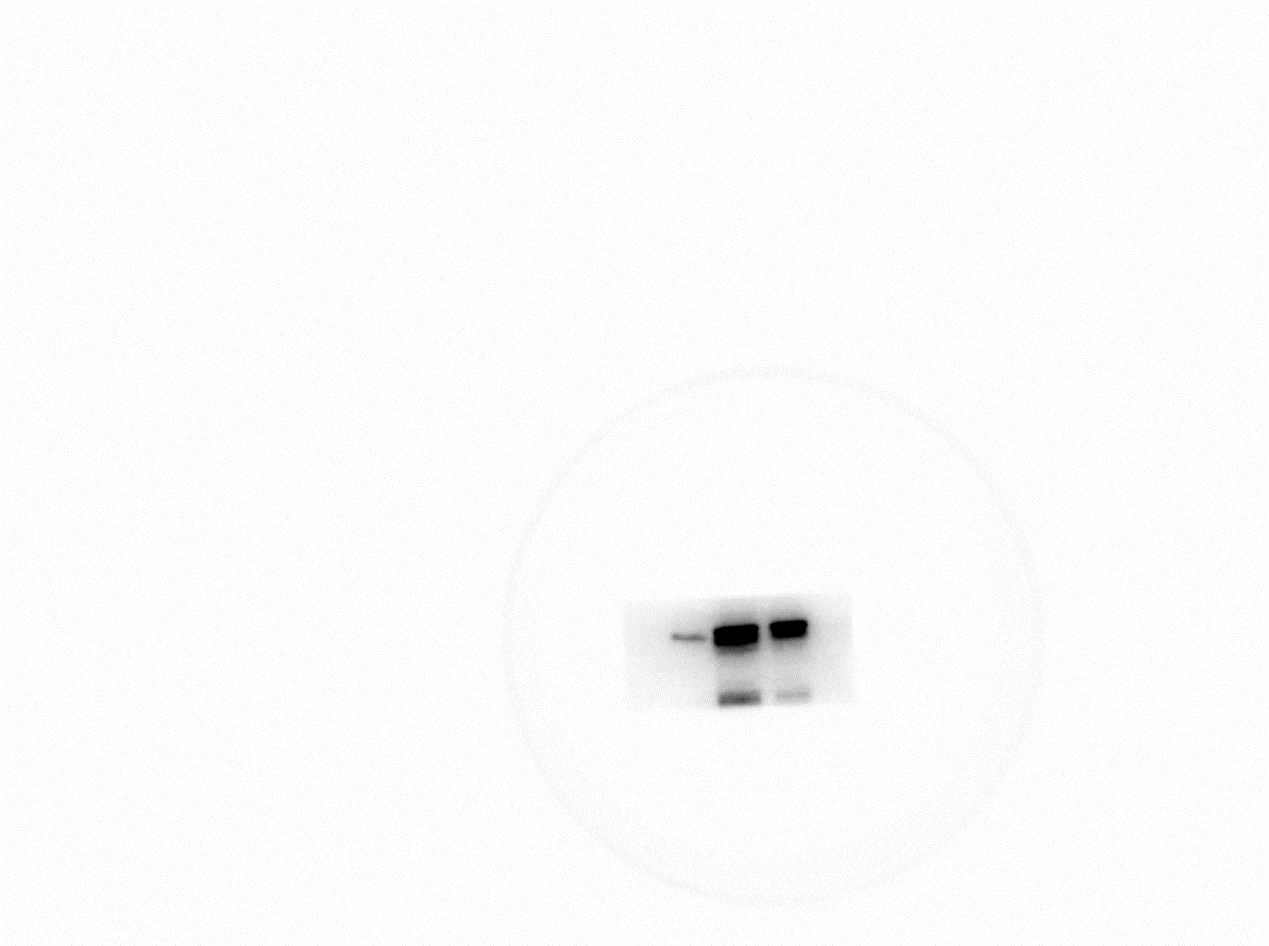


METTL3

pENTER

METTL3

METTL3+#4


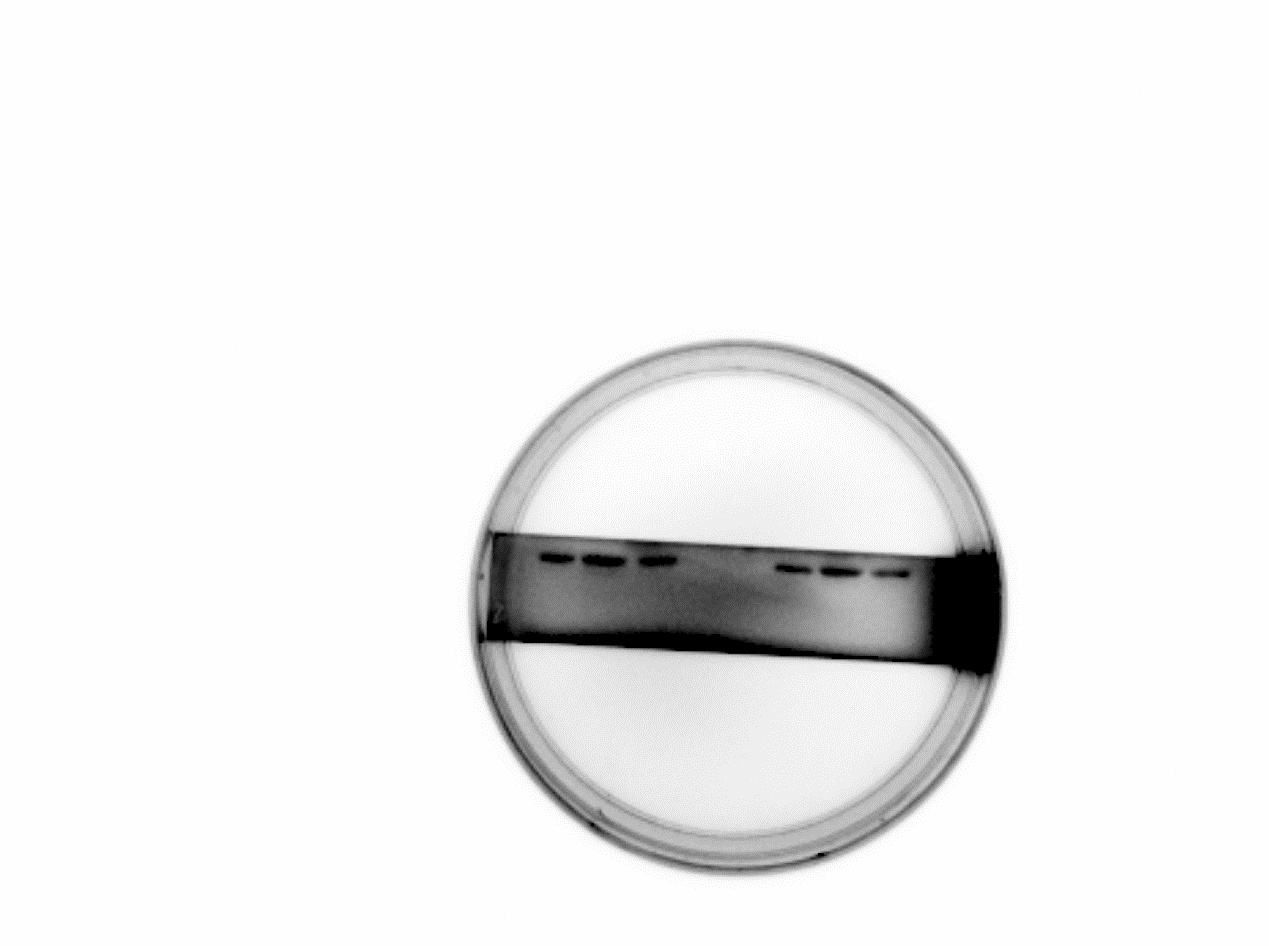


β-Actin

pENTER

METTL3

METTL3+#4


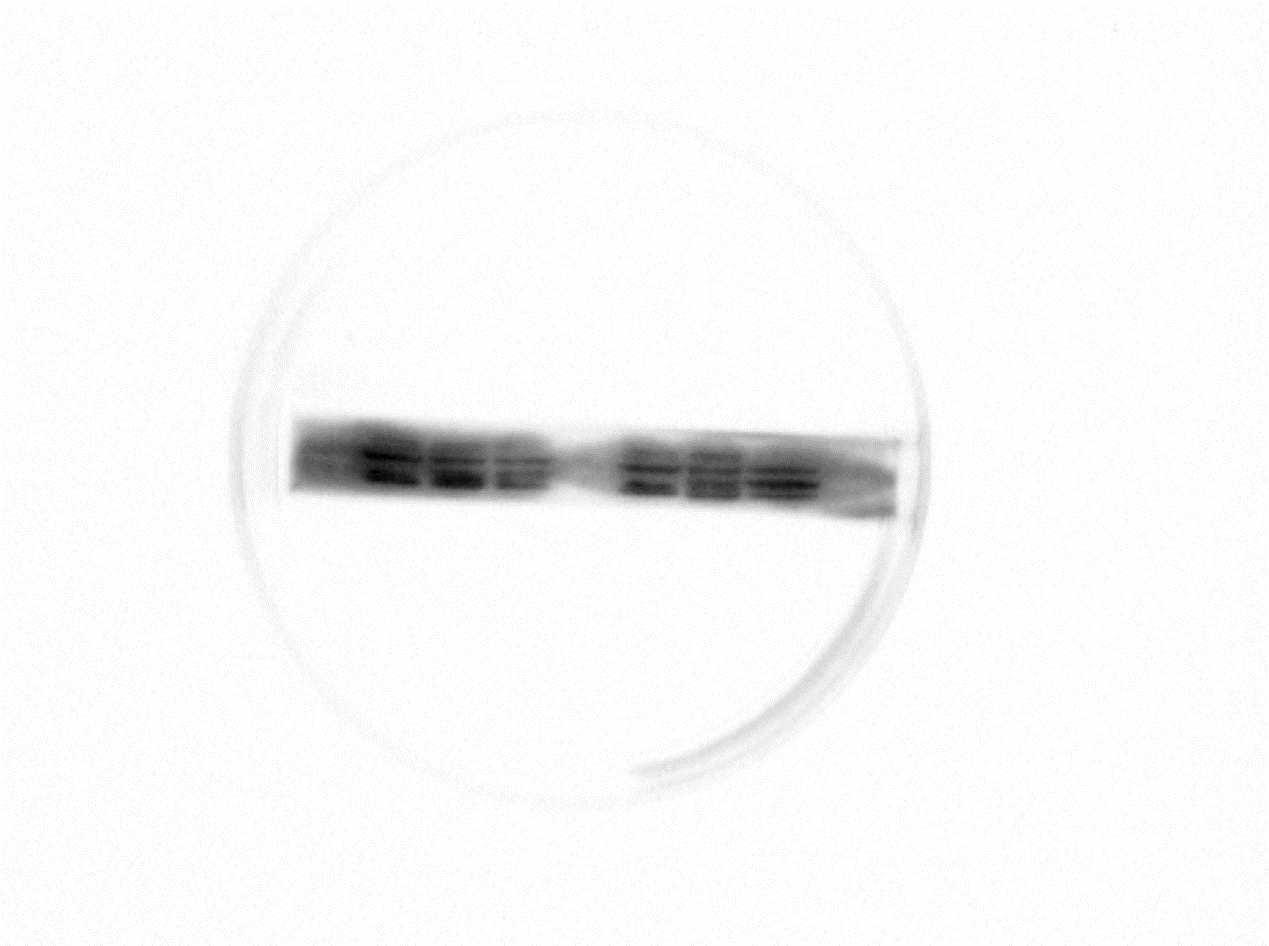


GLI1

pENTER

METTL14

METTL14+#4


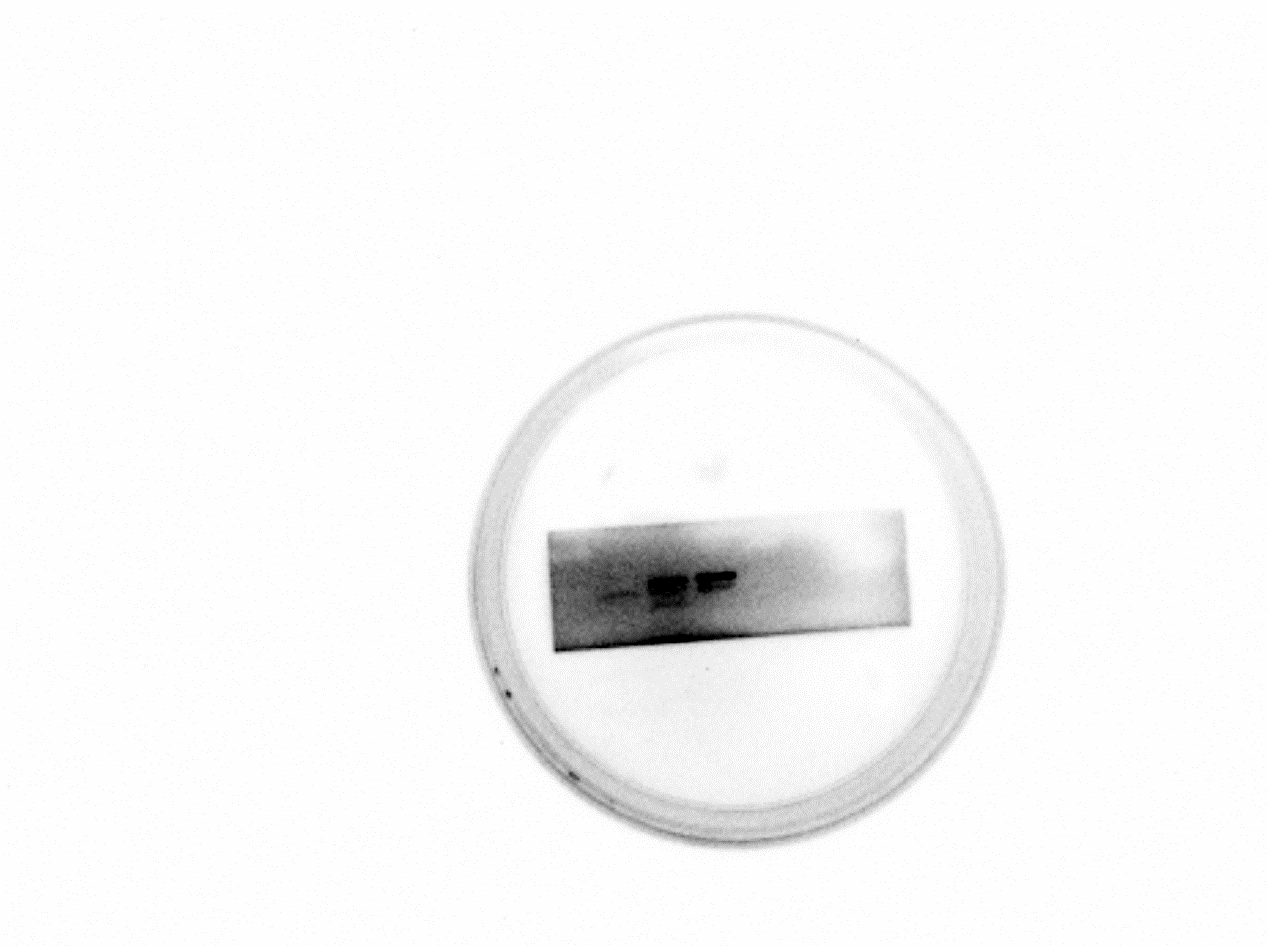


METTL14

pENTER

METTL14

METTL14+#4


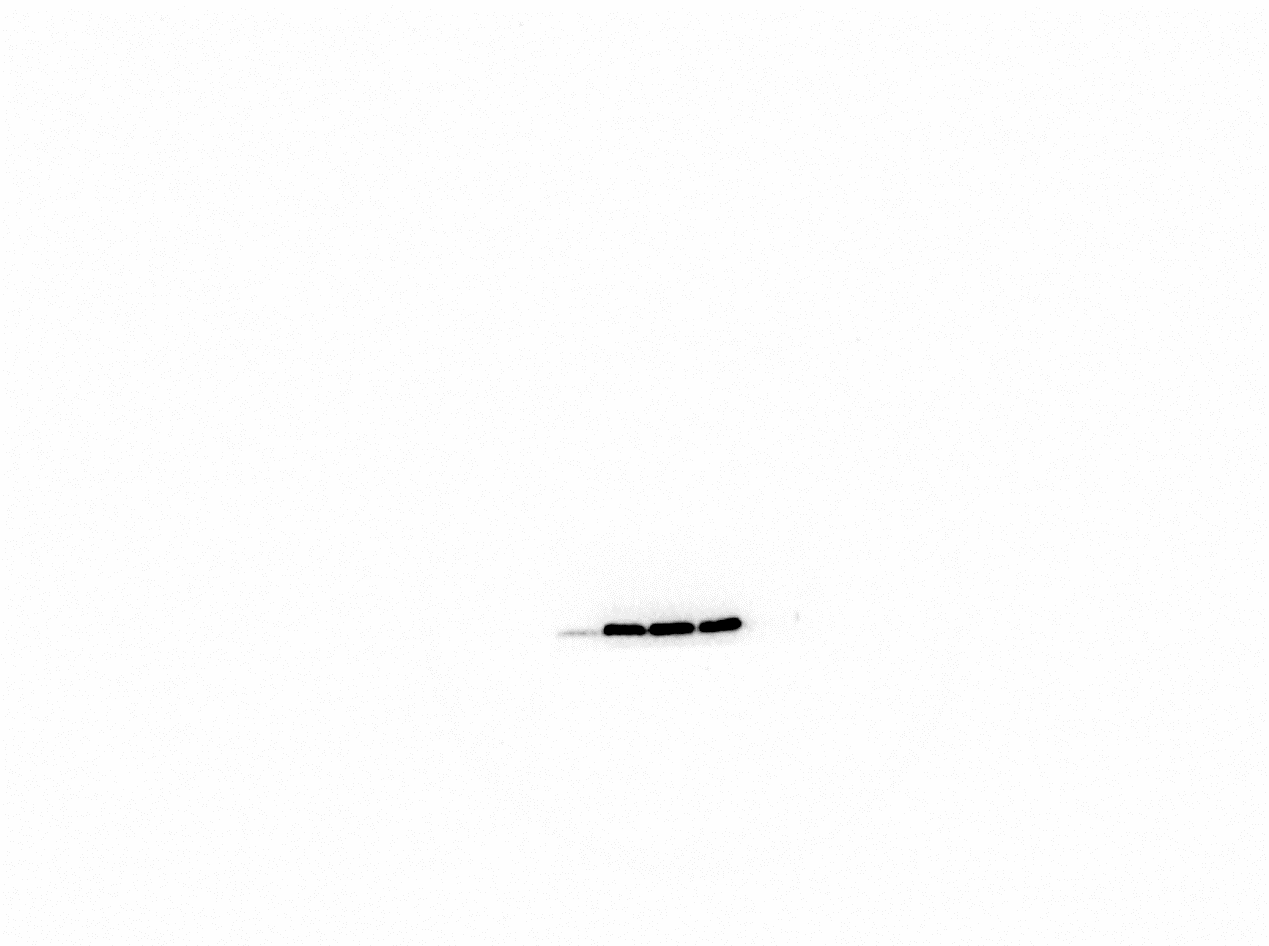


β-Actin

pENTER

METTL14

METTL14+#4


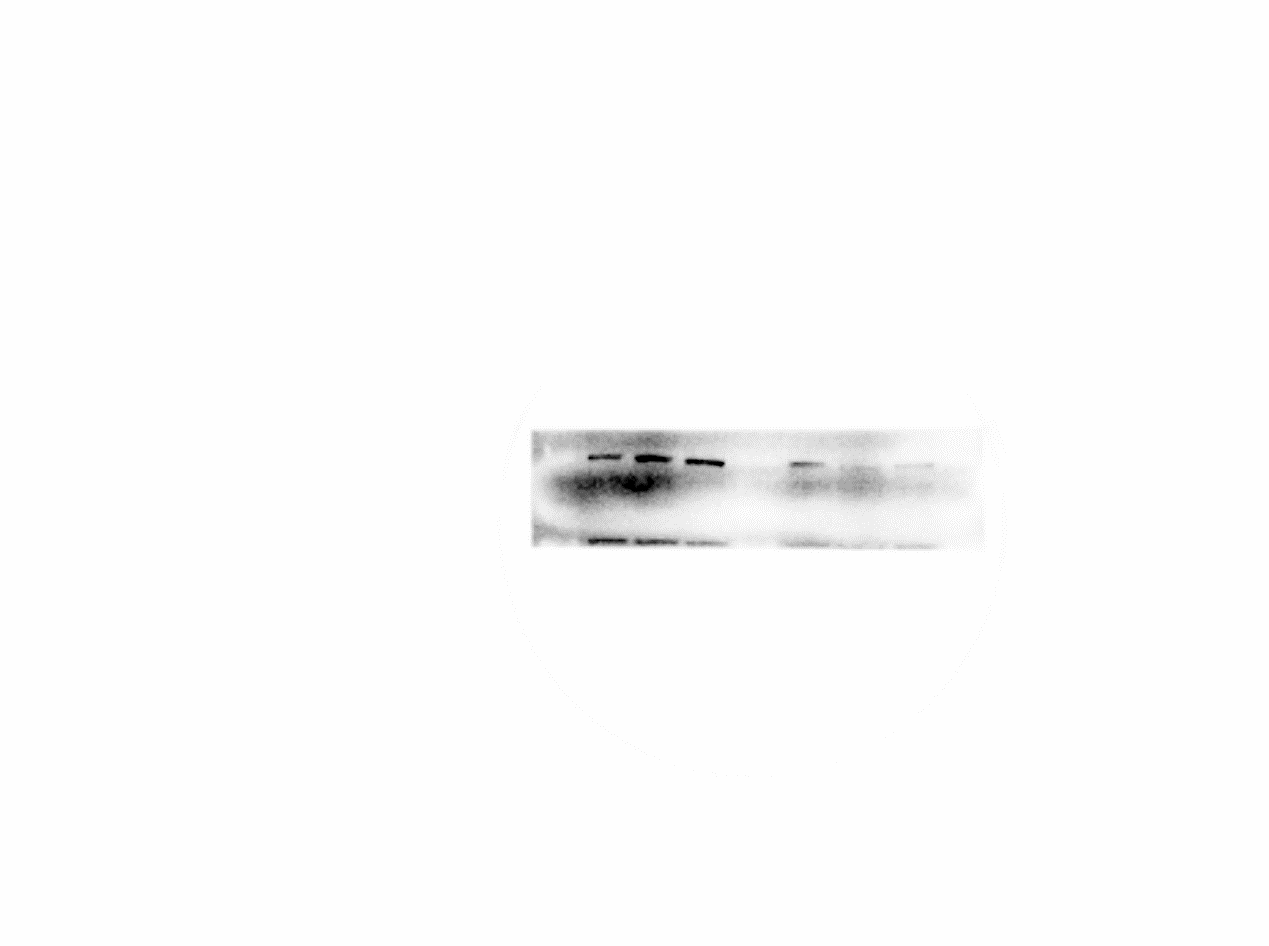


GLI1

pENTER

IFG2BP2

IGF2BP2+#4


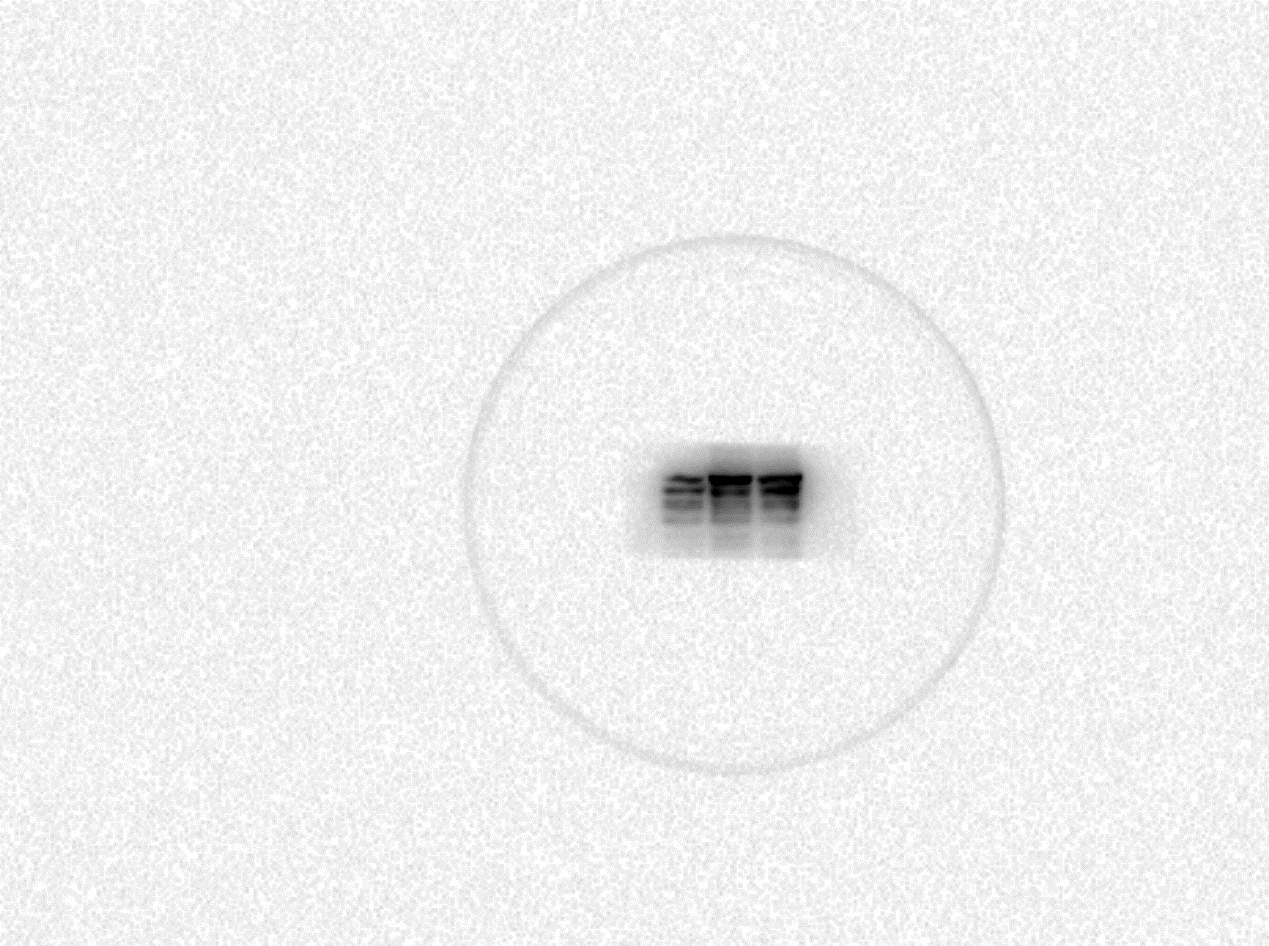


IGF2BP2

pENTER

IFG2BP2

IGF2BP2+#4


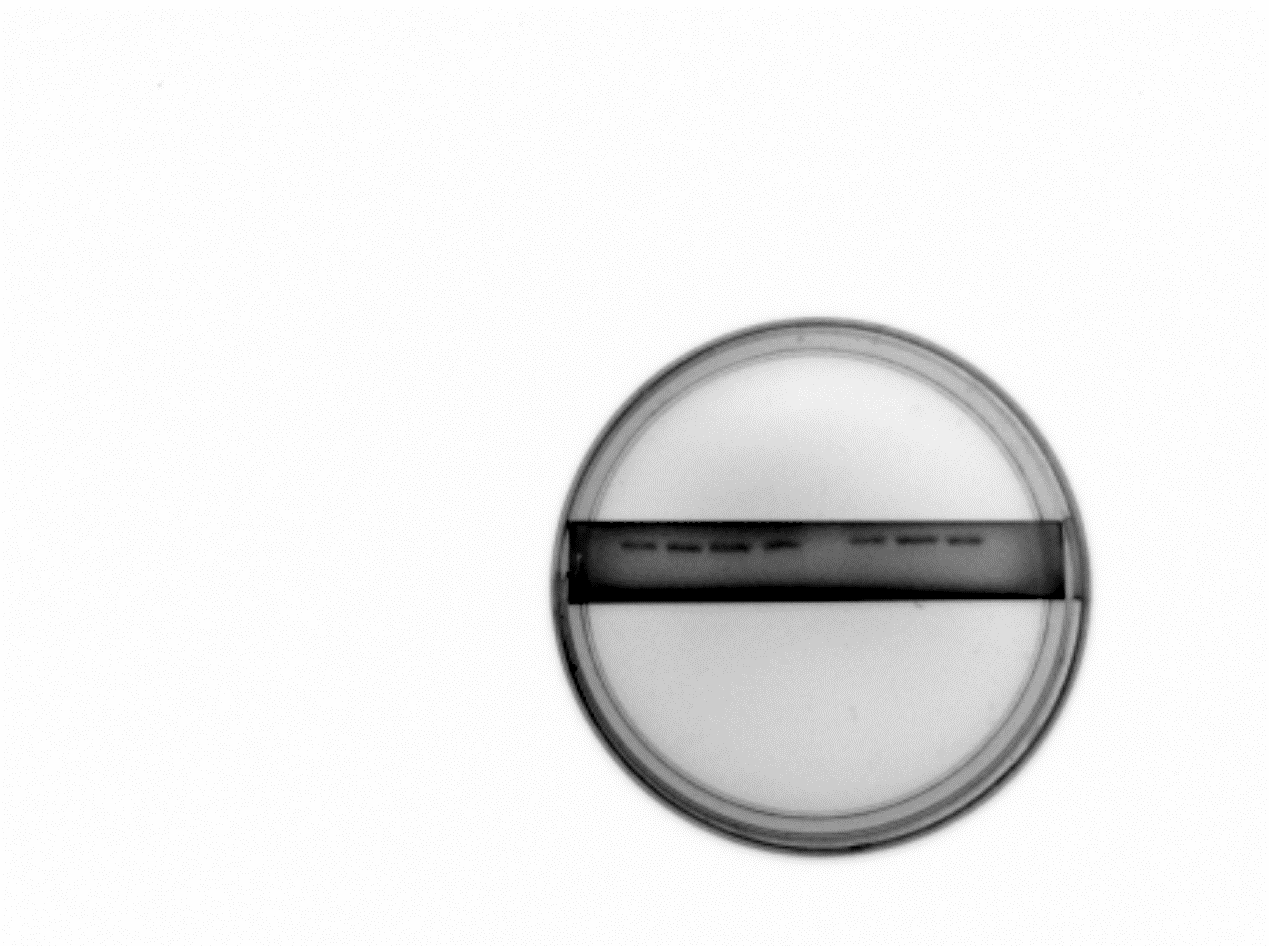


pENTER

IFG2BP2

IGF2BP2+#4

β-Actin

FIG6B
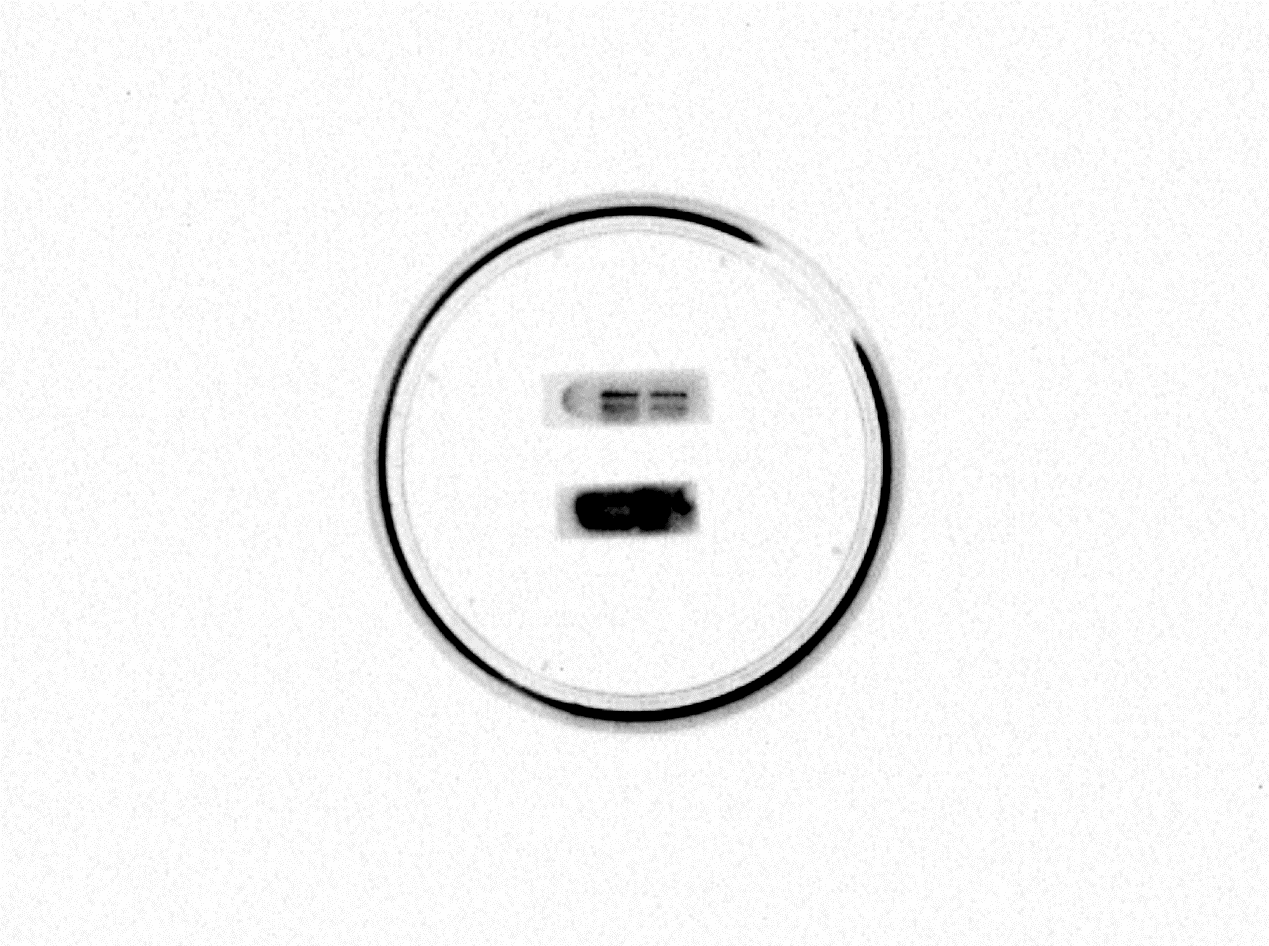


GLI1-A549

SHV

SOX2-OT#4


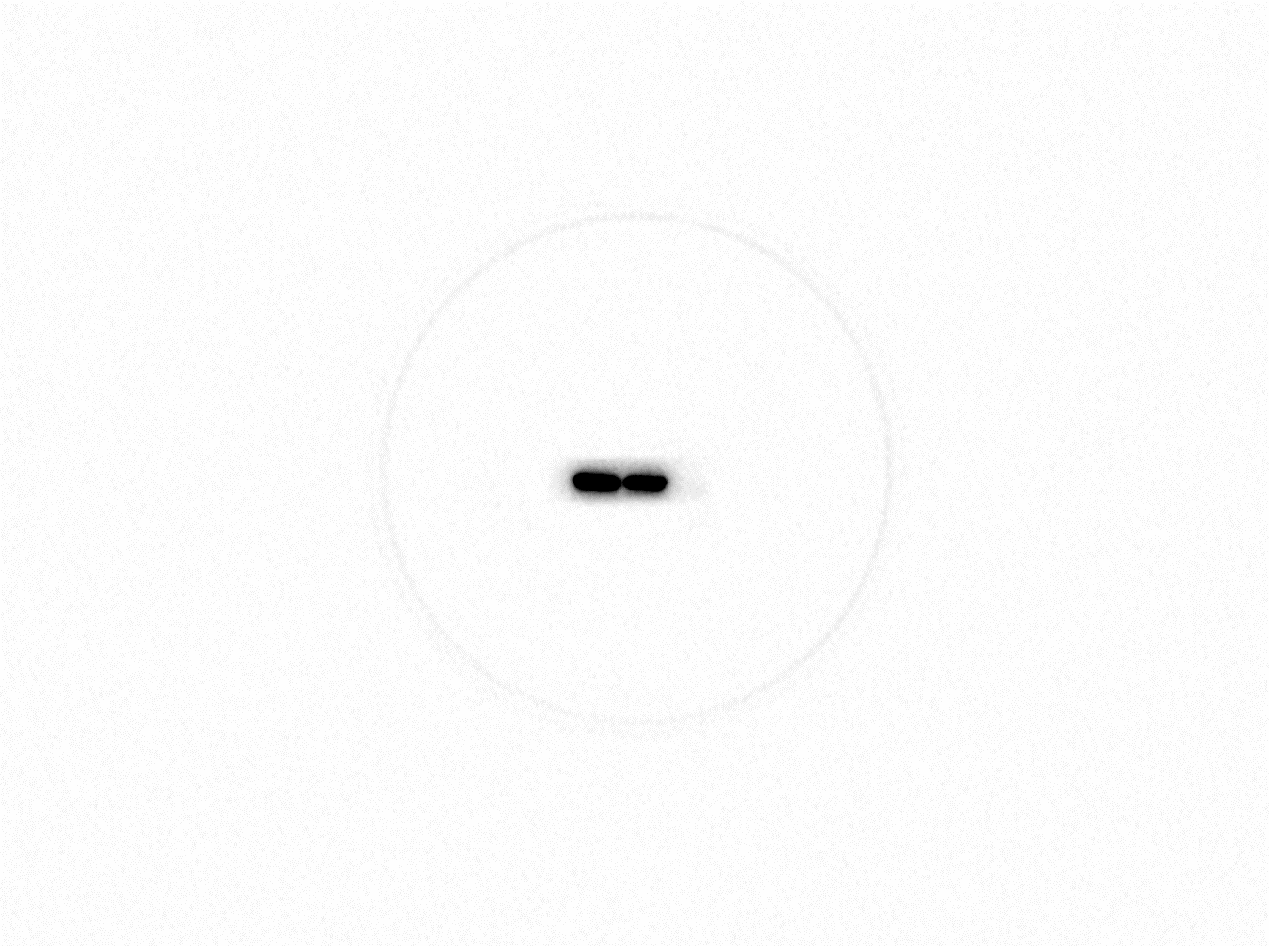


METTL3-A549

SHV

SOX2-OT#4


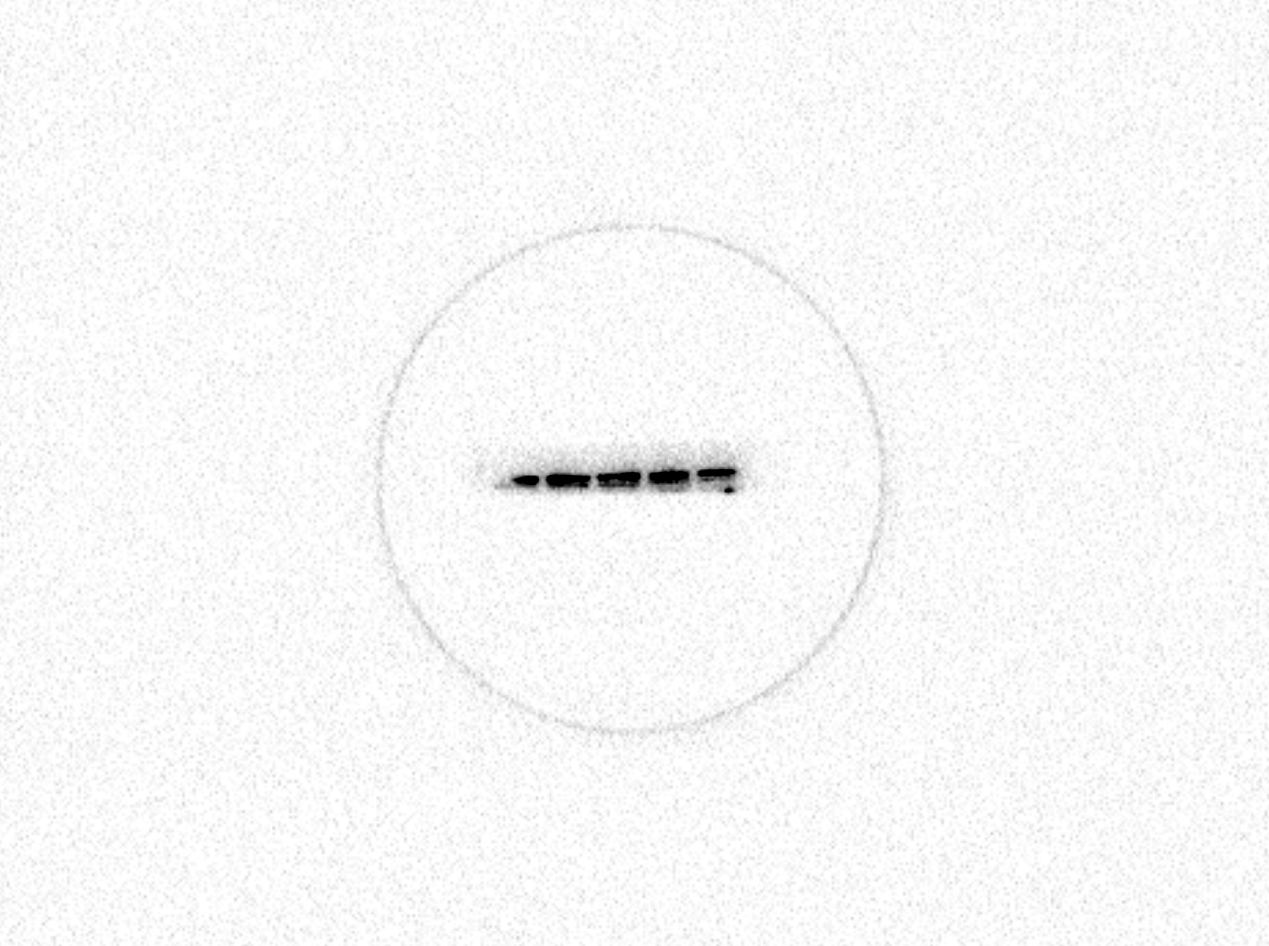


METTL14-A549

SHV

SOX2-OT#4


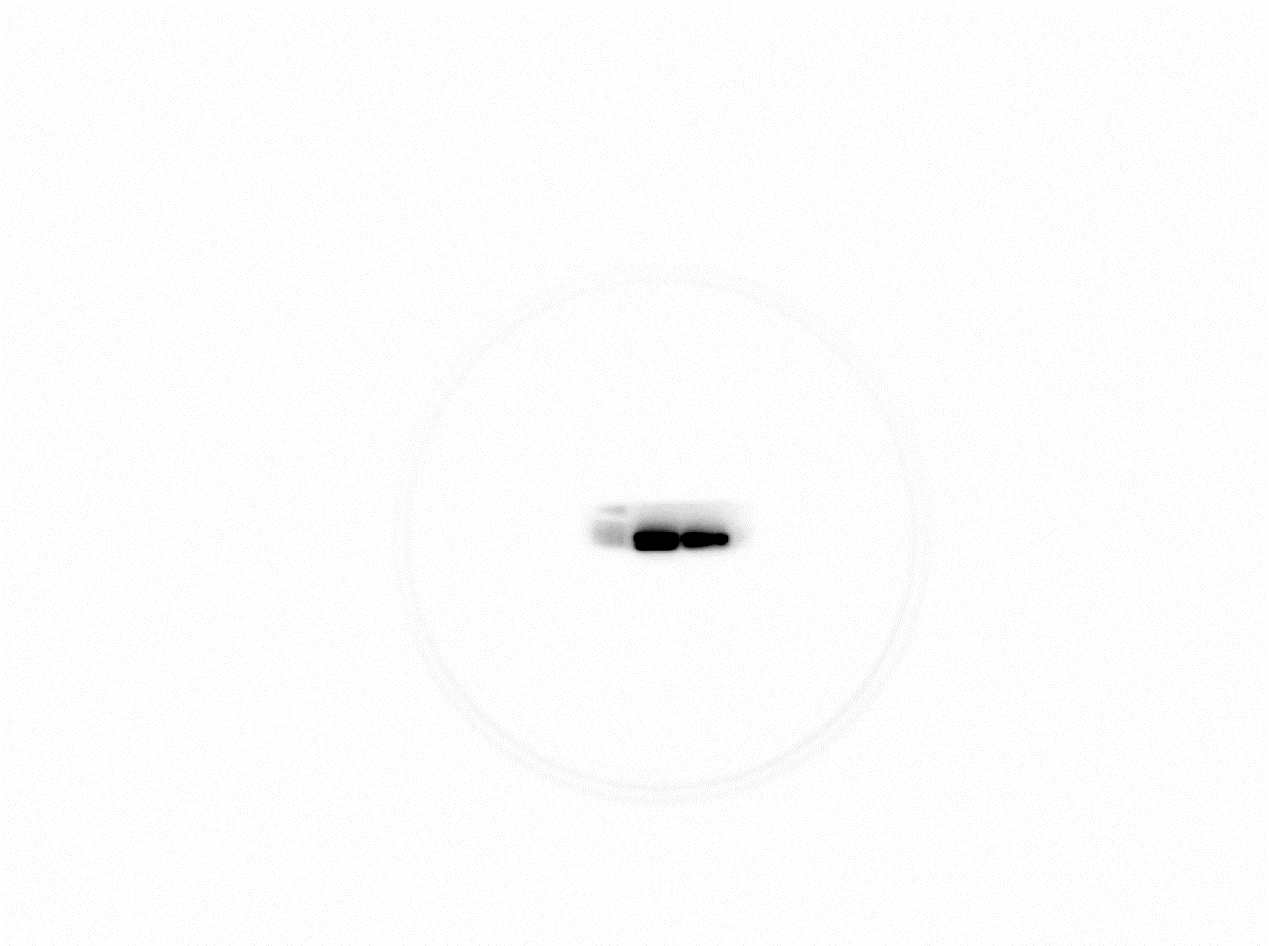


IGF2BP2-A549

SHV

SOX2-OT#4


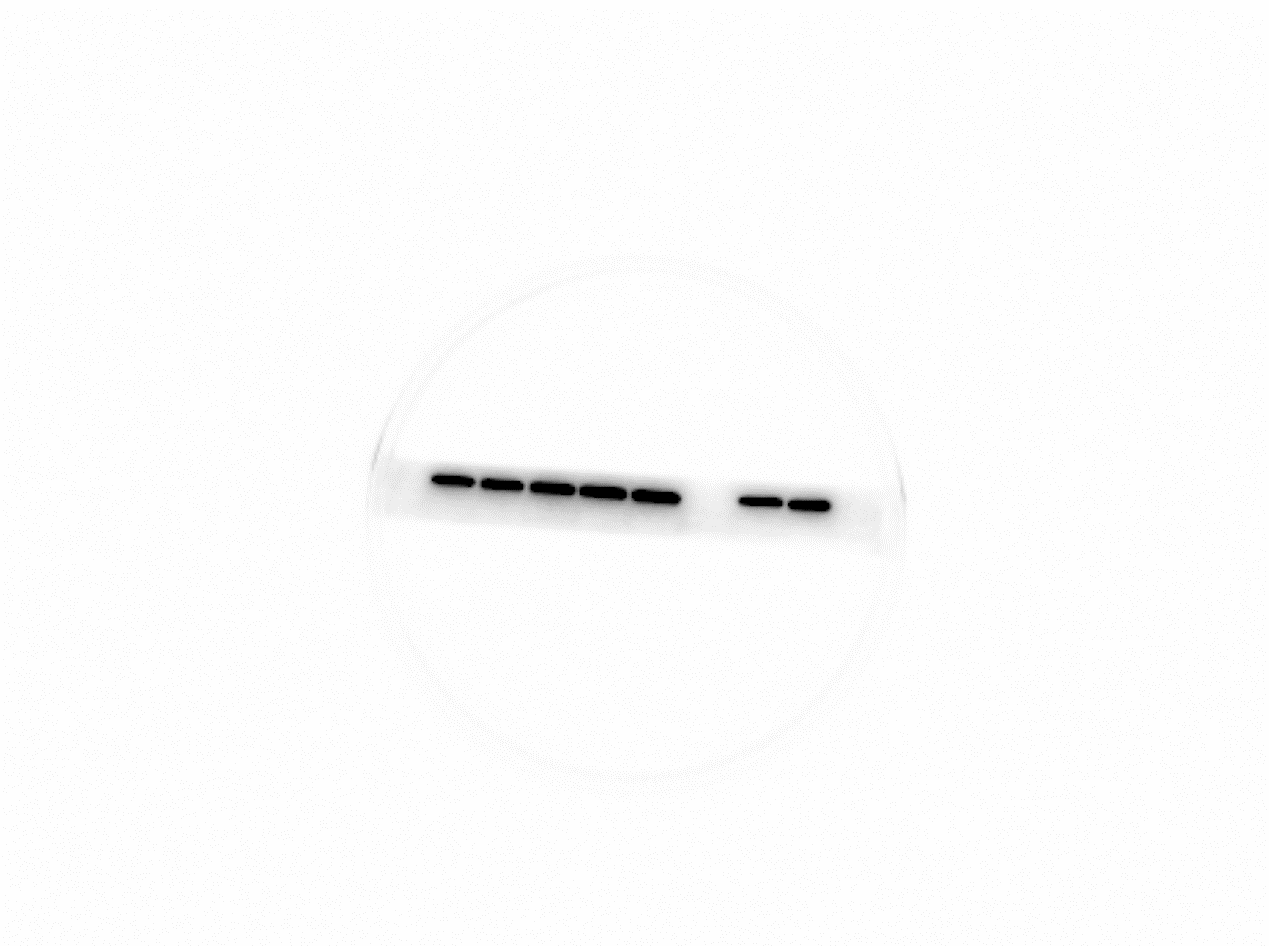


β-Actin-A549

SHV

SOX2-OT#4


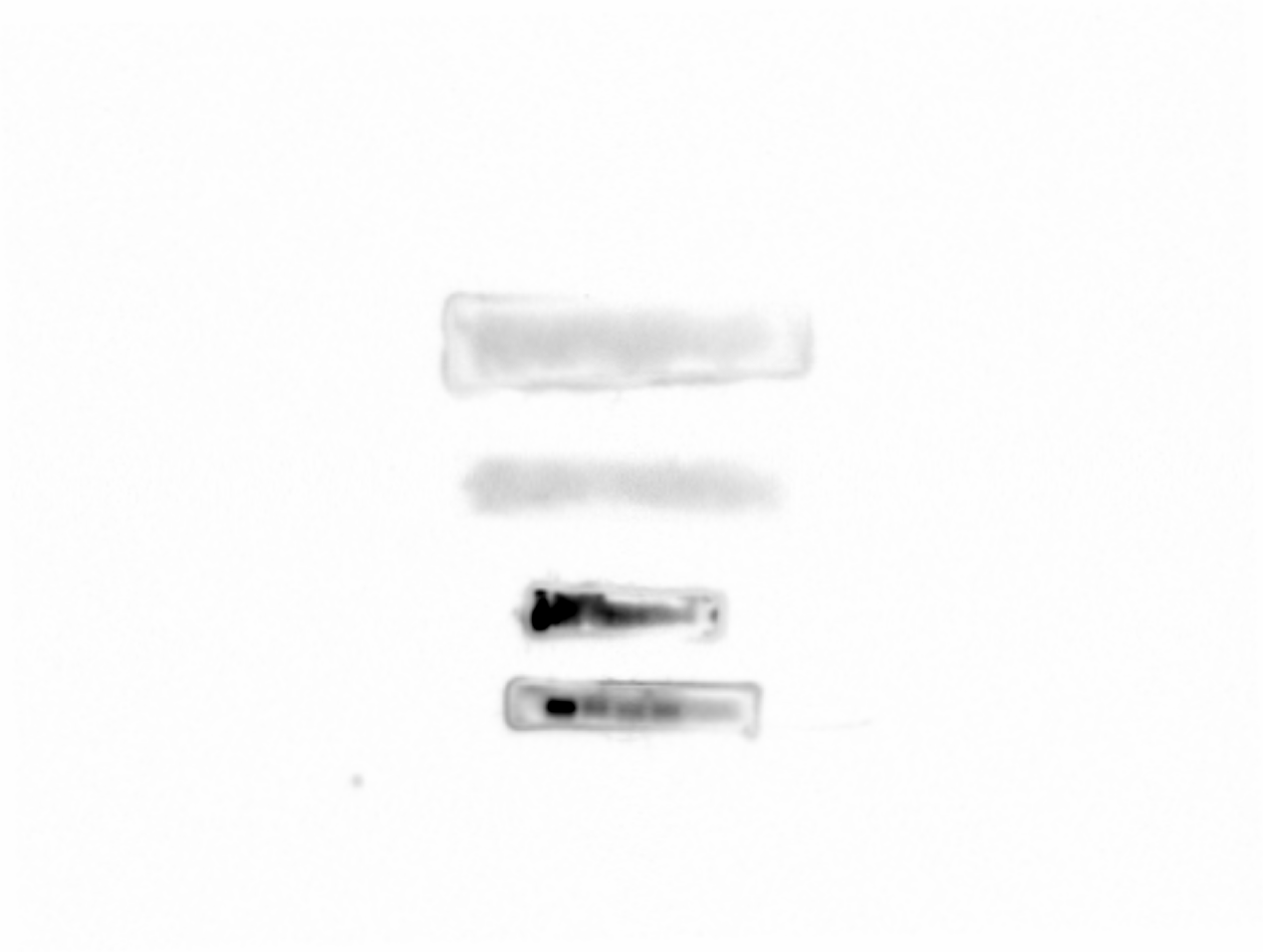


GLI1-H1299

SHV

SOX2-OT#4


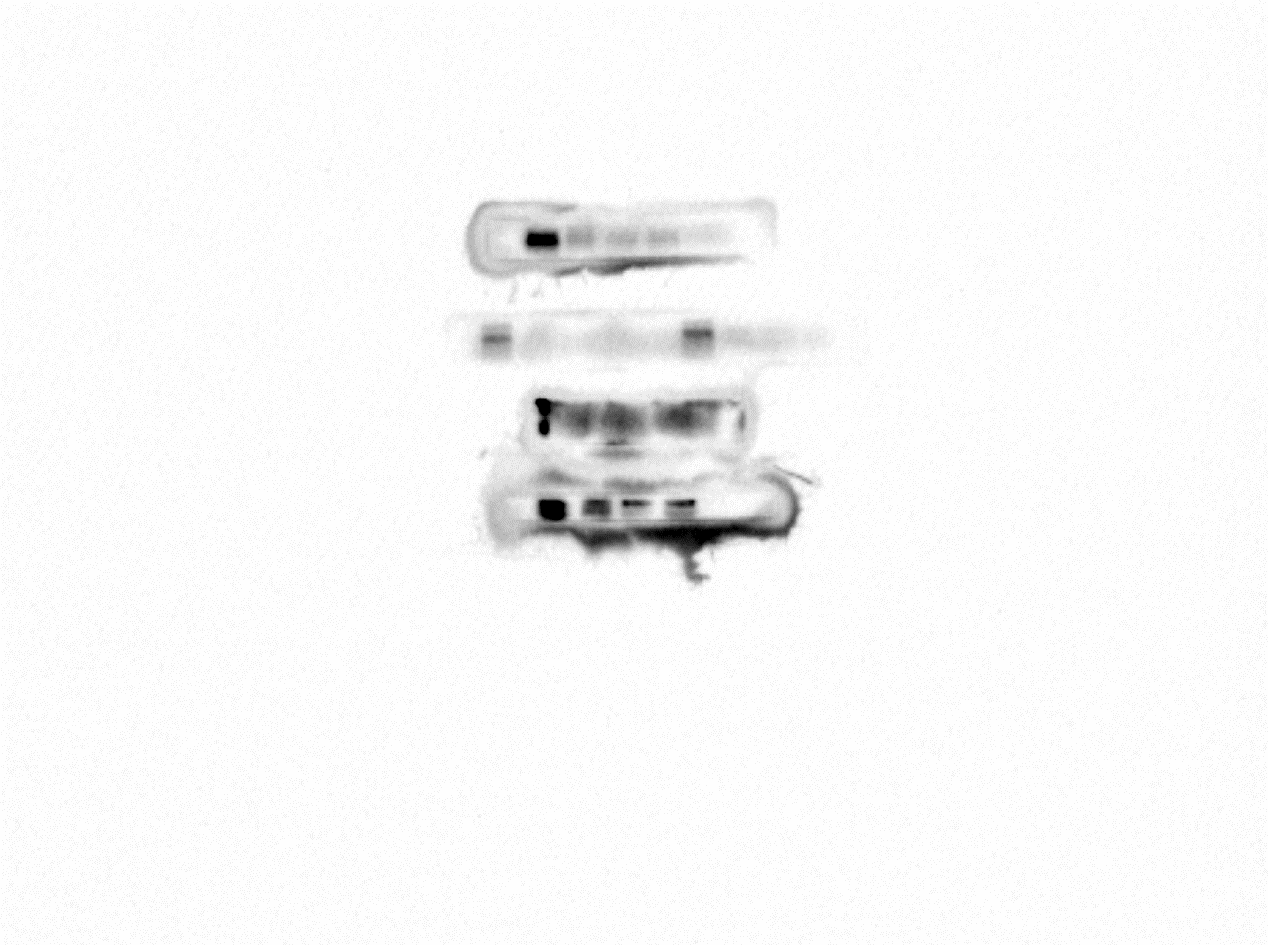


METTL3-H1299

SHV

SOX2-OT#4


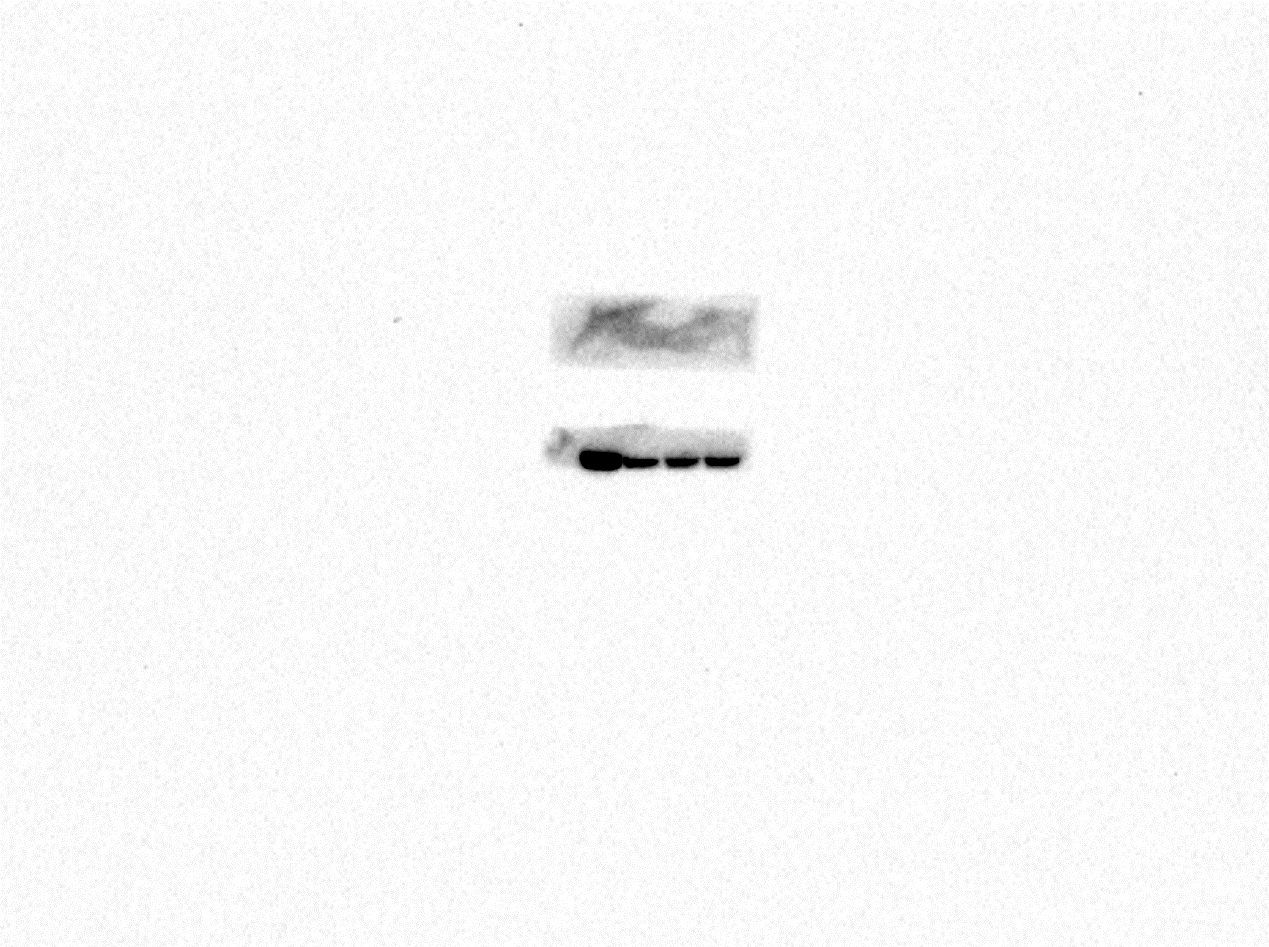


METTL14-H1299

SHV

SOX2-OT#4


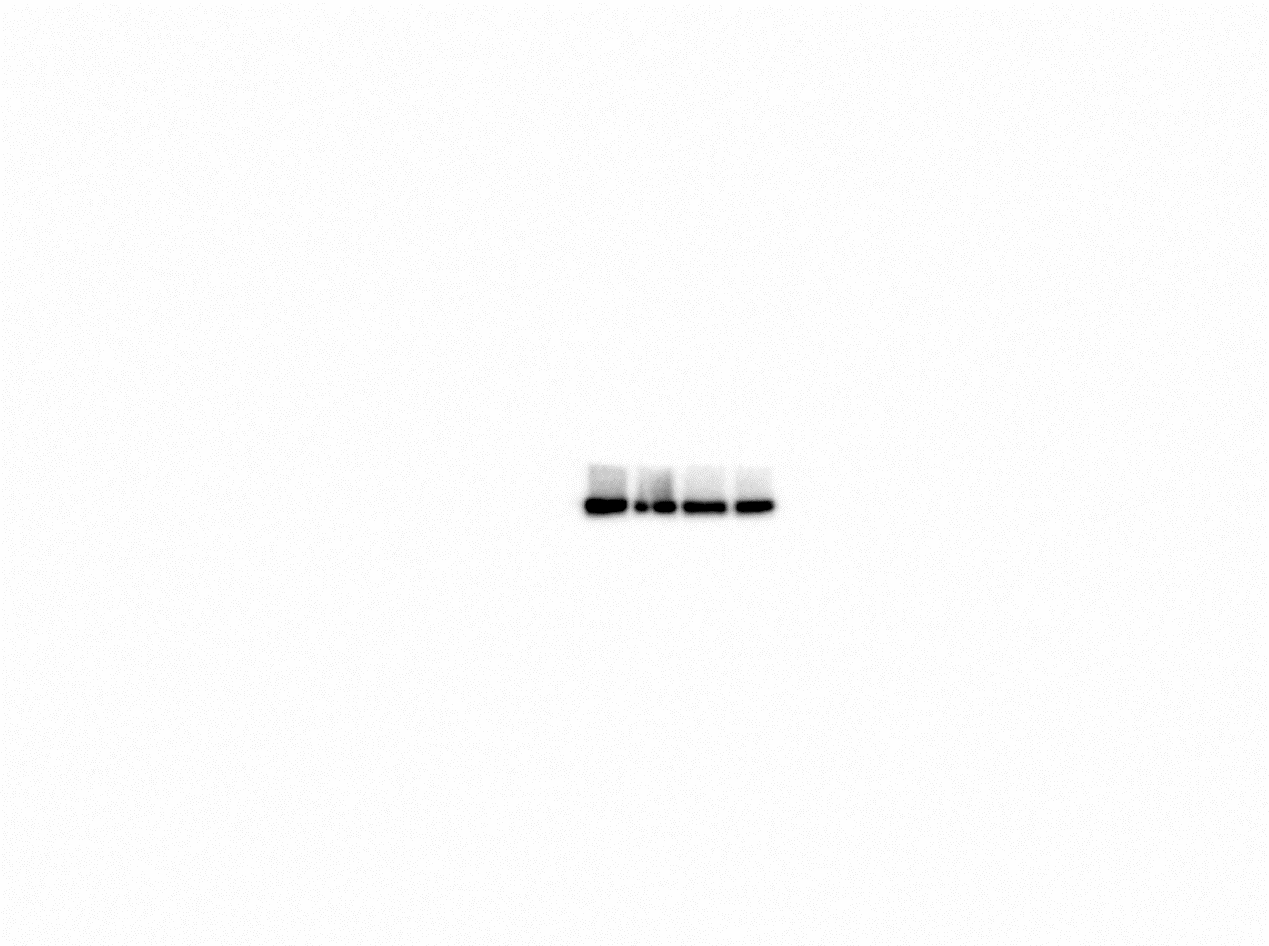


IGF2BP2-H1299

SHV

SOX2-OT#4


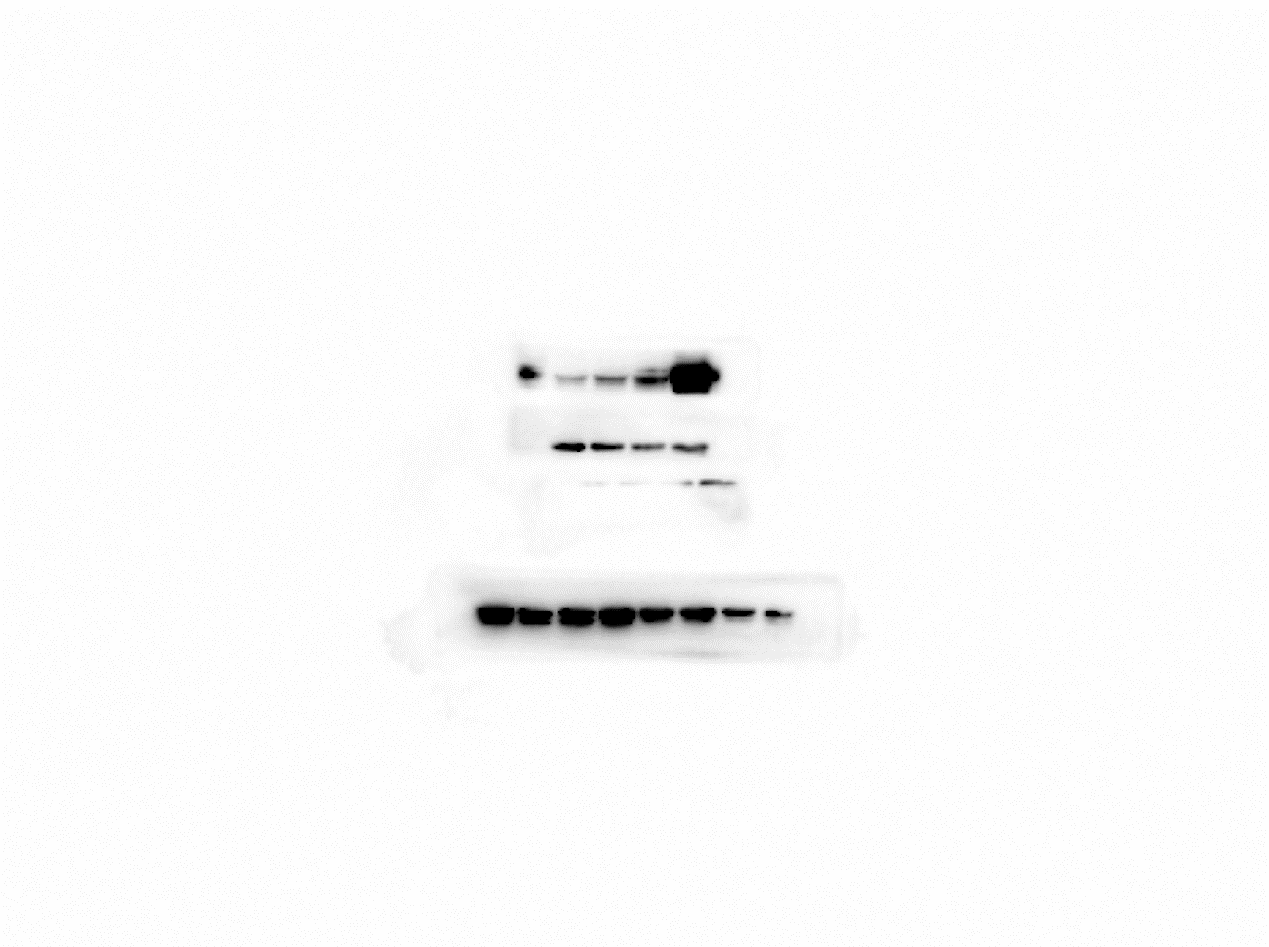


β-Actin-H1299

SHV

SOX2-OT#4

FIG6D


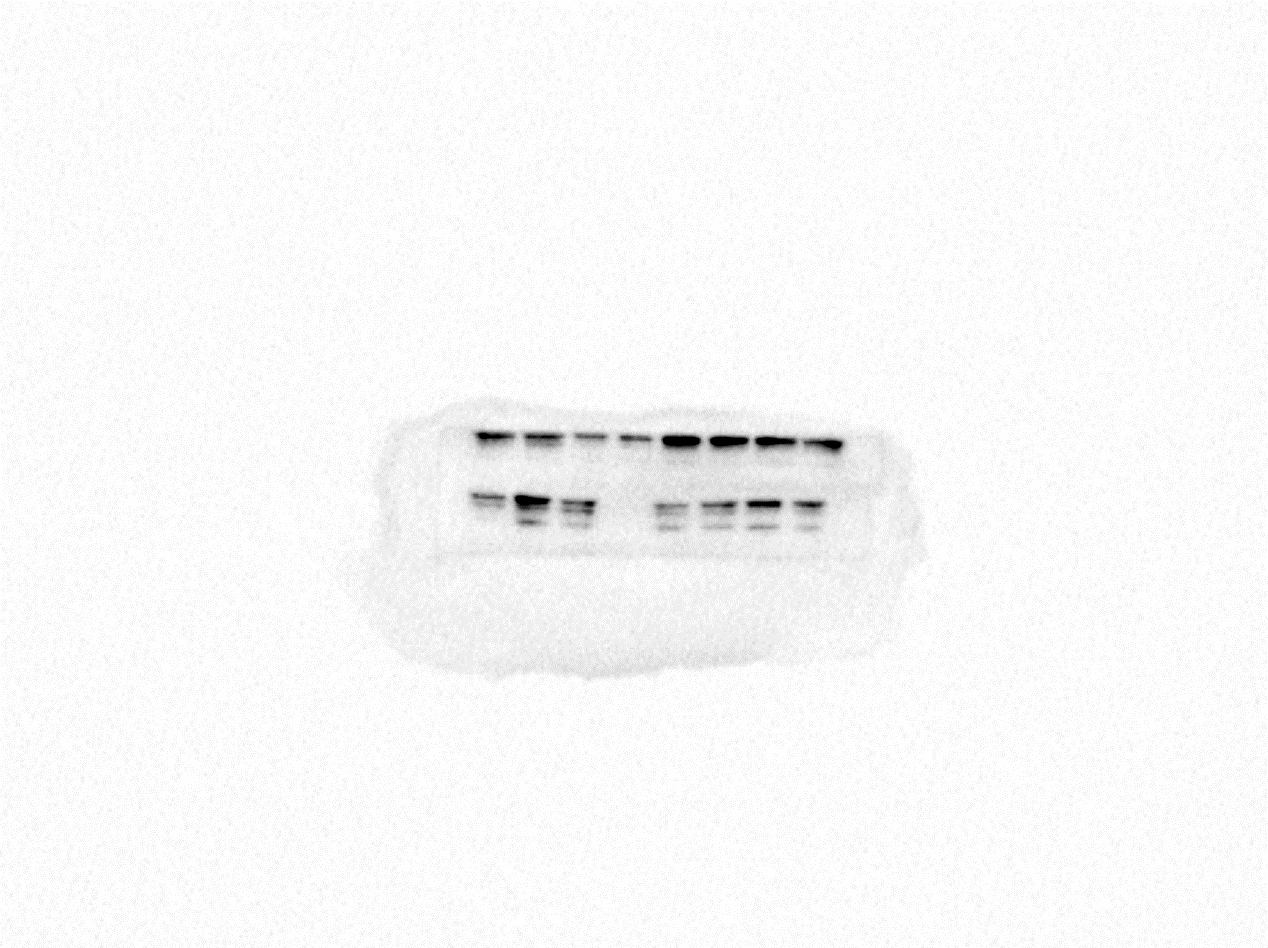


GLI1-A549

PCDNA3.1

GLI1

GLI1/#4


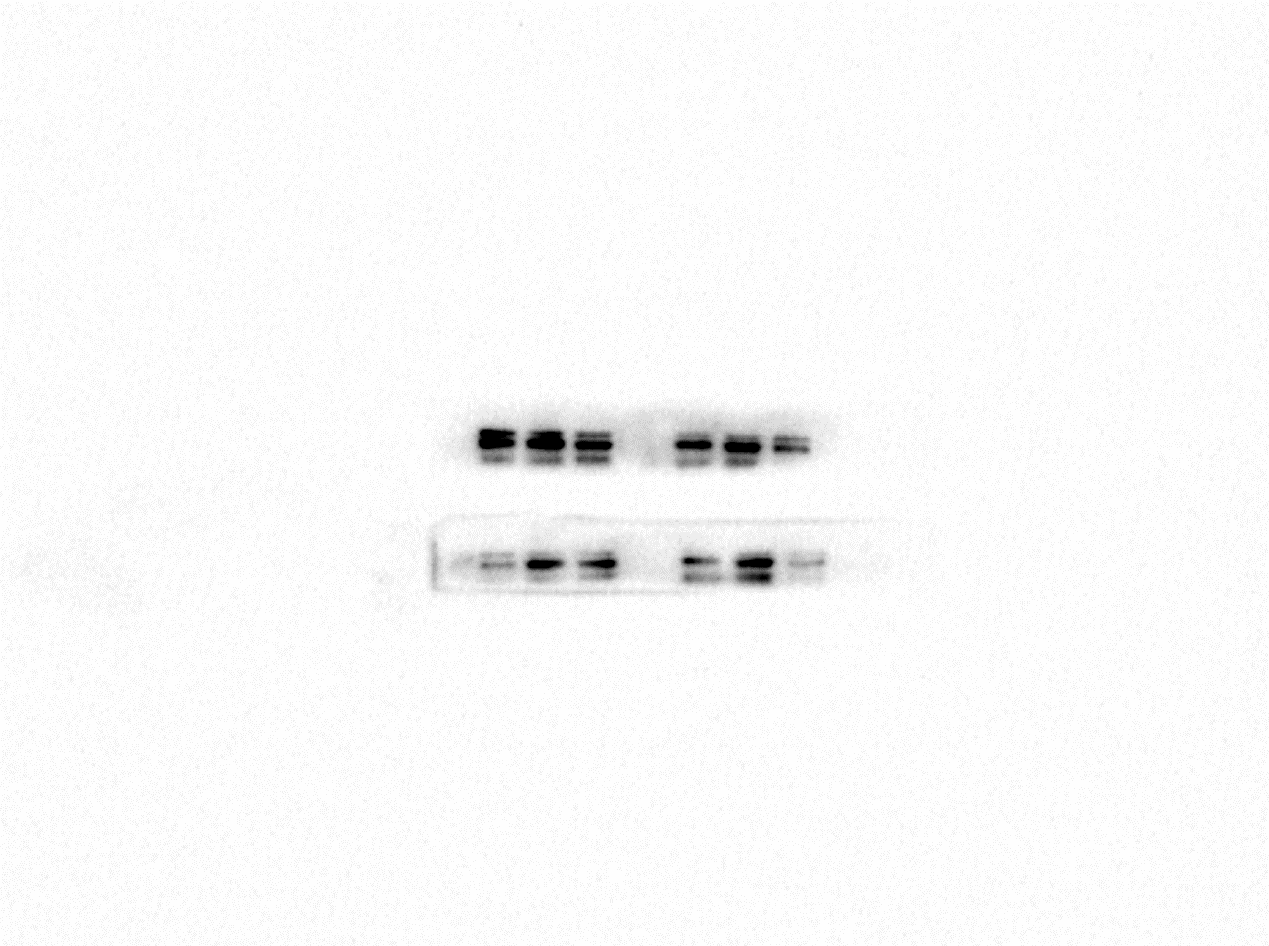


METTL3-A549

PCDNA3.1

GLI1

GLI1/#4


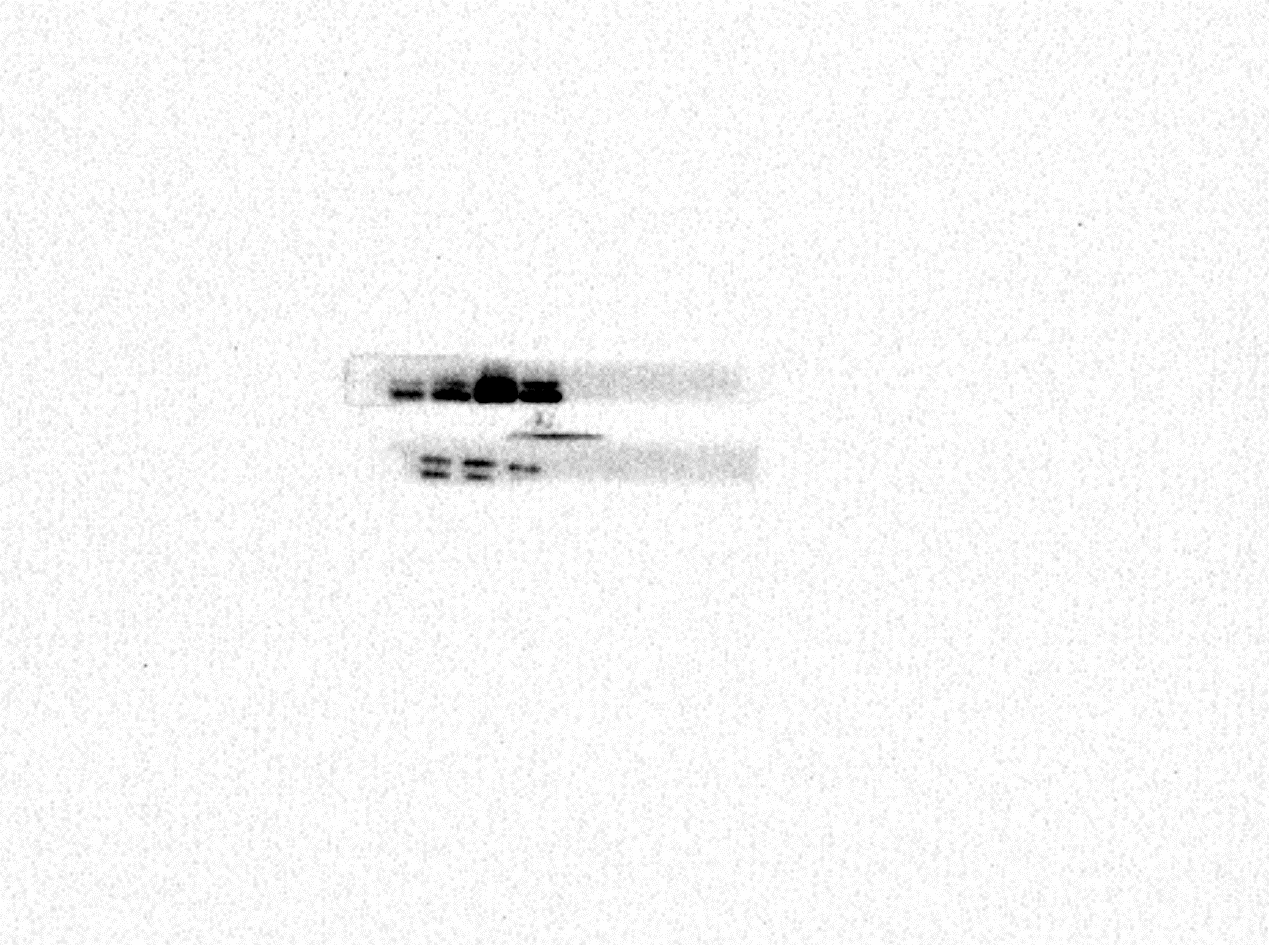


METTL14-A549

PCDNA3.1

GLI1

GLI1/#4


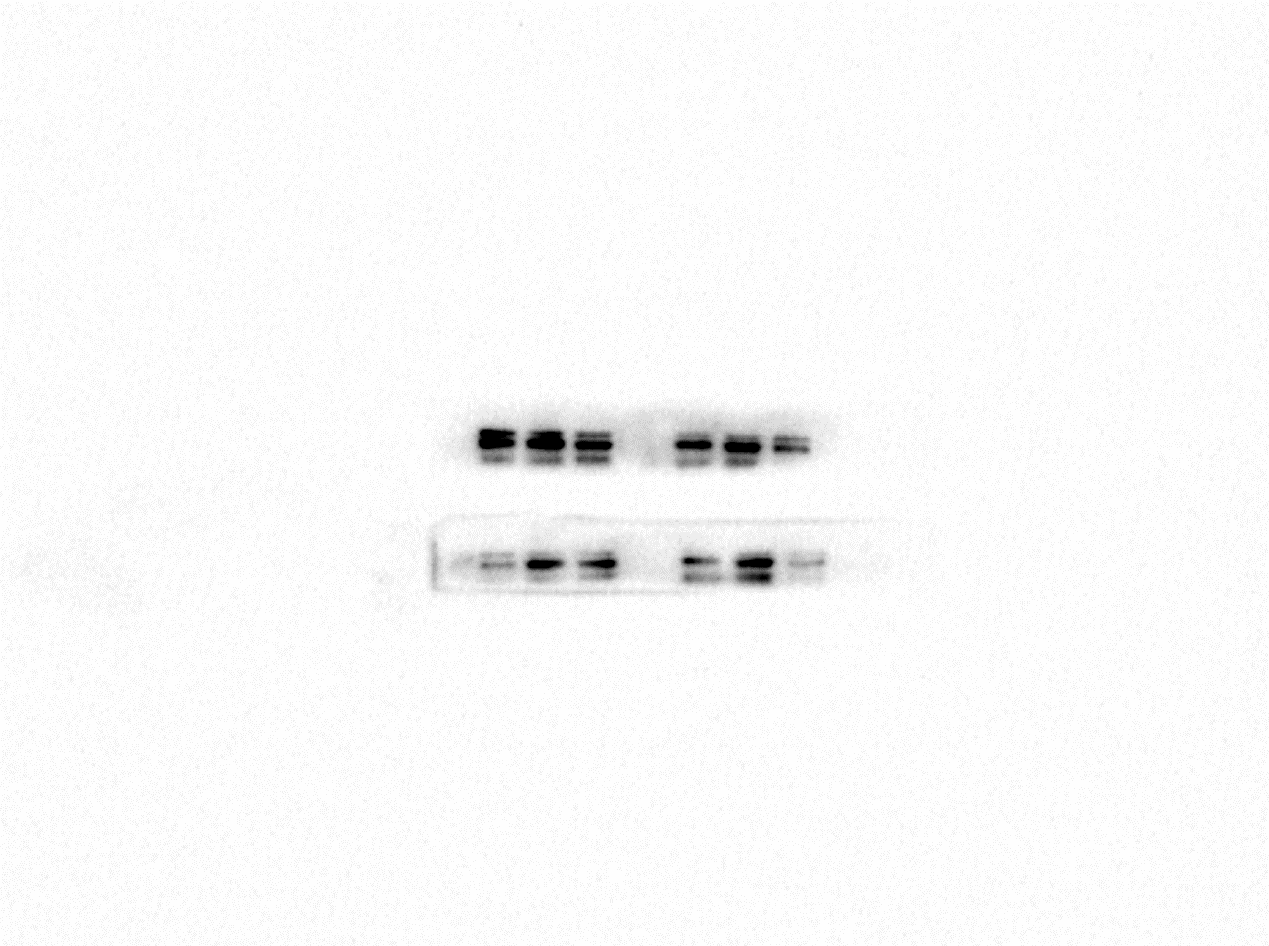


IGF2BP2-A549

PCDNA3.1

GLI1

GLI1/#4


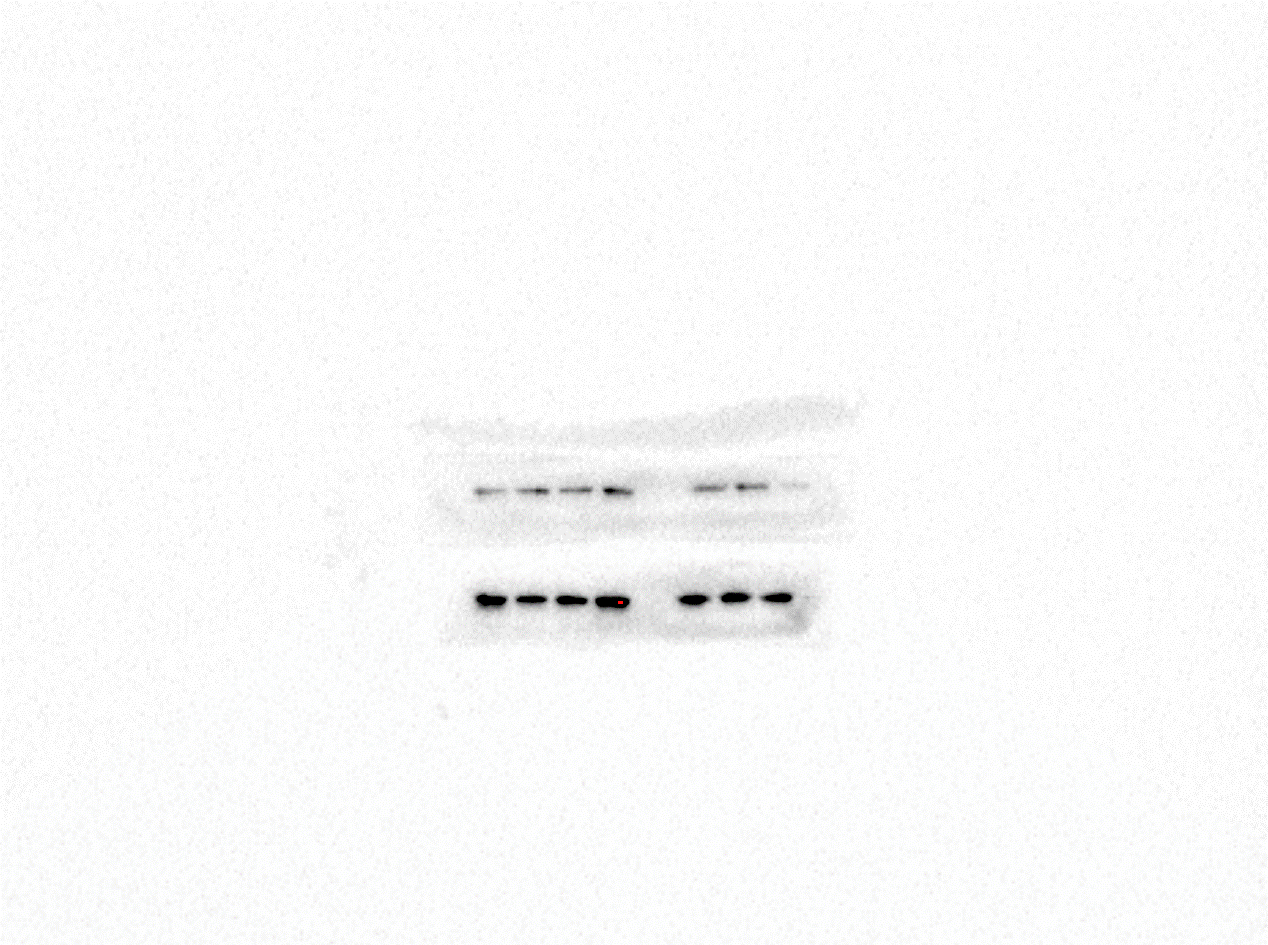


β-Actin-A549

PCDNA3.1

GLI1

GLI1/#4


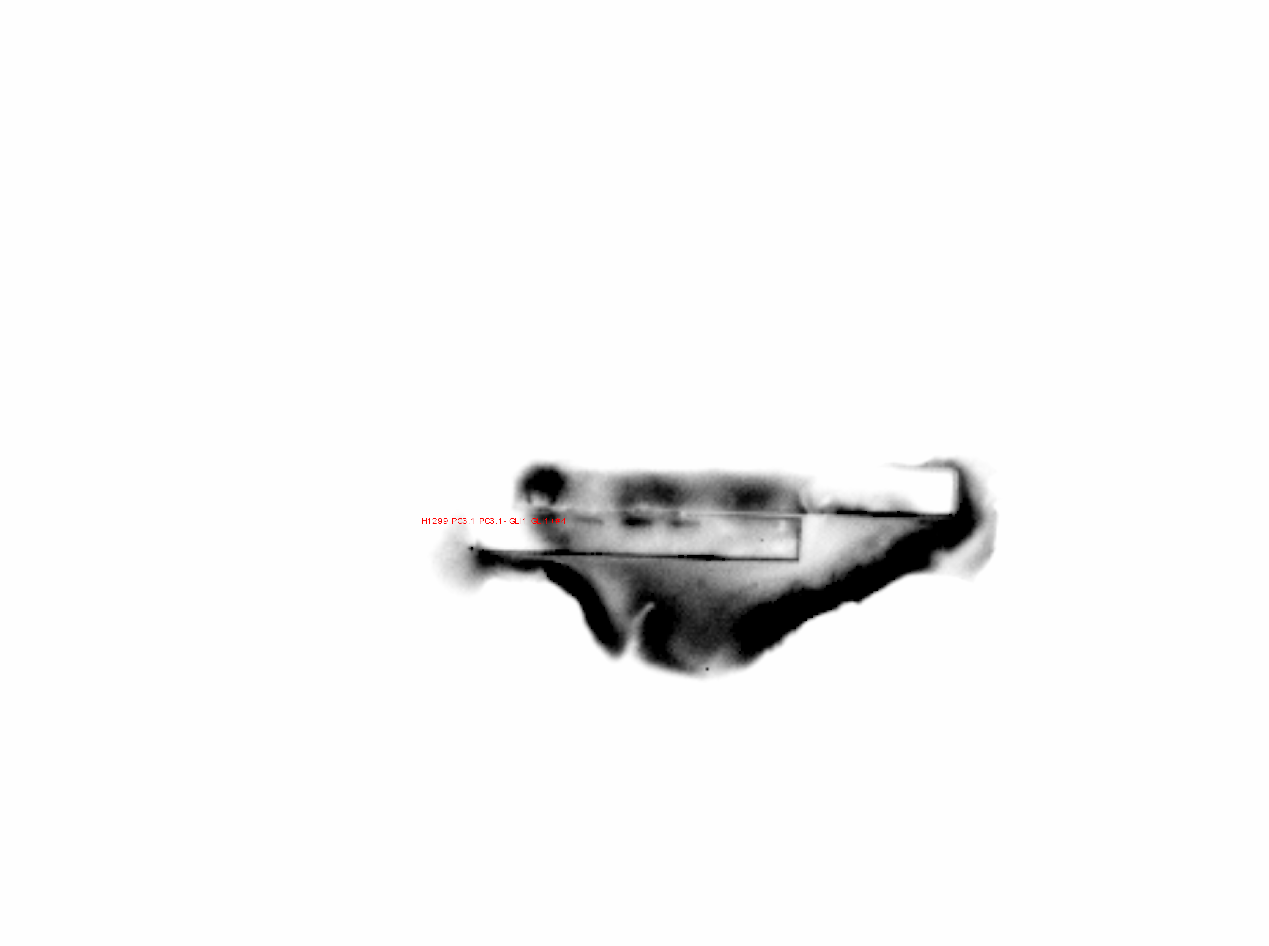


GLI1-H1299

PCDNA3.1

GLI1

GLI1/#4


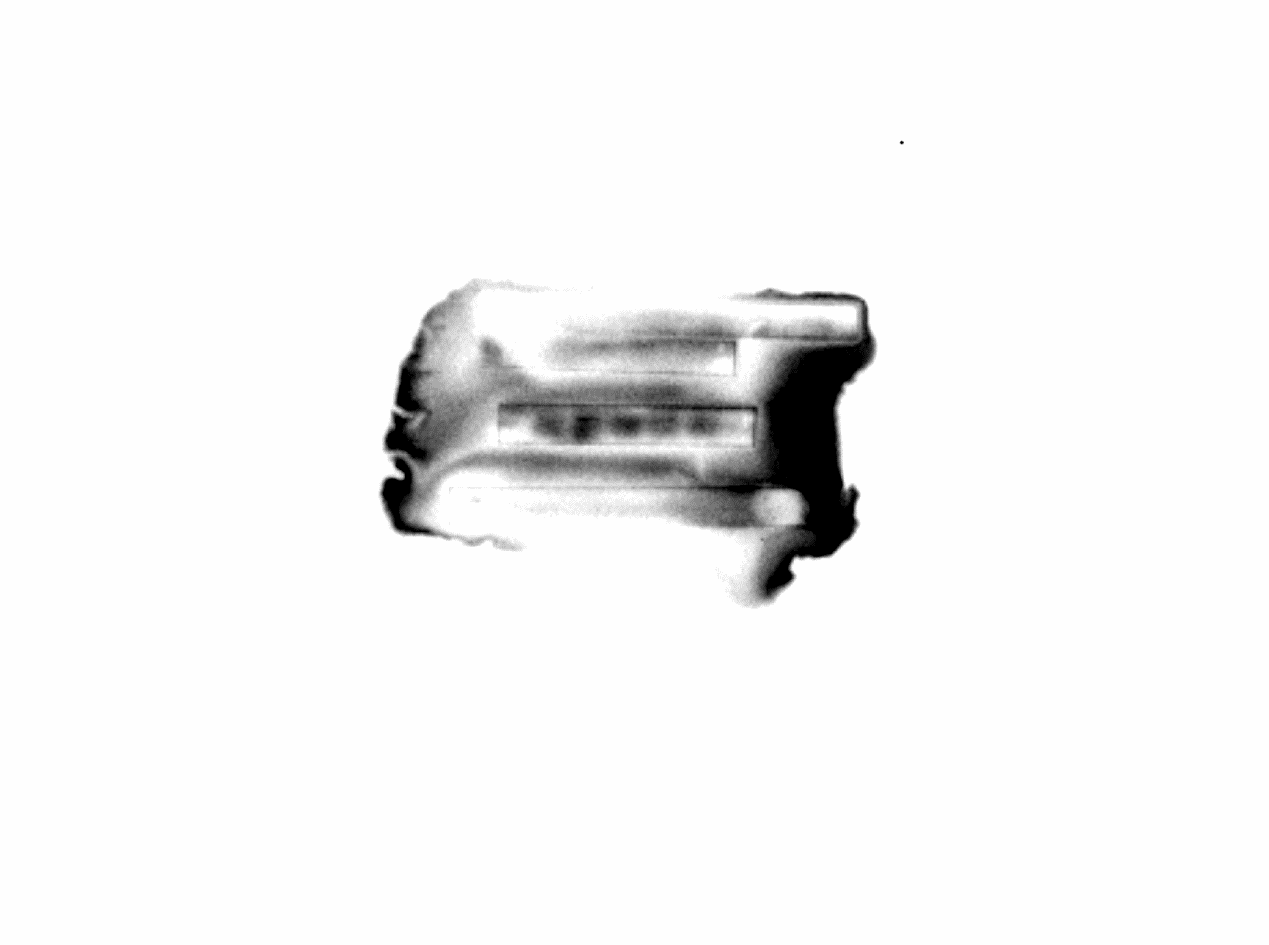


METTL13-H1299

PCDNA3.1

GLI1

GLI1/#4


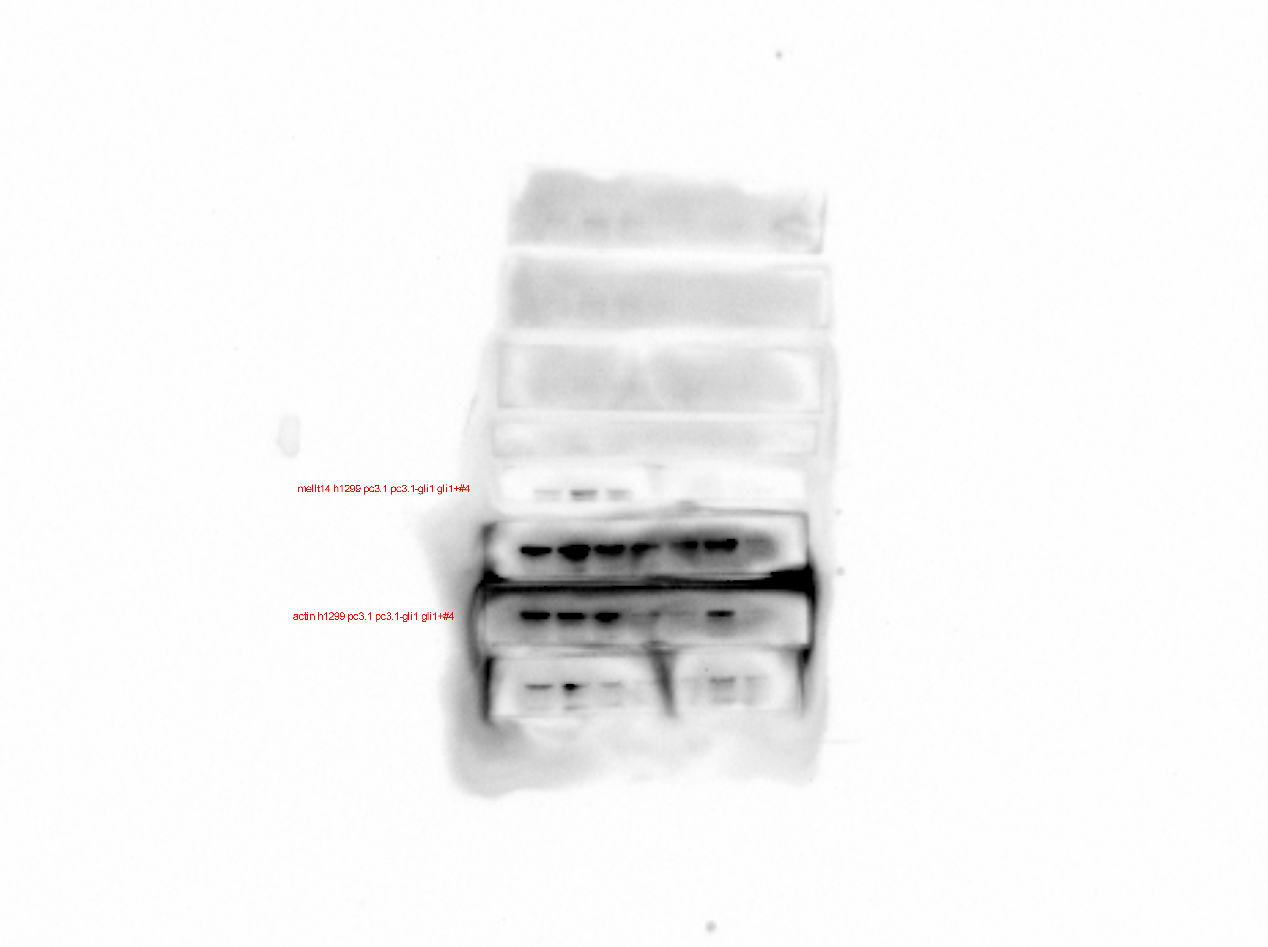


METTL14-H1299

PCDNA3.1

GLI1

GLI1/#4


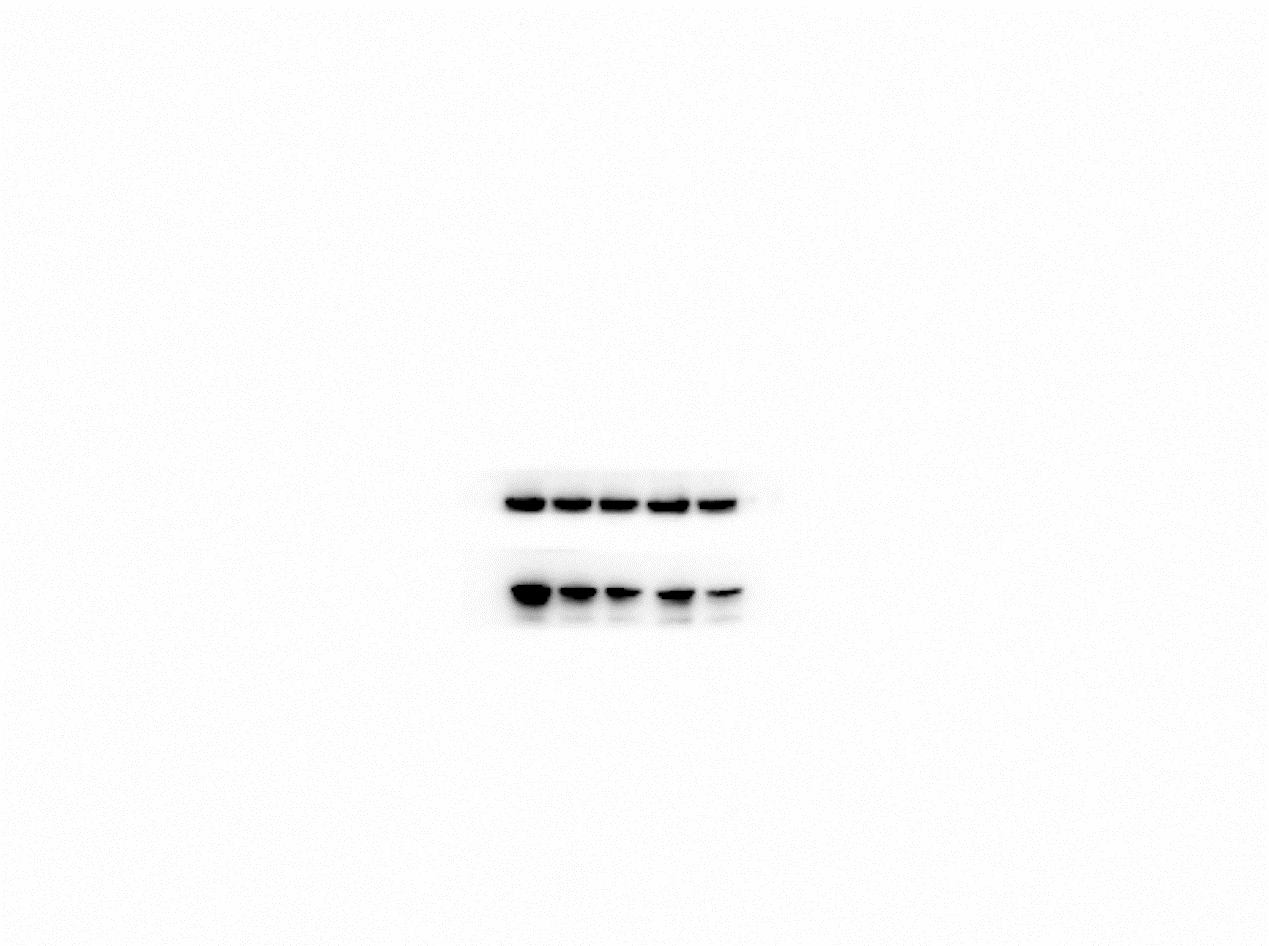


IGF2BP2-H1299

PCDNA3.1

GLI1

GLI1/#4


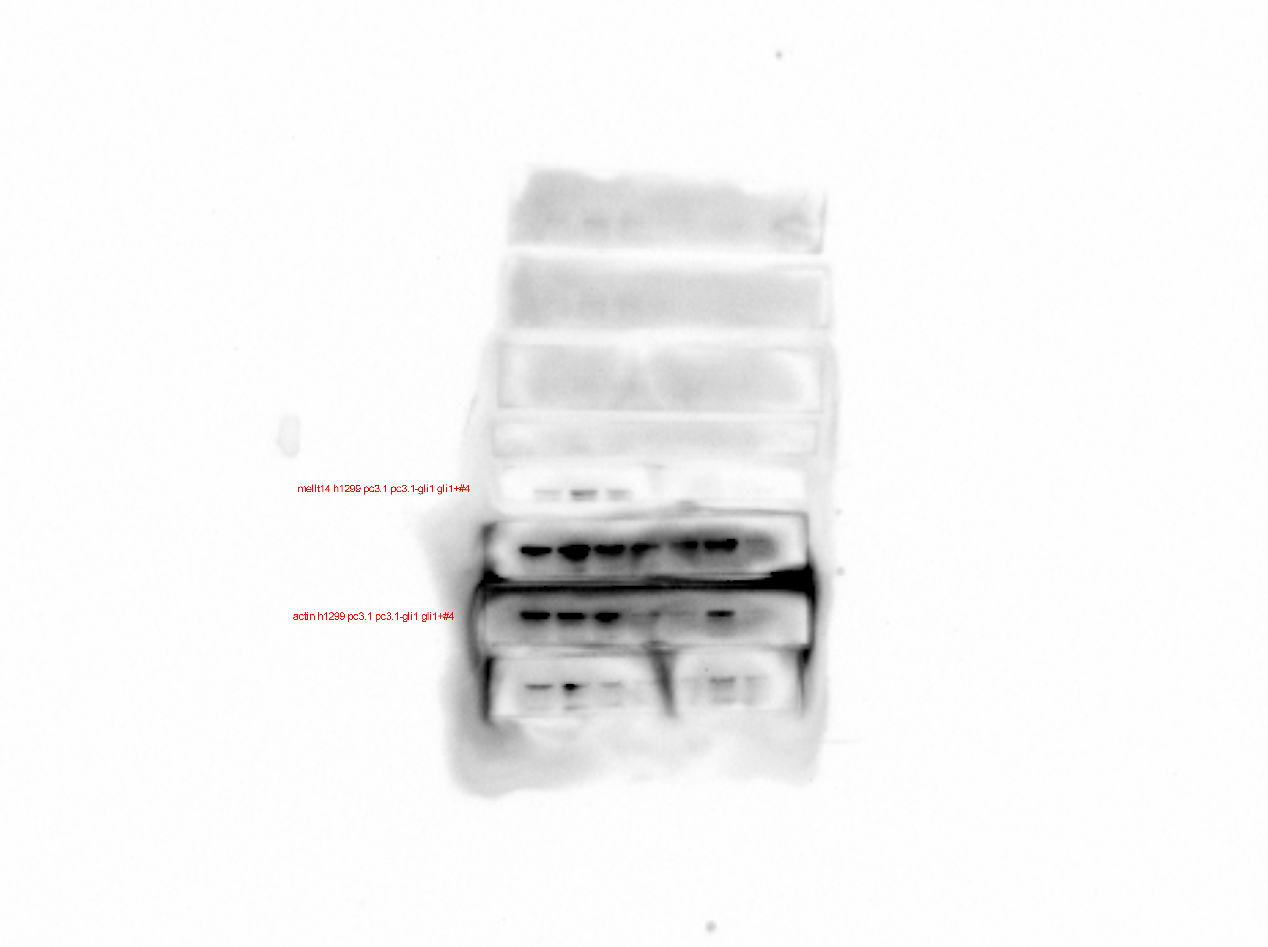


β-Actin-H1299

PCDNA3.1

GLI1

GLI1/#4

FIG6F


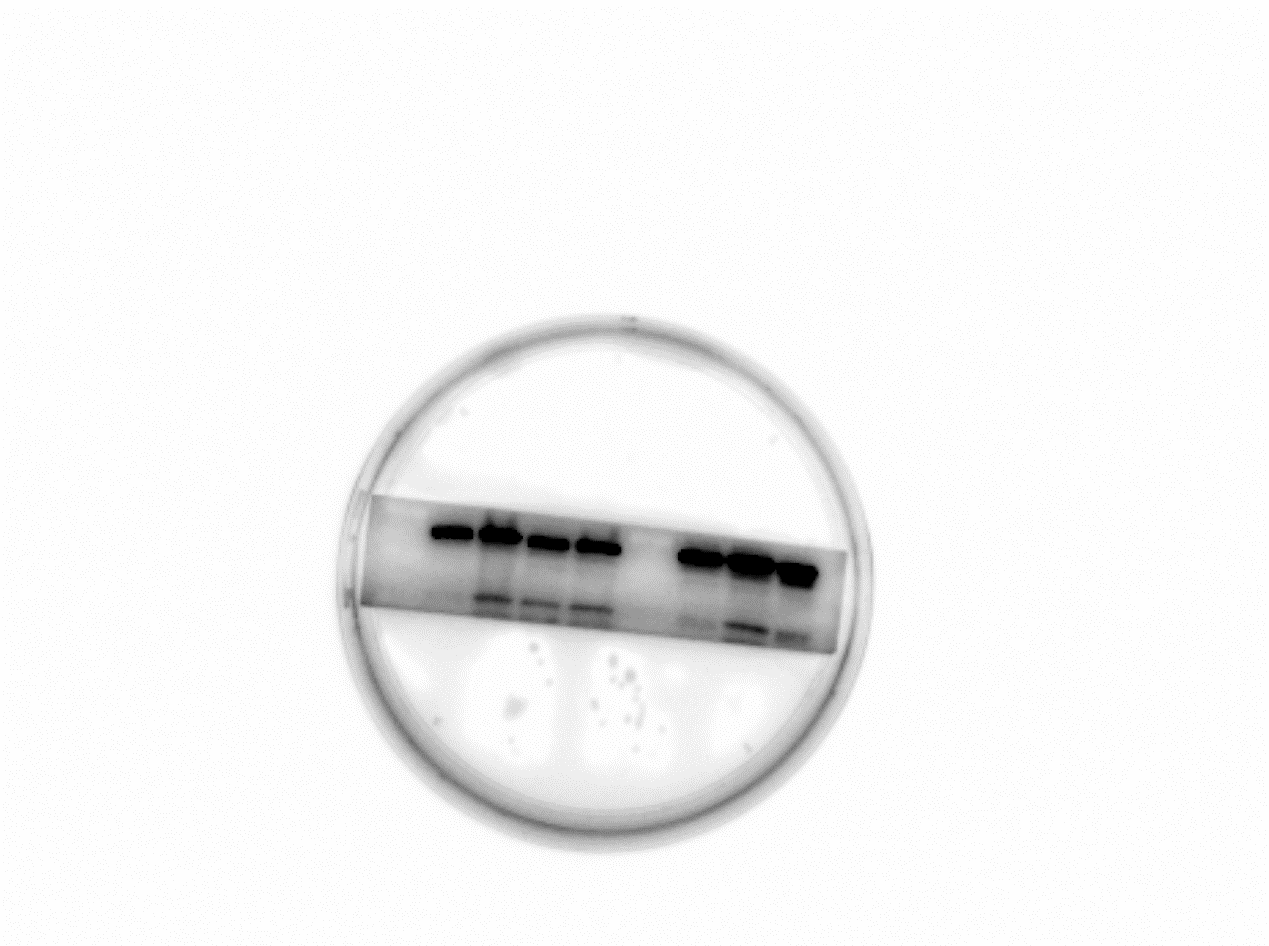


METTL3

SHV

miR185-5p inhibitor

SOX2-OT #4

SOX2-OT#4+ miR185-5p inhibitor


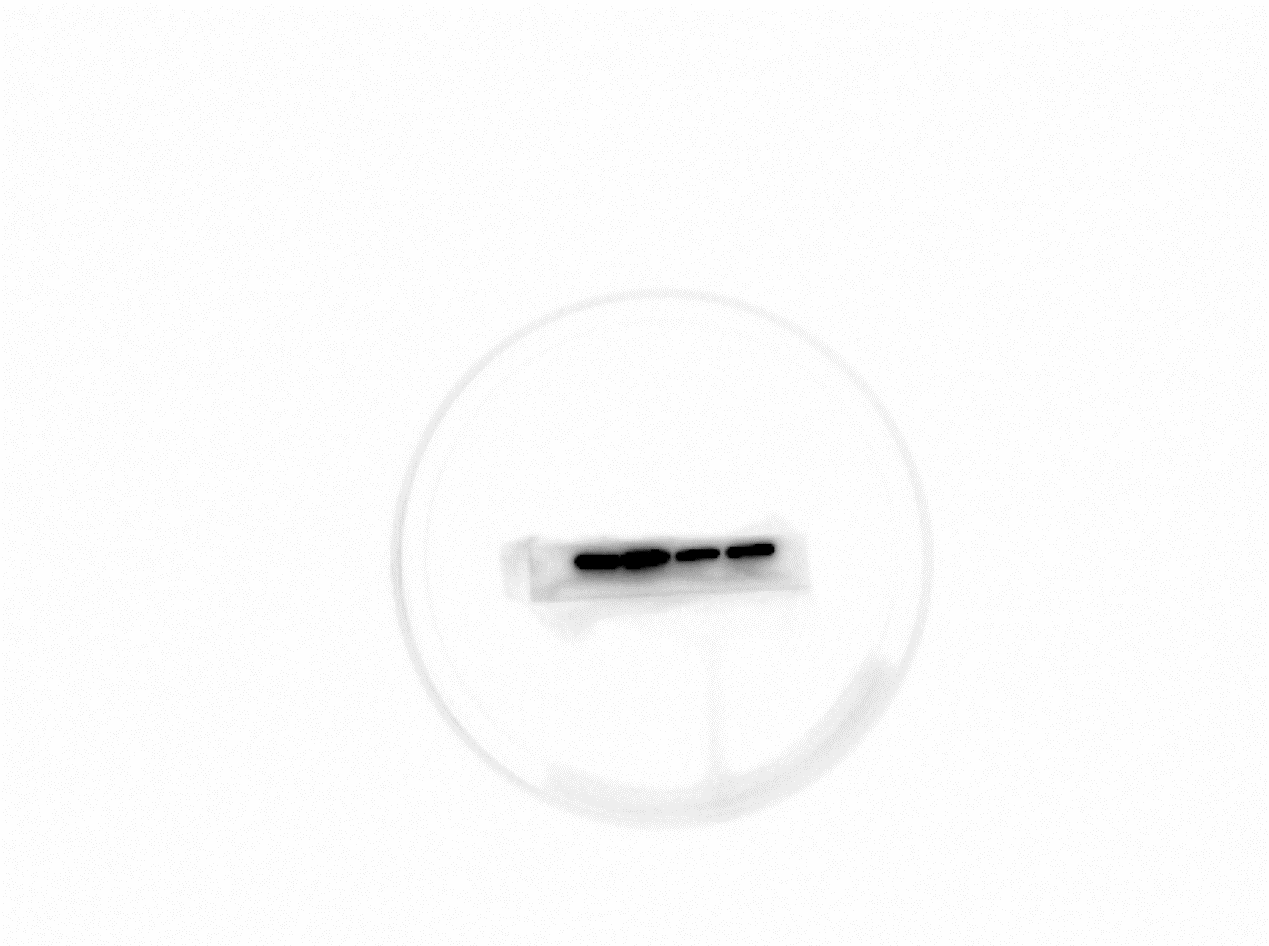


METTL14

SHV

miR185-5p inhibitor

SOX2-OT #4

SOX2-OT#4+ miR185-5p inhibitor

SHV

miR185-5p inhibitor

SOX2-OT #4

SOX2-OT#4+ miR185-5p inhibitor


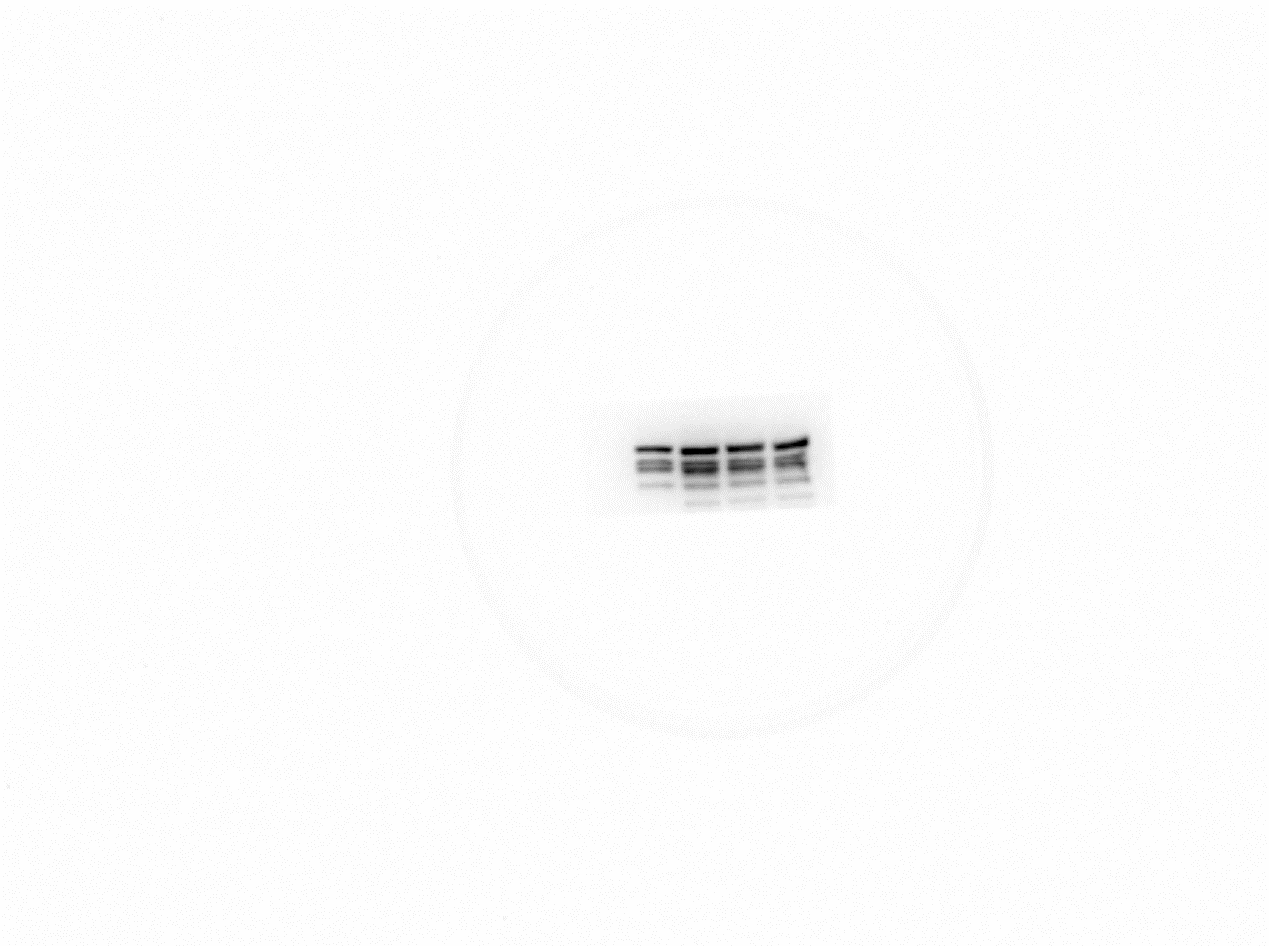


IGF2BP2

SHV

miR185-5p inhibitor

SOX2-OT #4

SOX2-OT#4+ miR185-5p inhibitor


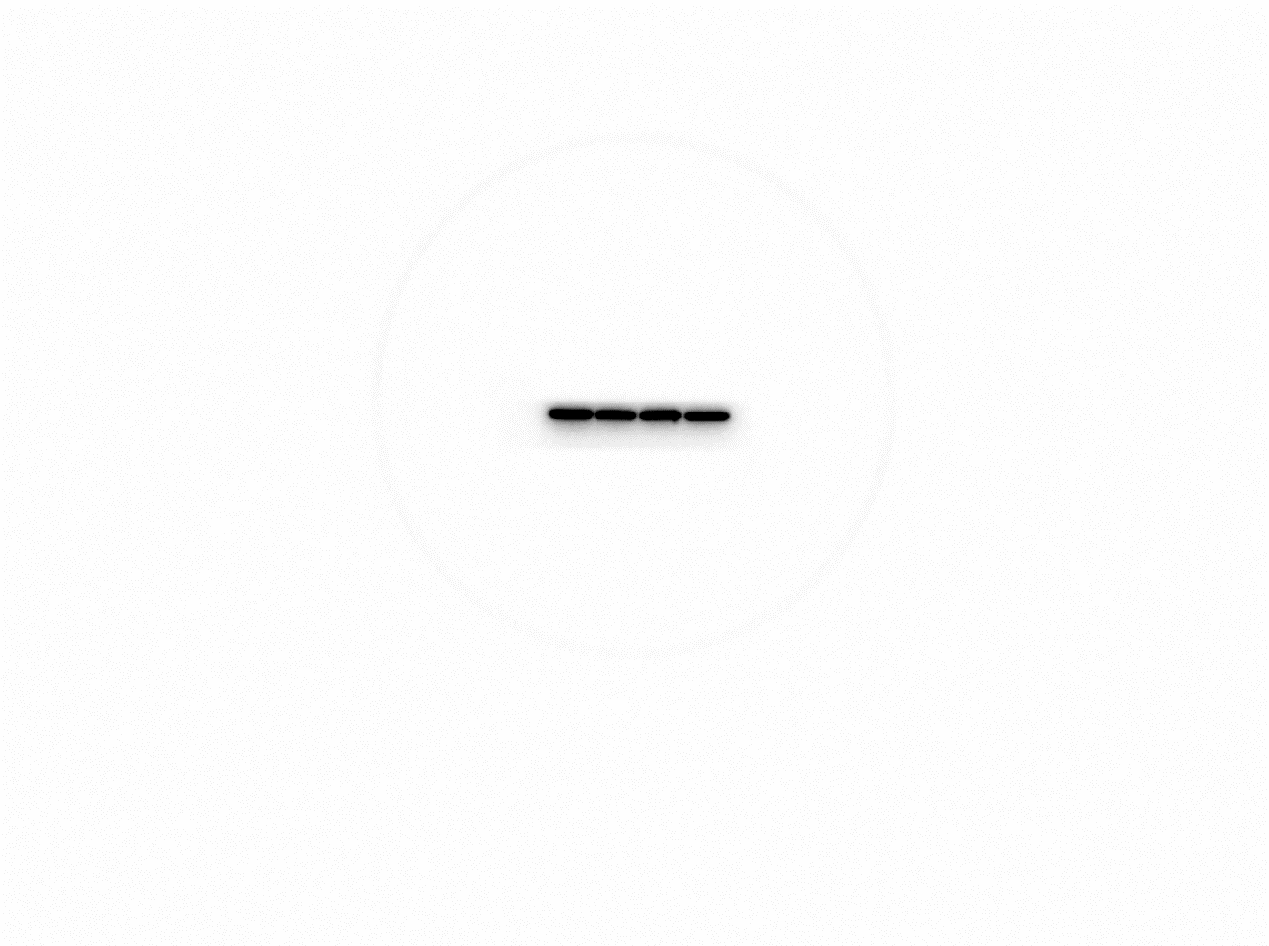


β-Actin

S4A


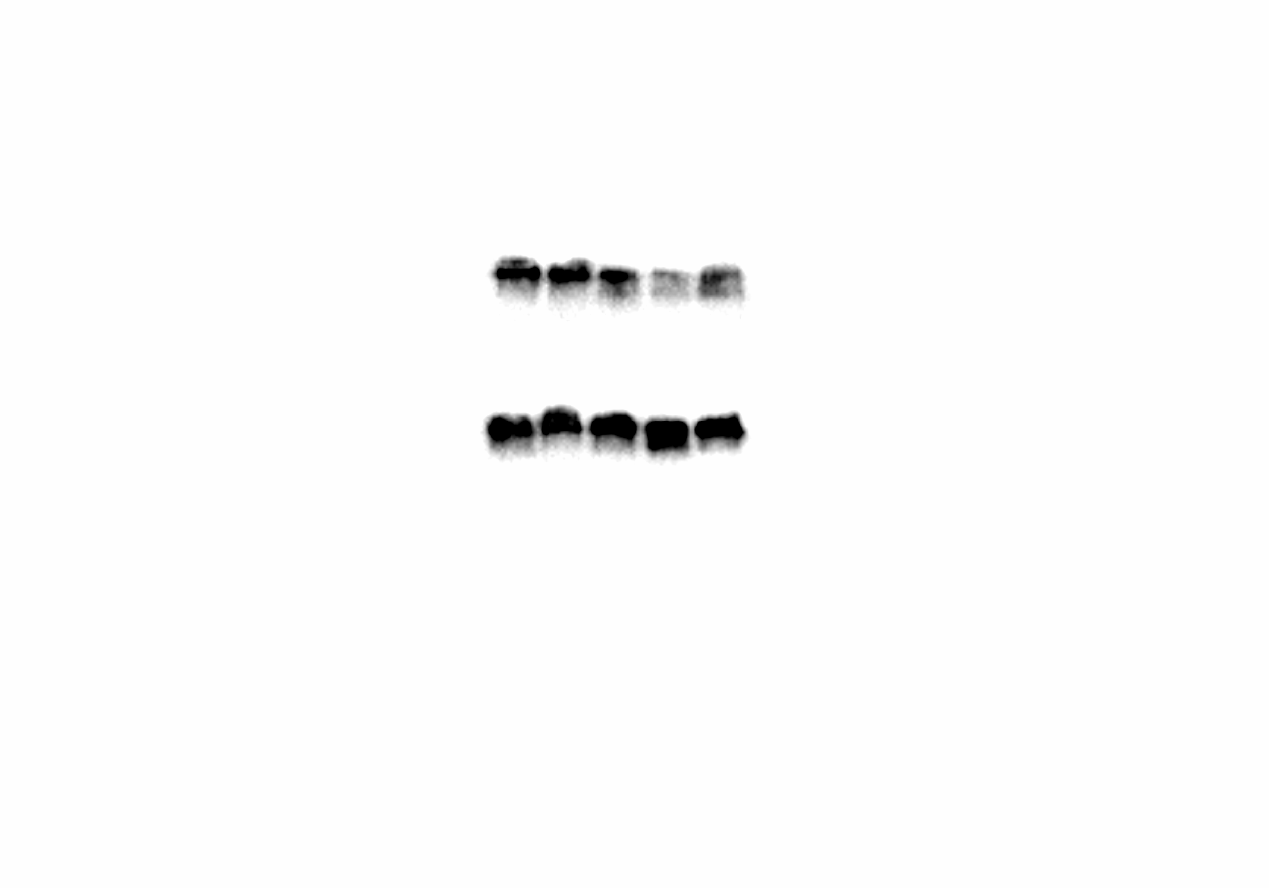


GLI1-pENTER

0h 2h 4h 6h 8h


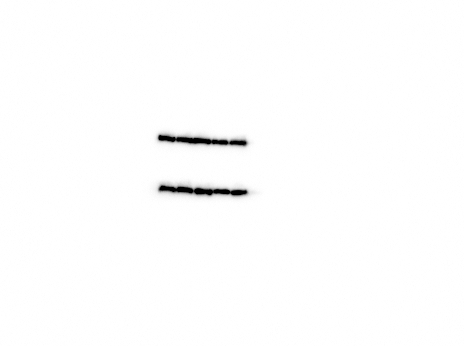


0h 2h 4h 6h 8h

α-Tubulin-pENTER


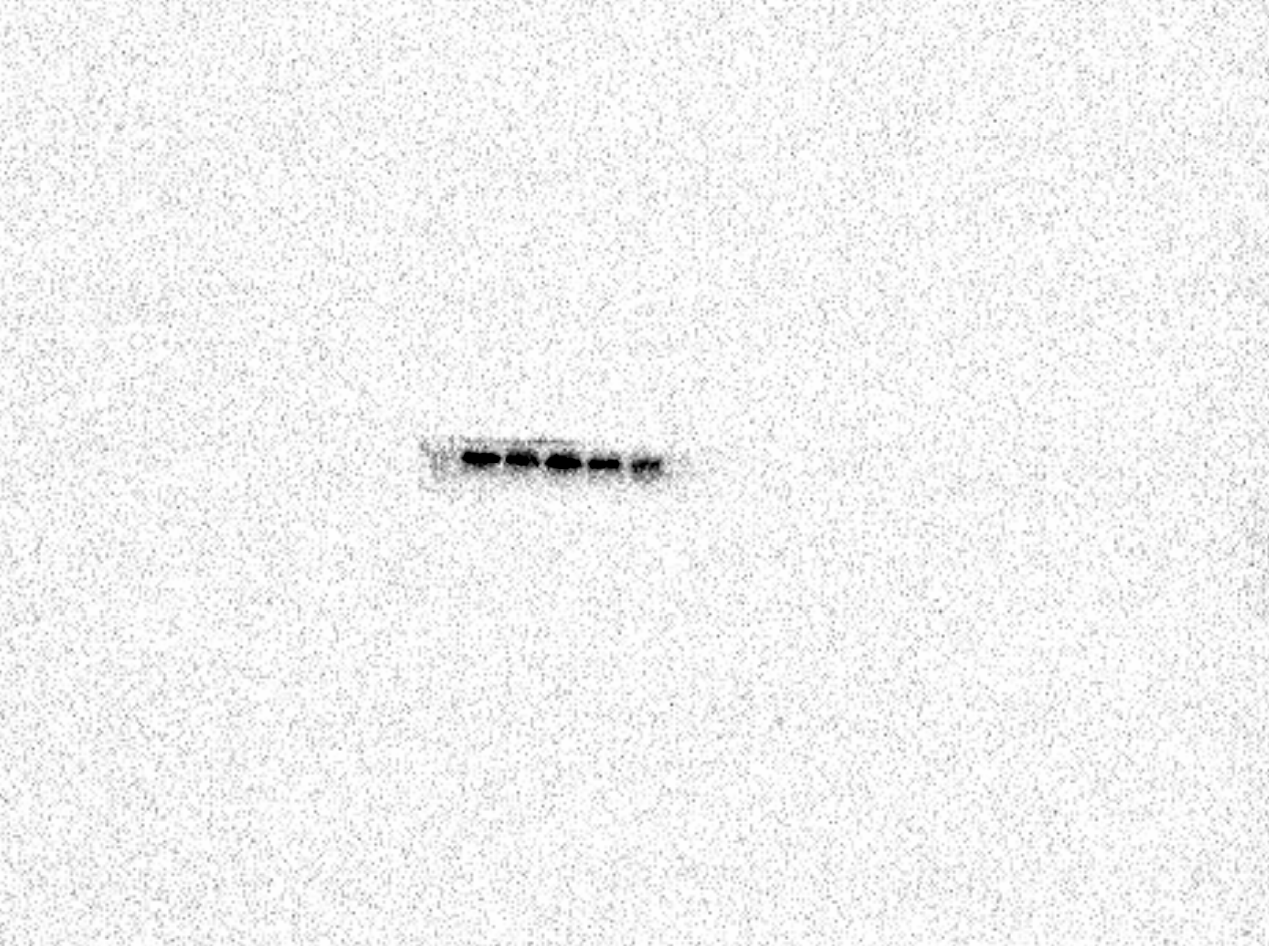


0h 2h 4h 6h 8h

GLI1-METTL3 OE


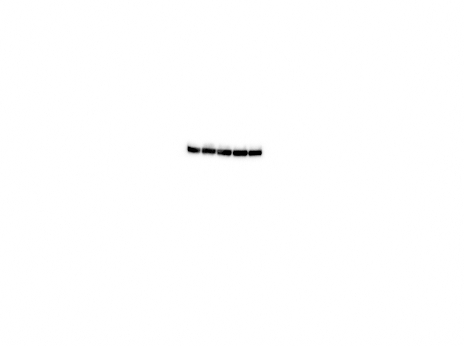


α-Tublin-METTL3 OE

0h 2h 4h 6h 8h


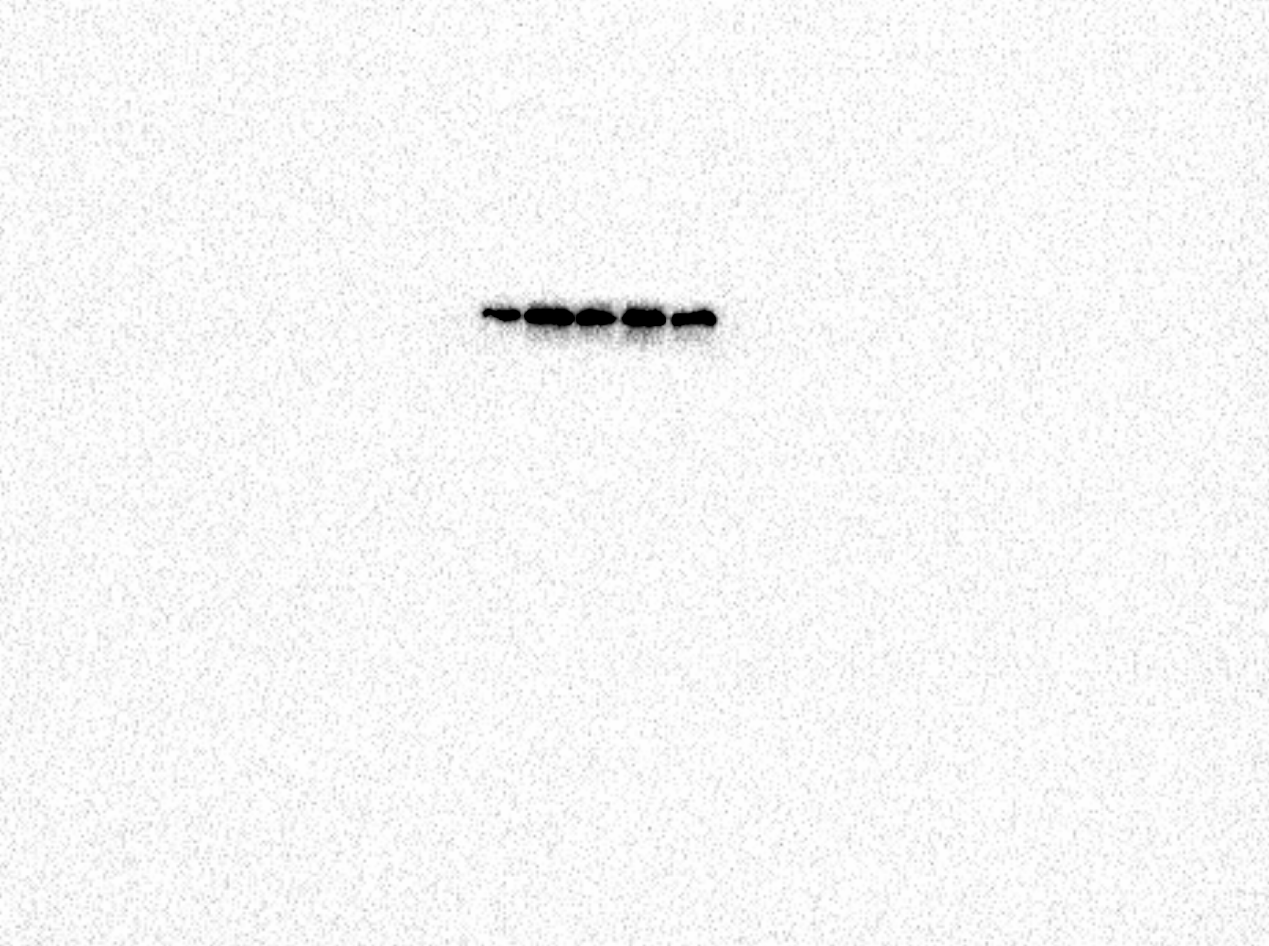


0h 2h 4h 6h 8h

GLI1-METTL14 OE


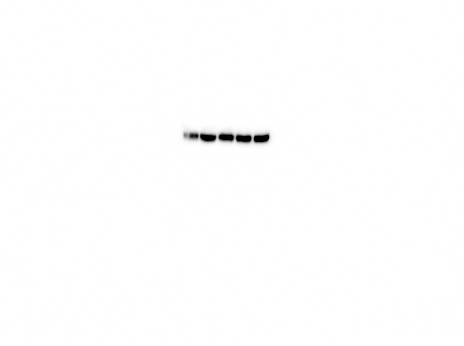


α-Tublin-METTL14 OE

0h 2h 4h 6h 8h


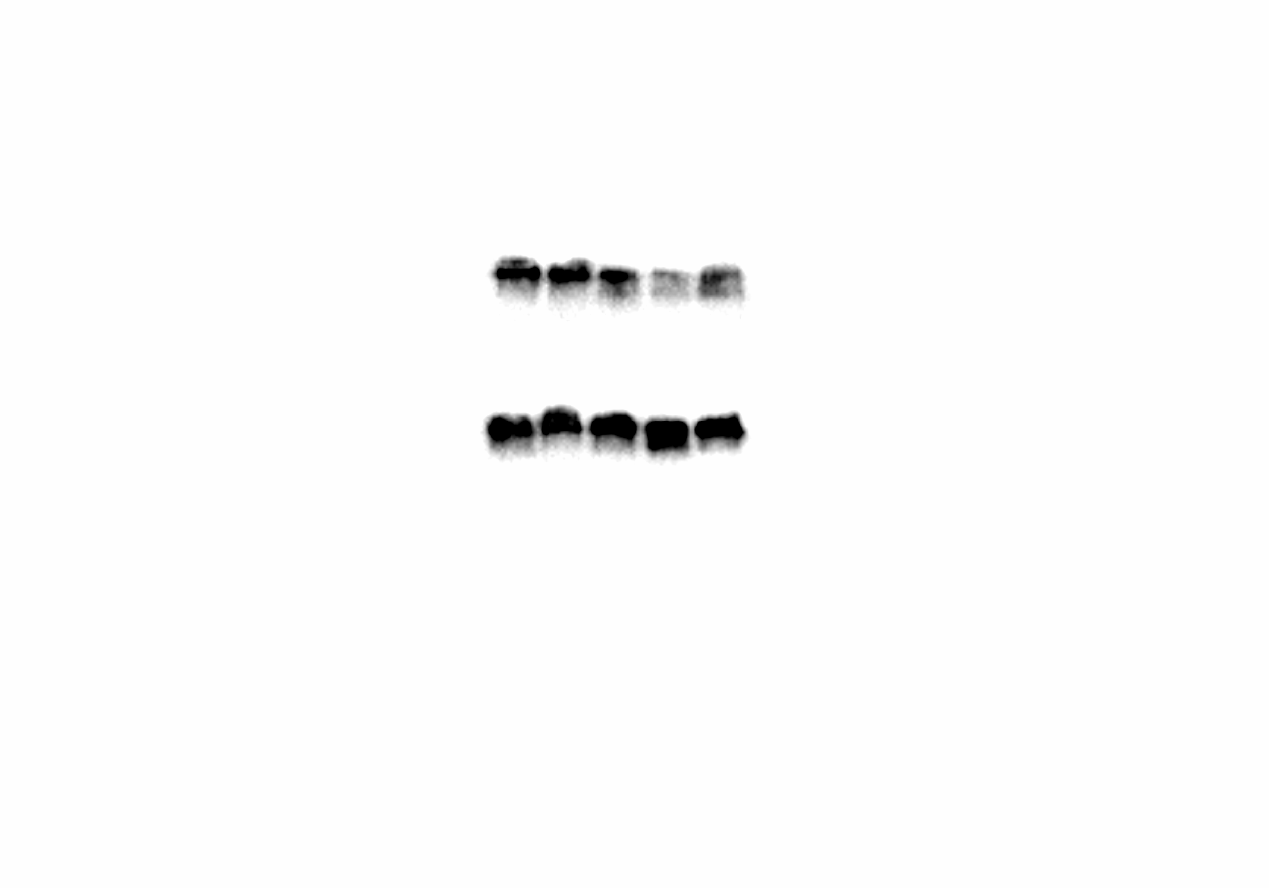


GLI1-IGF2BP2 OE

0h 2h 4h 6h 8h


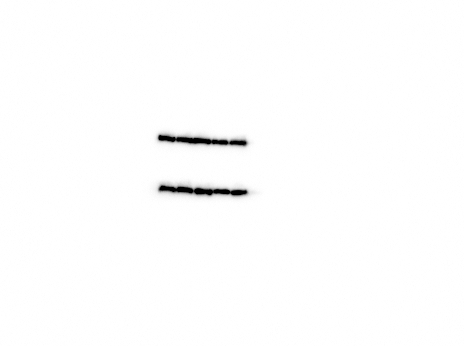


α-Tublin-IGF2BP2 OE

0h 2h 4h 6h 8h

FIGS4B


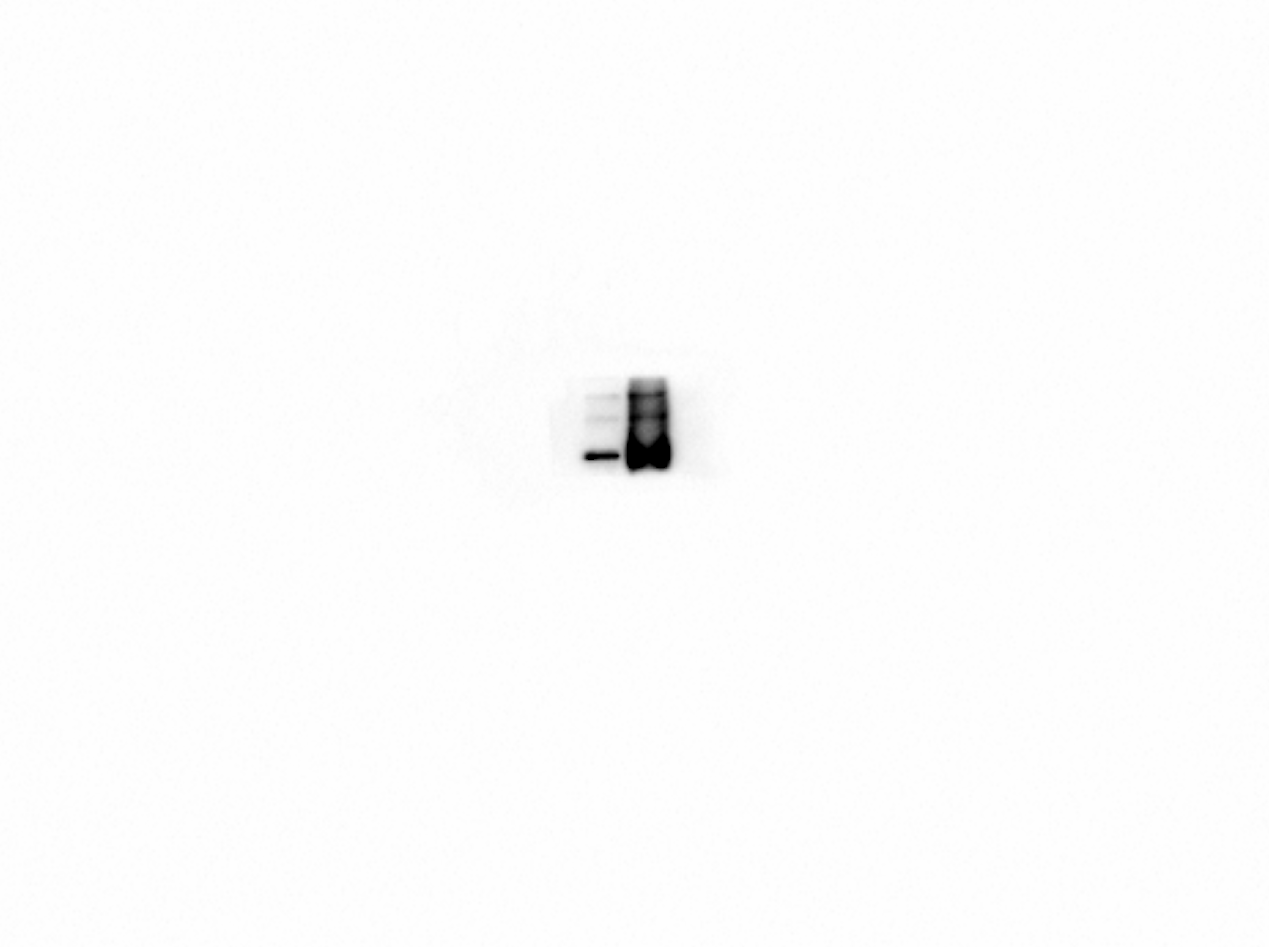


RPL22

con468

RPL22


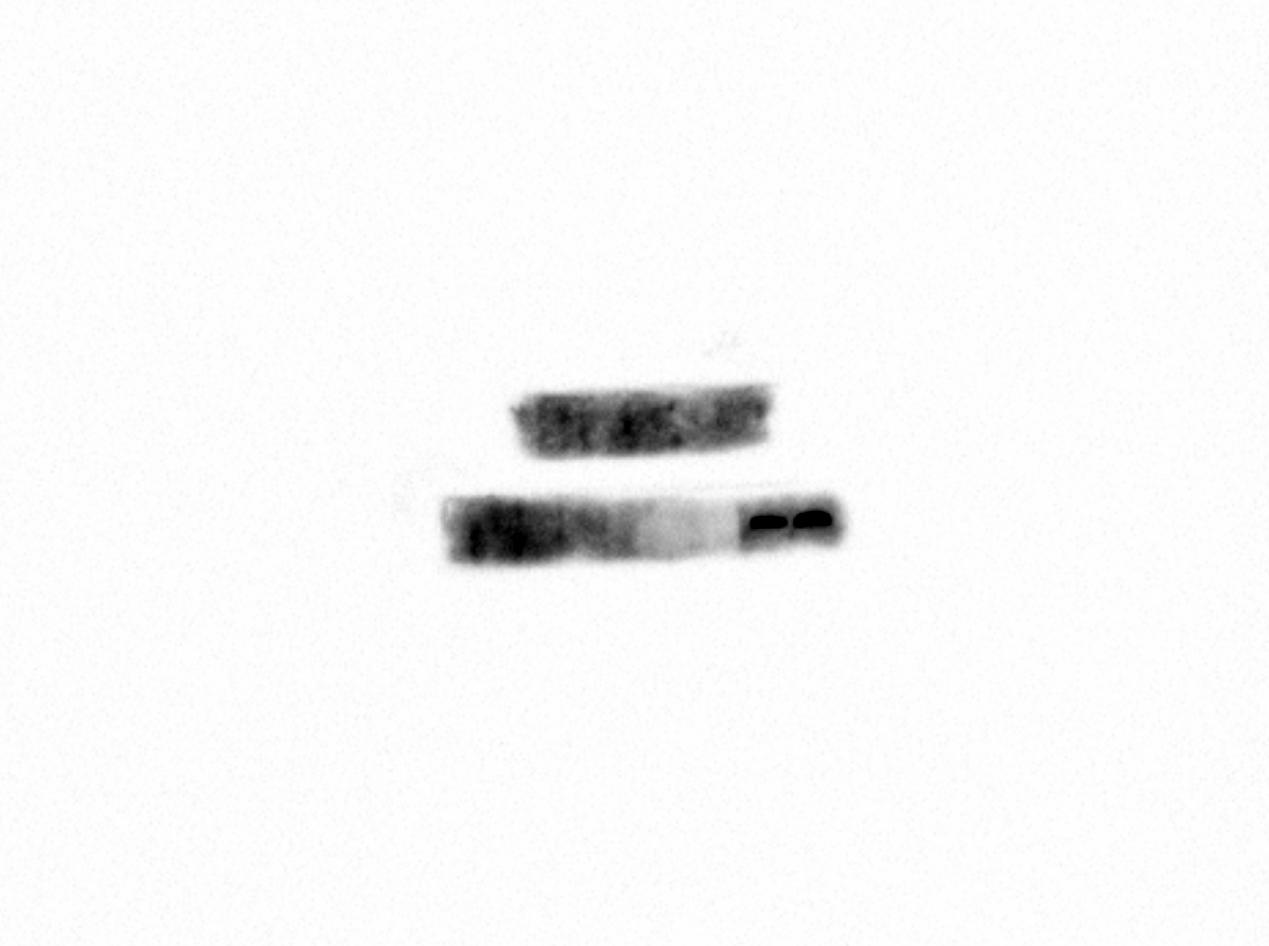


α-Tublin

con468

RPL22
